# A Semi-Supervised Approach for Refining Transcriptional Signatures of Drug Response and Repositioning Predictions

**DOI:** 10.1371/journal.pone.0139446

**Published:** 2015-10-09

**Authors:** Francesco Iorio, Roshan L. Shrestha, Nicolas Levin, Viviane Boilot, Mathew J. Garnett, Julio Saez-Rodriguez, Viji M. Draviam

**Affiliations:** 1 European Molecular Biology Laboratory–European Bioinformatics institute, Wellcome Trust Genome Campus, CB10 1SD, Cambridge, United Kingdom; 2 Department of Genetics—University of Cambridge, Downing Street, CB2 3EH, Cambridge, United Kingdom; 3 Cancer genome project–Wellcome Trust Sanger Institute, Wellcome Trust Genome Campus, CB10 1SD, Cambridge, United Kingdom; 4 RWTH-Aachen University Hospital, Templergraben 55, 52062, Aachen, Germany; Univesity of Texas Southwestern Medical Center at Dallas, UNITED STATES

## Abstract

We present a novel strategy to identify drug-repositioning opportunities. The starting point of our method is the generation of a signature summarising the consensual transcriptional response of multiple human cell lines to a compound of interest (namely the *seed compound*). This signature can be derived from data in existing databases, such as the connectivity-map, and it is used at first instance to query a network interlinking all the connectivity-map compounds, based on the similarity of their transcriptional responses. This provides a drug neighbourhood, composed of compounds predicted to share some effects with the seed one. The original signature is then refined by systematically reducing its overlap with the transcriptional responses induced by drugs in this neighbourhood that are known to share a secondary effect with the seed compound. Finally, the drug network is queried again with the resulting refined signatures and the whole process is carried on for a number of iterations. Drugs in the final refined neighbourhood are then predicted to exert the principal mode of action of the seed compound. We illustrate our approach using paclitaxel (a microtubule stabilising agent) as seed compound. Our method predicts that glipizide and splitomicin perturb microtubule function in human cells: a result that could not be obtained through standard signature matching methods. In agreement, we find that glipizide and splitomicin reduce interphase microtubule growth rates and transiently increase the percentage of mitotic cells–consistent with our prediction. Finally, we validated the refined signatures of paclitaxel response by mining a large drug screening dataset, showing that human cancer cell lines whose basal transcriptional profile is anti-correlated to them are significantly more sensitive to paclitaxel and docetaxel.

## Introduction

In the last few years, gene expression *signature matchin*g strategies have proven to be effective in identifying unexpected connections between transcriptional profiles of diseases and drug responses, based on genome-wide similarity metrics applied to gene expression data [1]. Using publicly available transcriptional data, derived either from general gene-expression data resources such as GEO [2] and ArrayExpress [3], or from more specific ones such as the Connectivity-map (a data-set of expression profiles derived from treatments with a large number of drugs on a set of human cell lines) (cMap) [4], it has been possible to infer new potential therapeutic applications for already approved drugs (drug- repositioning) [5].

Starting from the idea that each perturbation affecting the transcriptional activity of the cell can be summarised by a given gene expression signature, two computational paradigms have been conceived [6]. The first paradigm is based on the *signature reversion* principle and aims at identifying drugs inducing a transcriptional response anti-correlated (opposite) to that of a given disease. Drugs identified with this approach are hypothesized to be capable of reverting the disease signature, and hence the disease phenotype. This idea has been successfully applied in various contexts, including Crohn’s disease [7], skeletal muscle atrophy [8], cancer [9–11], and Alzheimer’s disease [12].

The second paradigm is based on the *guilt by association* principle, which assumes that if two drugs elicit a similar transcriptional response across a panel of heterogeneous human cell lines, they could share a mode of action (MoA), even if directly binding different intracellular targets. Hence, if one drug has a therapeutic effect for a certain disease, it is reasonable to hypothesize that the second drug could exert that effect too. Based on this idea, a ‘drug similarity network’ (DN) was assembled by systematically comparing transcriptional responses to drug treatment from the cMap database [13]. Cluster analysis of this network revealed groups of densely interconnected drugs enriched for a common MoA, and was used to investigate the MoA of new compounds, as well as to detect unreported effects of well-known drugs that are already contained in it. For example, through this approach fasudil, a drug used to reverse blood vessel spasm, was found to be an effective inducer of cellular autophagy [14]. With a similar method, comparison of the transcriptional signature of MT7, a compound that arrests cells in mitosis and disrupts spindle formation, to the cMap drug signatures predicted that MT7 is a microtubule perturbing agent [15].

**Fig 1:**
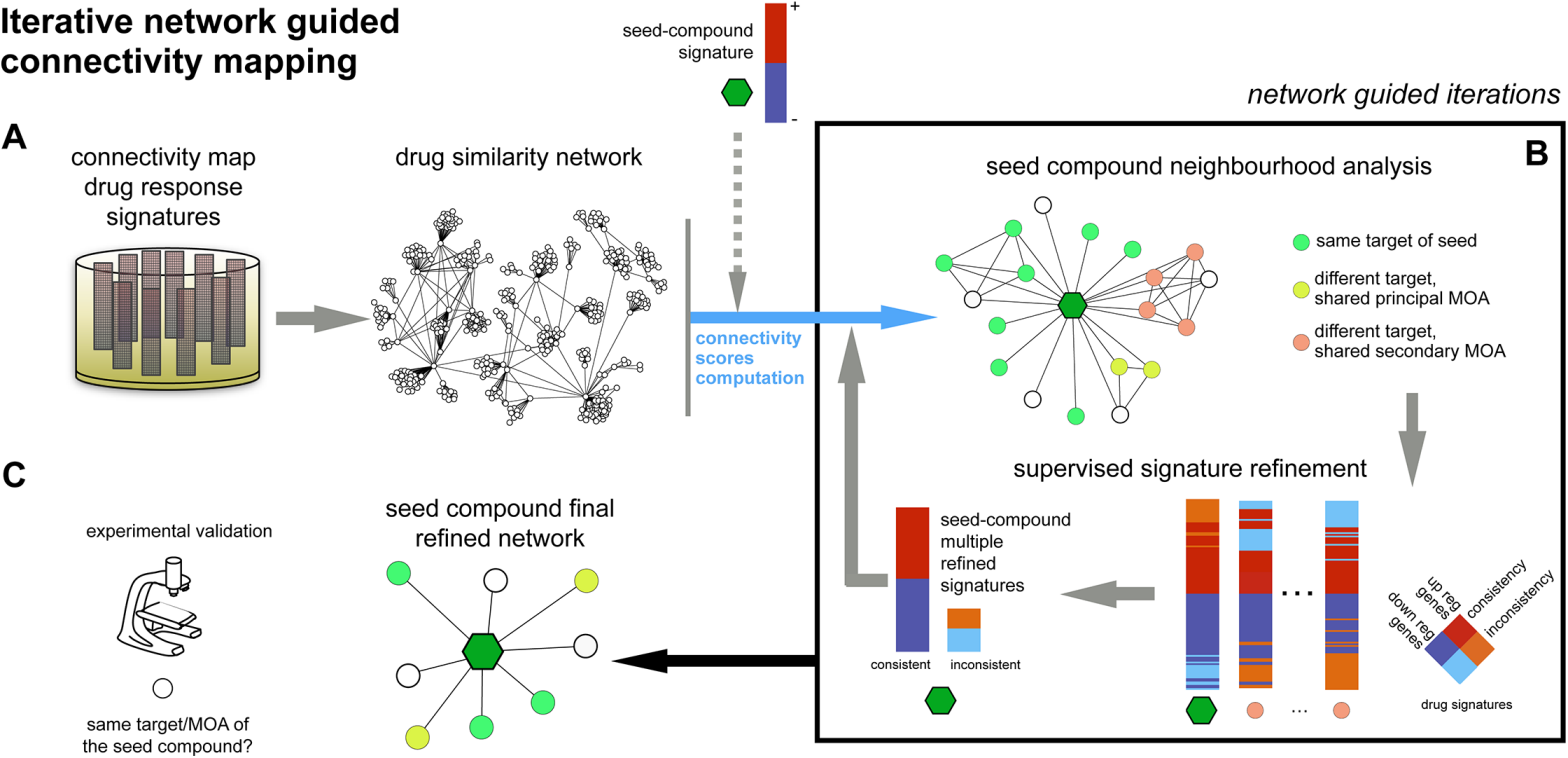
Iterative network guided connectivity mapping pipeline. (A) A drug similarity network (DN) is assembled from the drug response signatures contained in the connectivity-map database; the DN is queried by using the transcriptional signature of a seed compound, composed by up- and down-regulated genes, indicated in red and blue respectively, following treatment with the seed compound. (B) The resulting neighbourhood is analysed. By using a supervised approach the drugs and drug communities connected to the seed compound are investigated for enriched modes of action (MoAs); to dilute effects on drug similarity due to commonalities in secondary MoAs, refined signatures are computed, taking into account inconsistent effects of drug treatment on transcription (i.e. genes up-regulated by the seed treatment and down-regulated when treating with the classes of compounds characterised by the common secondary MoA and vice versa, indicated in orange and cyan respectively). This subdivides the original seed signature into a refined consistent signature and a refined inconsistent signature; the drug network is queried again with the computed multiple refined signatures and resulting connection scores are combined yielding a new seed neighbourhood; (C) after a number of refinement iterations the final neighbourhood of the seed compound is considered as output of the procedure; compounds in the final network, predicted to share the principal MoA of the seed compound, are finally selected for experimental validation.

Here we propose an approach that, starting from the computational pipeline proposed in [13], allows a supervised refinement of gene expression signatures following drug treatment to disentangle them from spurious effects due to drugs’ secondary effects. Particularly, we use the DN of [13] as initial searching space to identify molecules eliciting a transcriptional response similar to that of a compound of interest, that we call the *seed compound* (SC) (Fig 1A). This analysis yields a neighbourhood of compounds connected to the SC and clustered into *network communities* [16] statistically enriched for certain MoAs (Fig 1B). Among these, groups of drugs not having the same principal MoA of the SC but sharing a secondary effect with it can be found. For example, Hsp90 inhibitors are densely interconnected in the DN to proteasome inhibitors. This happens because, even if having different direct targets, these two classes of compounds increase the abundance of unfolded proteins. In fact, compounds in the first class inhibit one of the most important chaperone proteins (involved also in protein degradation), whereas compounds in the second class inhibit an enzyme responsible for the degradation of misfolded/unfolded proteins. As a consequence Hsp90 inhibitors and proteasome inhibitors both up-regulate genes involved in the response to the unfolded protein stress, and this results into a strong similarity at the transcriptional response level between these two classes of compounds [13,17]. For similar reasons, Topoisomerase I and II inhibitors and Cdk2 inhibitors are densely interconnected in the DN because of their common effect on cell cycle mediated by the inhibition of endogenous CDKs via the up-regulation of p21 (which follows the DNA damage) in the first case, and by the direct inhibition of Cdk2 in the second case [13,18]. These examples highlight that the definition of relevant primary MoA and secondary (non relevant) MoA is not general and varies across classes of drugs, with level of granularities that must be necessarily defined run-time by the user in a supervised and fully tunable step of analysis.

To filter out the effect of secondary MoAs of two drugs on their similarity we designed an iterative, network-guided approach for disentangling the transcriptional changes due to a primary MoA of interest from those changes induced non-specifically through secondary effects (Fig 1B). This is obtained by systematic reducing the overlap between the signature of the SC and those of neighbouring compounds that share a known secondary MoA with it. Hence, our methods combines an unsupervised step (the query of the drug network), with a supervised one where we take into account of a priori known characterisation of the drugs in the resulting network neighbourhood, and we use this information to refine the query signatures.

By applying two iterations of this method, and using paclitaxel (a microtubule inhibitor) as seed compound, we predicted two novel microtubule perturbing agents: splitomicin, an inhibitor of SIRT2 that deacetylates tubulin [19] and glipizide, a short acting anti-diabetic drug possessing hypoglycemic properties [20]. We experimentally verified that treating cells with splitomicin and glipizide induces microtubule stabilization, by measuring microtubule growth rates and microtubule turnover with levels of acetylated tubulin (Fig 1C). Both splitomicin and glipizide treatments induced subtle perturbations to both chromosome congression and segregation, supporting their roles as agents that perturb microtubule function. Finally, we validated the ability of the generated refined signatures in predicting sensitivity to paclitaxel, docetaxel, and vinorelbine, in terms of reduced cell viability across ~600 human cancer cell lines, from a high-throughput drug screening (http://www.cancerrxgene.org/), [21,22].

To allow easy implementation of our pipeline, we provide a set of free, open-source R scripts and objects that implement our code and reproduce our results, available at: https://github.com/francescojm/iNRG_cMap and http://www.ebi.ac.uk/~iorio/PLoS_ONE_Submission. Thus, this proof of principle study, together with the public available code implementing our computational pipelines, provides opportunities for improving drug-repositioning predictions through a network-guided iterative approach.

## Results

### A gene-signature summarizing the effect of paclitaxel in human cancer cell lines

We assembled a consensual transcriptional response for each drug *X* contained in the cMap [17] using the approach described in [13,18], and detailed in the Methods section. This response summarizes the consistent effect of an *X*-treatment on transcription across all the treated cell lines contained in the study, in the form of a genome-wide ranked list of genes (i.e. ‘Prototype Ranked List’ (PRL-signature)—S1 Supplementary Dataset, available at https://github.com/francescojm/iNRG_cMap/ and at http://www.ebi.ac.uk/~iorio/PLoS_ONE_Submission). Then we used the paclitaxel PRL-signature as a seed to query the drug network described in the same study (Fig 1A). This network summarizes the effects of 1,309 bioactive small molecules. Drugs are grouped according to their PRL-signature similarity, in a hierarchical topology where different regions contain modules of drugs enriched for a given MoA.

**Fig 2:**
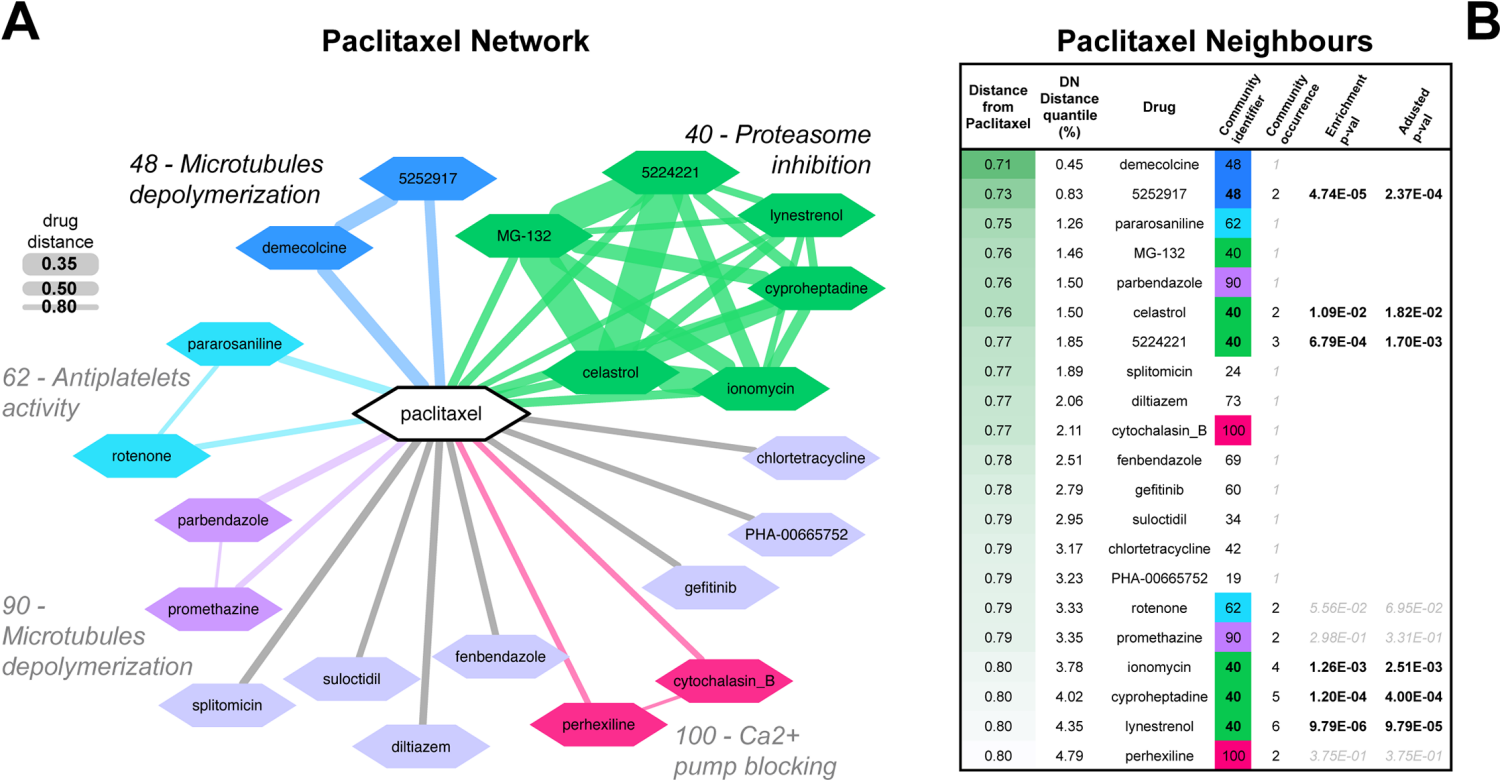
Paclitaxel initial network. (A) The initial neighbourhood of paclitaxel in the drug similarity network (DN). Each node represents a drug whose consensual transcriptional response is significantly similar to that elicited by paclitaxel. Edge thickness is proportional to the distance between the connected drugs and different colours indicate different drug communities. In the inset some of the mode of action enriched in the communities are reported. (B) List of drugs contained in the paclitaxel neighbourhood. First column contains drug distance scores between paclitaxel and the drug under consideration. The second column contains the percentile in which the distance falls when sorting all the possible pair-wise distances between the 1,309 drugs in the cMap dataset. Third column contains the name of the drug while the fourth column contains the identifiers of the network community containing the drug under consideration. Fifth column contains the occurrence of that community when considering the first *n* neighbours (where *n* is the position in the list). Sixth and seventh columns contains, respectively community enrichment *p-values* (i.e. the probabilities of observing a given number of drug from a given community, in the first *n* neighbours given the total number of drugs belonging to that community in the whole DN) and the same p-values after correction for multiple hypothesis testing.

Our analysis was focused on the paclitaxel neighbouring drugs and modules in the network (Fig 2). These drugs elicit a transcriptional response similar to that of paclitaxel, according to the distance metric described in the supplementary methods and in [13], hence they could share a mode of action (MoA) with paclitaxel. We performed a statistical enrichment analysis quantifying how surprising it is to observe occurrences of drugs from a given community in the paclitaxel neighbourhood (Fig 2B and S1 Table). The drug most similar to paclitaxel is demecolcine (drug distance (D) = 0.71, in the 0.45% distance quantile), a microtubule-depolymerizing drug that at low doses binds to microtubule plus-ends to inhibit its dynamics. Additionally, in the paclitaxel neighbourhood some known microtubule destabilizers (such as celastrol, parbendazole, and fenbendazole) are present; the significantly recurrent communities are number 40 (p-value = 6.95 × 10^−6^), enriched in the original DN for proteasome inhibitors (PI) and other mitotic progression blockers, and number 62, which in the network contains a mixture of calcium channel blockers, anti-infective agents and anti-platelet compounds. The full composition of these communities in the network is provided in S1 Table. In conclusion, proteasome inhibitors, microtubule destabilisers and stabilisers, all capable of arresting cells in mitosis, were identified in the paclitaxel neighbourhood.

The paclitaxel optimal-signature used to query the network (supplementary methods) is composed of the top 250 transcripts that are consistently up- and down-regulated in the cMap (S2 Table). The change in cell cycle regulated genes, such as CCNE2 down-regulation [23] and CENPE up-regulation [24], and enrichment of gene ontology categories (GO:terms) [25] such as ‘establishment or maintenance of cytoskeleton polarity’ and ‘microtubule perturbation’, ‘kinetochores’, ‘chromosome segregation’ and ‘chromosome condensation’ (S1 Table), are all consistent with transcriptional states of a mitotic arrest in cells as expected to occur from microtubule stabilization.

Taken together these results confirm that the paclitaxel optimal-signature: (i) is informative of the paclitaxel MoA; (ii) might be suitable for the identification of new microtubule stabilising agents through signature-matching strategies; and (iii) could be a first step towards the characterization of a regulatory network of microtubule stabilisation but (iv) could also be entangled with secondary effects, such as mitotic arrest.

### Iterative network-guided connectivity-mapping predicts new microtubule stabilizing agents

**Fig 3:**
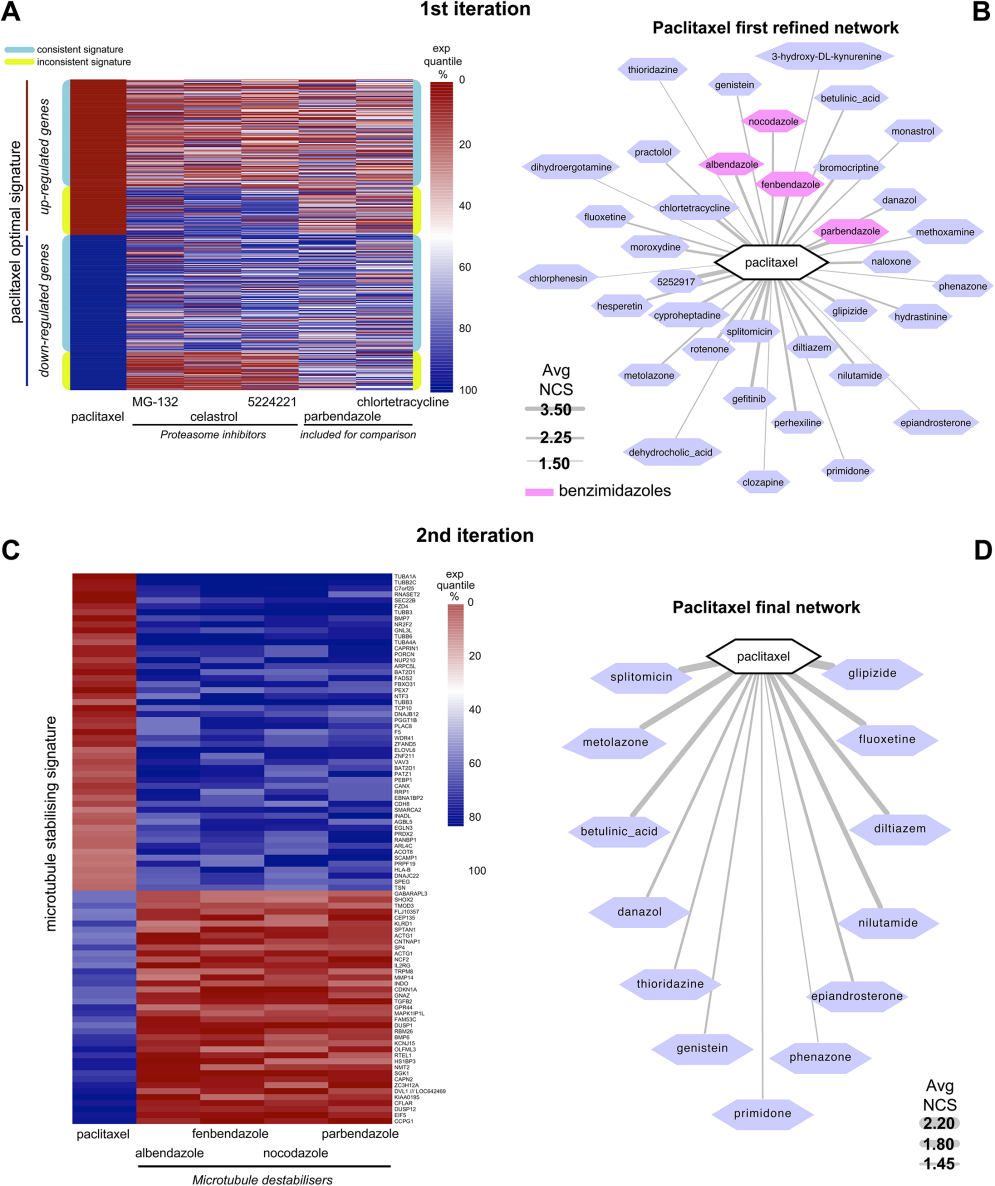
Paclitaxel refined networks. (A and B) First iteration of the network guided connectivity-mapping: (A) Expression percentile heat-map for the genes in the paclitaxel optimal-signature, along the prototype ranked lists (PRLs) of paclitaxel and some of its neighbouring drugs: 3 proteasome inhibitors (PIs) and 2 benzimidazoles (included for comparison). Rows corresponding to genes whose rank positions are inconsistent between the PRLs of paclitaxel and PIs (Paclitaxel/PIs inconsistent signature) are highlighted in yellow while those corresponding to genes with consistent rank positions are highlighted in cyan. (B) Network of drugs connected to paclitaxel when querying the connectivity-map (cMap) with the multiple signatures highlighted in the heat-map. Each node represents a drug whose consensual transcriptional response is significantly similar to the computed multiple signatures simultaneously, edge thickness is proportional to the normalised connectivity scores (NCS) between the connected drugs. Recovered microtubule perturbing drugs are highlighted in pink. (C and D) Second iteration of the network guided connectivity-mapping: (C) Expression percentile heat-map for the genes in the microtubule stabilising signature, along PRLs of paclitaxel and the 4 recovered benzimidazoles. (D) Network of drugs connected to paclitaxel when querying the cMap with the multiple signatures in the two heat-maps. The graphic notation is the same of (B).

Two lines of evidence suggested that the paclitaxel optimal-signature might not only reflect the transcriptional response to paclitaxel induced microtubule stabilisation but also include secondary effects due to paclitaxel induced mitotic arrest. First, the GO:term enrichment analysis of the paclitaxel optimal-signature highlighted the up-regulation of genes associated with the G2-M phase of the cell cycle. Second, drugs in community number 40 are among the most recurrent ones in the paclitaxel neighbourhood (p-value = 6.95 × 10^−6^), and this community is enriched for proteasome inhibitors such as celastrol and MG-132, which induce mitotic arrest [26]. To disentangle the specific effects of paclitaxel on microtubule stabilisation from the secondary effects arising due to paclitaxel induced mitotic arrest, we focused on three typical mitotic progression blockers and proteasome inhibitors predicted to be very similar to paclitaxel in the network based on our previous analysis: MG-132, celastrol and 5224221 (all contained in community n. 40) (Fig 2). Although comparing the PRL-signatures of these three drugs with the paclitaxel optimal-signature showed an overall consistent profile of expression, reflecting the similarity of these drugs in the network, we also observed a number of genes that showed an inconsistent profile across the two classes of drugs (Fig 3A and S1 Fig). Particularly, we identified a subset of genes behaving inconsistently across paclitaxel and proteasome inhibitor treatment and collected them into an inconsistent-signature composed by two groups of genes, defined as follows (and highlighted in yellow in the heat-map shown in Fig 3A):

(A) Up-regulated set, containing genes in the up-regulated part of the paclitaxel optimal-signature but falling above the 70th percentile in the PRL-signature of at least two drugs among MG-132, celastrol and 5224221;
(B) Down-regulated set, containing genes in the down-regulated part of the paclitaxel optimal-signature but falling within the 30th percentile of the PRL-signature of at least two drugs among MG-132, celastrol and 5224221.

The two lists of genes are in S3 Table. As we did for the paclitaxel optimal-signature, we performed a GO enrichment analysis on the paclitaxel/PIs inconsistent signature and the results were very similar to those of the previous case (S3 Table). Despite the reduction in the number of considered genes, most of the enriched terms were still linked to microtubules, spindle, spindle pole, chromosome condensation and segregation, indicating that this refined signature is more specifically linked to the effect of paclitaxel on microtubule stabilisation, with respect to the paclitaxel optimal-signature, which apparently covers a wider range of effects.

To better account for the effects on microtubule stabilisation, and to add more weight to the contribution of the expression-changes of these inconsistently modulated genes on the initial connectivity scores, we used the two refined signatures, respectively the paclitaxel/PIs consistent and inconsistent signatures, to compute individual connectivity scores to the cMap drugs, using the method implemented on the cMap query tool [17] and combining the obtained results as follows. To avoid the problem of data comparability across the different batches in the cMap [27,28], and to dilute cell line specific effects on drug response, we used the PRL-signatures rather than individual gene expression profiles [13]. We derived connectivity scores between the two signatures and all the cMap drug PRL-signatures using gene set enrichment analysis (GSEA) [29] (Fig 3B and S4 Table). To compute the final refined paclitaxel neighbourhood, the normalised connectivity scores of the compounds yielding a positive connection with a false discovery rate (fdr) < 5%, when queried with both the signatures, were averaged. Finally, the compounds were sorted according to their average score. This resulted in a list of 36 compounds, further referred to as the paclitaxel-refined network (Fig 3B and S4 Table).

Compared to the initial paclitaxel network (Fig 2A) the refined network contains more drugs; additionally, some benzimidazoles (well established microtubule destabilisers) such as albendazole and nocodazole are recovered from the cMap. Importantly, proteasome inhibitors are no longer connected to paclitaxel. This suggests that the similarity accounted while composing this network is disentangled from commonalities to mitotic arrest and probably reflects more efficiently the effects of the analysed drugs on microtubules.

To reduce the secondary effect of gross microtubule disruption from specific effect of microtubule stabilisation, we iterated this procedure by discriminating microtubule stabilisers and destabilisers in the paclitaxel-refined network. We focused on the 4 benzimidazoles, known to destabilise microtubules, contained in the paclitaxel-refined network: parbendazole, fenbendazole, albendazole and nocodazole (in order of similarity to paclitaxel). We computed a microtubule-stabilising signature (Fig 3C) selecting the genes that are specifically up-regulated upon treatment with paclitaxel and down-regulated upon treatment with the benzimidazoles, and vice-versa. Then we checked for connections of the PRL-signature of the drugs in the paclitaxel-refined network with the microtubule-stabilising signature. This allowed us to predict the likelihood of a drug as a microtubule stabiliser versus destabiliser on the basis of positive and negative connectivity scores, respectively. In this way, we computed a connectivity score quantifying how likely it is that a drug stabilises microtubules (positive score) or destabilises them (negative score) (Table 1). We combined these scores (after normalisation) with those resulting from the previous analysis by additionally refining the paclitaxel network (Fig 3D and Table 1).

**Table 1:** Paclitaxel final network.

DRUG	Paclitaxel/Proteasome Inh. Consistent Signature NCS	Paclitaxel/Proteasome Inh. Inconsistent Signature NCS	Microtubule Stabilisation Signature NCS	Avg NCS
glipizide	3.29	2.19	**1.14**	2.20
splitomicin	3.65	2.45	**0.03**	2.05
fluoxetine	2.72	1.60	**1.60**	1.97
metolazone	2.16	2.15	**1.34**	1.88
5252917	4.30	2.72	**-1.63**	1.80
diltiazem	3.56	1.74	**0.07**	1.79
betulinic_acid	2.71	1.62	**1.01**	1.78
nilutamide	2.93	1.89	**0.45**	1.75
moroxydine	2.61	2.47	**-0.24**	1.62
perhexiline	3.42	1.60	**-0.30**	1.57
gefitinib	3.53	2.23	**-1.15**	1.54
bromocriptine	3.16	2.14	**-0.75**	1.52
rotenone	3.85	1.71	**-1.09**	1.49
danazol	2.58	1.79	**0.05**	1.47
epiandrosterone	1.74	1.53	**1.14**	1.47
chlortetracycline	3.43	2.23	**-1.38**	1.43
thioridazine	1.88	1.53	**0.74**	1.38
cyproheptadine	3.62	1.67	**-1.20**	1.36
hydrastinine	2.51	1.77	**-0.21**	1.36
genistein	1.98	2.04	**0.01**	1.34
naloxone	2.93	2.13	**-1.15**	1.30
monastrol	2.09	1.78	**-0.05**	1.27
hesperetin	3.16	1.63	**-1.01**	1.26
primidone	1.87	1.82	**0.10**	1.26
phenazone	1.74	1.64	**0.27**	1.22
dehydrocholic_acid	1.80	1.86	**-0.71**	0.98
methoxamine	1.96	1.88	**-0.96**	0.96
3-hydroxy-DL-kynurenine	1.76	1.92	**-0.95**	0.91
practolol	2.38	1.76	**-1.51**	0.88
chlorphenesin	1.57	1.53	**-0.93**	0.73
dihydroergotamine	1.68	1.59	**-1.64**	0.54

We performed a GO enrichment analysis on the genes contained in the microtubule stabilising signature. To cover a wider range of effects on transcription and obtaining a signature of suitable size, the microtubule-stabilising signature (S5 Table) was composed of genes falling within the 25% quantile along the paclitaxel PRL-signature and over the 75% quantile along the PRL-signatures of the 4 benzimidazoles, and vice versa. Over-represented GO:terms linked to protein polymerization and microtubules, when considering the up-regulated part of the signature, and response to oxygen levels and hypoxia, when considering the down-regulated one (S5 Table).

Among the 36 drugs contained in the paclitaxel-refined network, 9 had a positive score when queried with the microtubule stabilising signature (hence are predicted to stabilise microtubules, as shown in Table 1). When sorting them according to the finally averaged scores, glipizide ranked first, followed by splitomicin, fluoxetine, metolazone, 5252917, diltiazem, betulinic acid, nilutamide, moroxydine and perhexiline.

Some of these have already been linked to processes related to microtubule function: Flouxetine modulates microtubular system in rat hippocampus [30]; 5252917 is shown as a tubulin active agent in [31]; diltiazem is known to augment paclitaxel and proven in clinical trial as a combinatorial drug that could reduce the dosage of colchicine (another microtubule stabiliser) [32]; Nilutamide targets the Androgen receptor that relies on microtubule dynamics for signalling and transport [33].

Overall, the lack of prominent enrichment of mitotic genes and related GO:terms in the microtubule stabilising signature suggested the possibility that our matching strategy is no longer reflecting microtubule stabilisation induced cell cycle arrest alone. This is further confirmed by the up-regulation of tubulin genes that would be expected as a cellular response to depletion of tubulin subunits arising from microtubule stabilisation. Hence, the final list of compounds could contain drugs whose impact on microtubule function is very similar to that exerted by paclitaxel. We chose to validate glipizide and splitomicin as they ranked in the top-2 positions in the final list. In agreement with the guilt by association principle mentioned in the introduction, although no direct interaction with tubulin has been reported for splitomicin and glipizide, our results suggest that they can indirectly induce microtubule stabilisation.

### Experimental validation of Glipizide and Splitomicin as microtubule stabilisers

Glipizide is an anti-diabetic drug and a potassium channel antagonist [34] which has not been previously associated with microtubule stabilisation, although potassium channel BKCa is known to directly interact with Microtubule Associated Protein 1A (MAP1A) in both excitable and non-excitable cells [35]. Splitomicin a well-studied inhibitor of yeast Sir2 which does not inhibit the human SIRT homolog SIRT1 [36,37] but potentiates the anti-motility activity of taxanes [38]. Human SIRT2 is known to deacetylate tubulin, in a cell type dependent manner [19,39]. Tubulin acetylation occurs on microtubules that are turned over slowly (hence is a marker of stable and long-lived microtubules) [40], although tubulin acetylation per se does not directly confer microtubule stability [41]. In here, we tested if glipizide and splitomicin are able to directly or indirectly induce microtubule stabilisation as predicted through our study.

**Fig 4:**
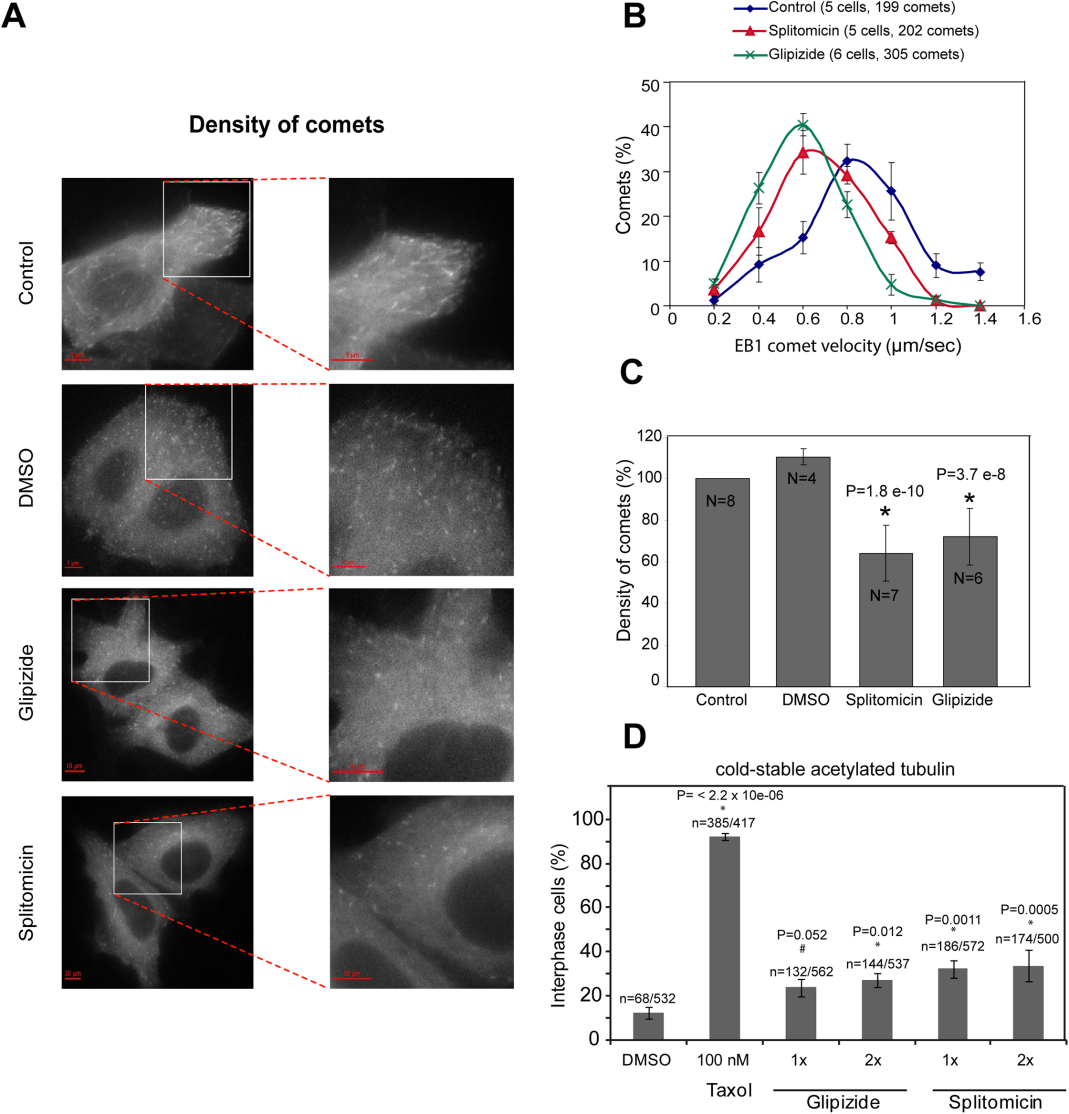
Splitomicin and Glipizide treatment reduce the incidence of microtubule growth and density and increase the amount of acetylated tubulin. (A) Live-cell images of HeLa^EB3-TdTomato^ cells treated with 40 **μ**M glipizide or 20 **μ**M splitomicin, as indicated. HeLa^EB3-TdTomato^ expressing cells were treated with the indicated drugs for 1 h and filmed using time-lapse microscopy once every 10 seconds for a period of 5 minutes. Scale bar as indicated. (B) Graph shows drug treatment induced reduction in EB3 comet velocity. Instantaneous velocity of EB3 comets between consecutive time-frames were considered using movies acquired as described in (A). (C) Graph shows drug treatment induced reduction in EB3 comet density. Comet density plotted as percentage of EB3-comets observed per square μm area in interphase cells. Values are normalised using unperturbed negative control conditions. N = number of cells. (D) Graph showing increased incidence of cold stable and acetylated tubulin bearing microtubules following drug treatments. Cells were treated with two different concentrations of glipizide (20 μM; 1x and 40 μM; 2x) or splitomicin (10μM; 1x and 20μM; 2x) and cold treated as described in S2A prior to immunostaining with antibody against acetylated tubulin. * indicates statistically significant difference assessed using binomial confidence interval.

To assess the extent to which the two drugs may suppress microtubule dynamics, we used two approaches: first, we measured the velocity and density of growing microtubule-end bound protein, EB3 fused to TdTomato (EB3-TdTomato), using live-cell imaging (Fig 4A) and second, we measured the amount of acetylated tubulin, a post-translational marker for long-lived stable microtubule populations, using fixed-cell immunofluorescence (Fig 4D). Following an hour of drug treatment, the velocity and density of EB3 comets in interphase HeLa EB3-TdTomato cells were significantly reduced compared to control cells (Fig 4B and 4C). Under similar conditions, 100 nM paclitaxel treatment would abolish almost all of EB3 comets (NT and VMD, unpublished data). These data indicate two points: first glipizide and splitomicin treatments can reduce microtubule growth rates and suppress microtubule growth fraction, indicative of microtubule stabilization. Second, compared to paclitaxel, the impact of glipizide and splitomicin are mild, highlighting the strength of the signature in exposing drugs with a microtubule stabilising ability even when this is not as high as that of paclitaxel.

To confirm the above finding, we measured the level of acetylated tubulin, a marker of long-lived stable microtubules. We exposed drug-treated cells to a brief cold treatment that disassembles dynamic microtubules and then fixed cells for immunostaining with anti-acetylated tubulin and anti-tubulin antibodies (S2 Fig). Immunostaining studies showed a slight but reproducible increase in the proportion of cells positive for acetylated tubulin following drug treatment, compared to control DMSO treatment (Fig 4D and S2(B) Fig); in contrast, no noticeable difference was observed in total tubulin intensities between drug treated and untreated populations (data not shown). This confirms that glipizide and splitomicin increase the incidence of stable and long-lived microtubules in cells. In summary, both EB3-comet behaviour and acetylated tubulin status show a role for glipizide and splitomicin in altering microtubule stability, consistent with our predictions. Because glipizide and splitomicin were excluded from being microtubule increasing agents in a high-throughput screen (HCS assay for microtubule stabilizers, http://pubchem.ncbi.nlm.nih.gov/assay/assay.cgi?aid=2205), the role for glipizide and splitomicin in altering the state of microtubule dynamics is probably indirect.

### Cell division events are perturbed in the presence of glipizide and splitomicin

Although glipizide and splitomicin have not been reported as direct tubulin binding agents, our studies so far indicate a role for glipizide and splitomicin in altering the state of microtubule dynamics.

Microtubule stabilisation is expected to perturb chromosome congression and segregation during cell division. We tested if microtubules stabilisation following glipizide and splitomicin treatment perturbed mitosis. For this purpose, we performed two assays: first, we quantified the percentage of rounded-up cells (mitotic index) in HeLa cell populations treated with varying concentrations of glipizide and splitomicin for either a three or six hour period. Second, we performed time-lapse microscopy to follow mitosis progression in cells exposed to glipizide and splitomicin.

Following a three hour period of drug treatment we observed an increase in mitotic indices of splitomicin or glipizide similar to paclitaxel treated populations, indicative of a mitotic perturbation. However, following a six-hour drug treatment, there was only a modest increase in mitotic cells in splitomicin treated populations, compared to populations treated with paclitaxel (S3(A) Fig). This shows that mitotic arrest following glipizide and splitomicin treatment was not prolonged, a case similar to the mitotic delay but not terminal arrest observed following the depletion of a potent microtubule destabilizer, MCAK [42,43]. In agreement, time-lapse microscopy movies of drug treated cells showed a slight delay in anaphase onset compared to controls (S3(B) Fig). To investigate if the cause for mitotic delay is related to defective microtubule function, we quantified the extent of chromosome congression and segregation defects in cells treated with varying concentrations of the two drugs. Analysing the presence of uncongressed chromosomes in metaphase cells showed a dose dependent increase in cells with congression defects following splitomicin and glipizide treatment ((S3C and S3D) Fig). A similar dose dependent increase in segregation defects in anaphase cells was also observed following treatment with splitomicin and glipizide alone ((S3C and S3E) Fig). Importantly, we find that splitomicin caused a more prominent increase in congression and segregation defects, compared to glipizide (S3(D) Fig). These findings on the differences in the severity of mitotic defects are consistent with the less severe reduction in comet density following glipizide treatment compared to splitomicin treatment.

The data from live-cell assays show that glipizide and splitomicin disrupt mitotic progression less severely compared to paclitaxel, indicating our approach to be sensitive enough for identifying compounds that are less potent compared to paclitaxel. Neither glipizide nor splitomicin cause a terminal mitotic arrest implying that our approach is capable of excluding the effects of a microtubule stabilization induced mitotic arrest phenotype.

### Cancer cell lines expressing refined signatures of microtubule instability are sensitive to paclitaxel, docetaxel and vinorelbine

**Fig 5:**
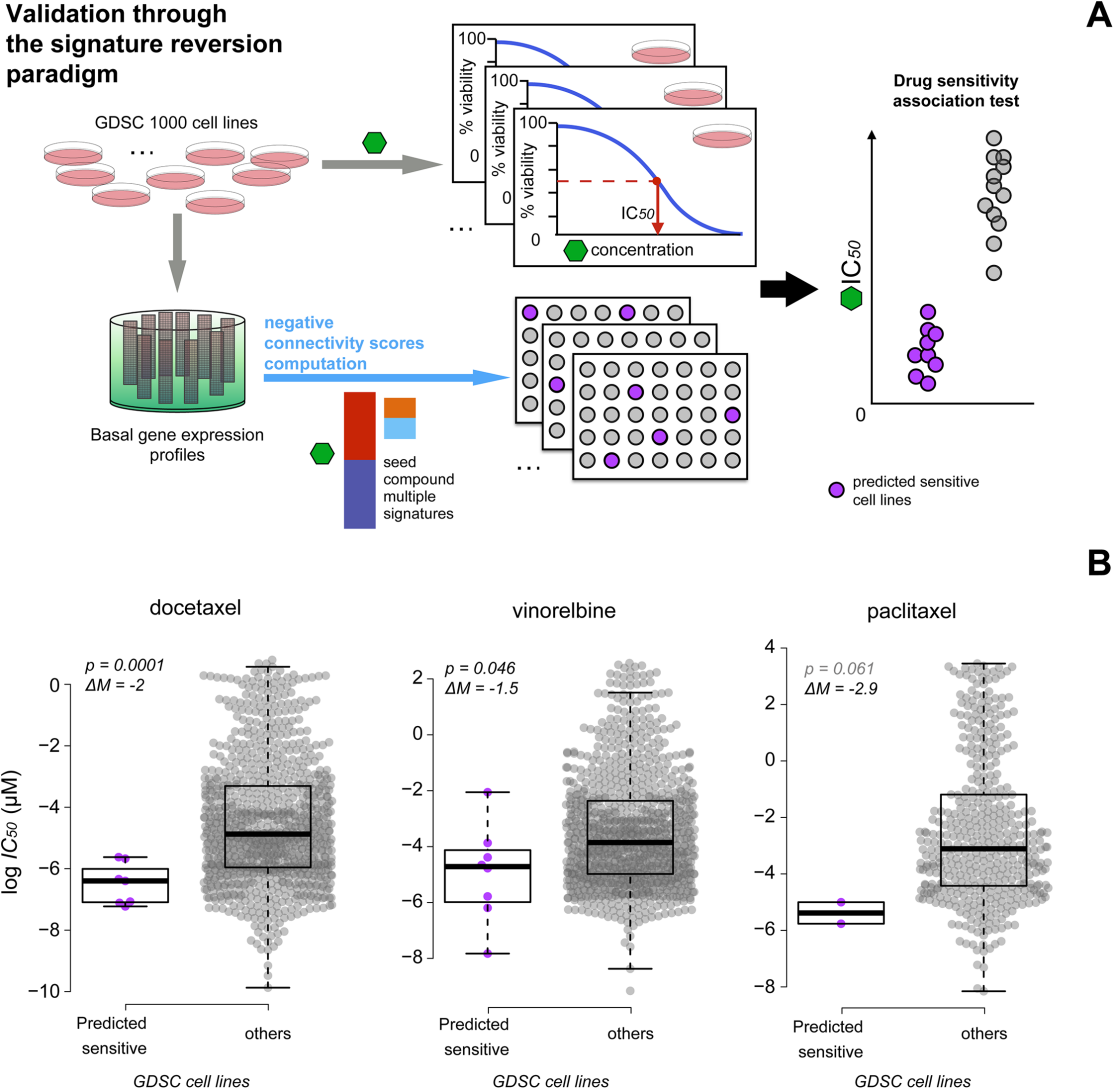
Validation of the predictive ability of the refined signatures. (A) Validation of the signatures through the signature reversion paradigm. The basal expression profiles of the cell lines in the GDSC panel are queried against the refined multiple signatures and connection scores are computed. Cell lines negatively connected to the signatures are predicted to be more sensitive to paclitaxel and its analogue, for which drug response data is available in the GDSC database. (B) Scatter plots showing that the cancer cell lines negatively connected to the Paclitaxel/PIs inconsistent signature and the microtubule stabilising signature (simultaneously) tend to be more sensitive to docetaxel, vinorelbine and paclitaxel. Purple points refer to cell lines significantly negatively connected to the two signatures for which drug response data is available. Gray points refer to the cell lines in the rest of the panel. Coordinates on the y-axis indicate the log IC*50* of the drug under consideration. P-values of an unpaired two sample t-test quantifying the extent of difference in drug response across the two groups of cell lines are reported in the insets together with the difference between the two log IC*50* means (ΔM).

As mentioned in the introduction, it has been shown that if a drug can revert the transcriptional signature of a given disease then it might able to revert the disease phenotype itself. If this hypothesis holds true for paclitaxel and drugs in the paclitaxel final neighbourhood, we would expect that the extent of inverse match between our refined transcriptional signatures for paclitaxel and the basal gene expression of cancer cell lines would correlate with their sensitivity (in terms of viability reduction) to microtubule stabilising drugs (Fig 5A). To test this hypothesis, we used a large-scale drug screening dataset from the Genomics of Drug Sensitivity in Cancer (GDSC) project [21]. For our analysis across the whole panel of screened cell lines, we extracted from the GDSC (http://www.cancerrxgene.org/, version #5, web-released in may 2014) database, IC50 (half-maximal inhibitory concentration) values and the area under the dose response curve (AUC), corresponding to treatments with paclitaxel, docetaxel (a taxane semi-synthetic analogue of paclitaxel), vinblastine (a vinca alkaloid), vinorelbine (a semi-synthetic vinca alkaloids) and epothilone B: all microtubule stabilisers. This data was then paired with the collection of basal expression profiles of the GDSC panel of cell lines, publicly available on ArrayExpress [3] (accession number: E-MTAB-783).

After pre-processing (supplementary methods) we computed connectivity scores of the GDSC cell lines versus the multiple refined signatures as explained in the previous sections (S6 Table). Ten cell lines were significantly negatively connected to both the paclitaxel/PIs inconsistent signature and the microtubule stabilising signature, hereafter referred as the predicted sensitive cell lines (S4 Fig). None of the cell lines in the GDSC panel were significantly negatively connected to all the three signatures simultaneously.

To test for association between the predicted sensitive cell lines and the level of sensitivity to the 5 drugs under consideration, we divided the whole panel of cell lines into two subsets: one containing the predicted sensitive cell lines and the other containing the rest of cell lines in the panel. We performed a two-sample unpaired t-test to check for differences in the average log IC50 values and the AUCs of the drugs under consideration. We found a clear (> 2-folds) and significant association between the predicted sensitive cell lines and increased sensitivity to docetaxel and vinorelbine when considering the difference in IC50s (p-value = 1.64 × 10^−4^ and 0.46 ×10^−2^) (Fig 5B) and for docetaxel, when considering AUC scores (p-value = 3.38 ×10^−3^; S5 Fig). The scatter plots (Fig 5B) suggests a consistent association for paclitaxel as well, though the limited sample size (only 2 predicted sensitive cell lines with available IC50 and AUC values) strongly diminish the significance of the test (p-value = 6.1 ×10^−2^). No statistically significant associations to drug response were found between the cell lines negatively connected to the refined signatures when used individually or the paclitaxel optimal signature alone (S6 and S7 Figs.).

Taken together, our results show that through an iterative network-guided connectivity-mapping strategy we can compose gene signatures whose change upon drug treatment precisely reflects the MoA of the considered drugs. Connectivity scores among drugs based on these signatures are effective in predicting an unreported similarity in MoA between glipizide, splitomicin and paclitaxel. Our experimental results show that this similarity is due to the effects exerted by these drugs on the state of microtubules. Additionally, we present a validation on an independent dataset showing the ability of these signatures in predicting drug sensitivity in cancer cell lines whose basal expression is negatively connected to our refined signatures. Finally, the lack of predictive ability of these signatures when used individually to identify cancer cell lines negatively connected to them (and that of the paclitaxel optimal signature, when used alone) further confirms the usefulness of our strategy.

## Conclusions

Signature-matching methods have been exploited in a number of recent works as tools to identify drug-repositioning opportunities and to elucidate a drug’s mode of action (MoA). These methods consider changes in transcription upon drug treatment as the output of a ‘black-box’ mechanism triggered by interaction between the analysed drug and its target. We presented an approach that goes one step further by allowing a supervised refinement of the drug signatures to disentangle them from spurious effects due to drugs’ secondary effects.

In our proof of principle case, we identified microtubule stabilisation associated transcriptional responses, derived by a stepwise refinement in which indirect effects of paclitaxel-induced mitotic arrest and general microtubule disruption are disentangled from microtubule stabilisation. This refinement revealed glipizide and splitomicin as mild microtubule stabilisers, along with several other known microtubule stabilisers. We validated splitomicin and glipizide’s microtubule stabilizing potential in cells using microtubule growth assays. Finally, we showed that sensitivity to microtubule-stabilisers could be predicted using the transcriptional response to microtubule-stabilizers in tumour cells. We found a significant anti-correlation between sensitivity to microtubule stabilising drugs and the level of basal expression of our refined signatures, ascertaining the usefulness of our approach and showing the potential for reasoned use of these signatures in predicting drug sensitivity in cell line models.

Our studies show splitomicin as a microtubule stabiliser promoting congression and segregation defects. This is consistent with chromosomal instability observed in SirT2 deficient animals [44], although this work attributes it to Histone methylation changes through Histone deacetylation defects. Unlike paclitaxel treatment, neither glipizide nor splitomicin cause a steady-increase in mitotic index. Also, splitomicin caused a more severe chromosome congression and segregation defect compared to glipizide. These probably reflect the differences in their extent of stabilising microtubules. Future studies on glipizide’s mode of action will be required to explain the mechanistic reason for this phenotypic difference.

Functional analysis of the refined signatures shows an enrichment of genes associated with sterol synthesis. Whether microtubule stabilisation induces cells to up-regulate genes in these functional classes as a transcriptional feedback has not been tested. Nevertheless, 2-methoxy estradiol, a physiological hormone closely linked to sterol syntheses, is known to possess microtubule pausing properties [45] suggesting the possibility of a transcriptional feedback loop. Additionally, the up-regulation of proteins such as CKAP5, KIF2C and C13of34 (Bora) regulating microtubule dynamics (growth and shrinkage) may be indicative of a negative feedback loop in response to microtubule stabilisation (i.e., the cell responds to microtubule stabilisation by up-regulating proteins required for microtubule dynamics). Similar feedback loops positively regulating the expression of the direct targets of a drug upon treatment have been reported previously [27].

We believe that our approach, readily available as free open-source code, can be broadly applied to other drugs and diseases, as a means to obtain refined signatures of drug response that, as our example shows, can largely improve existing signatures. In our case study we made use of prior knowledge on the MoAs of the cMap compounds from the drug annotations assembled in [13]. This was projected on the drug communities identified in the same study, based on experimental data. However, these annotations could be replaced by any more updated user-defined catalogue, as fully detailed in the documentation of our public available source code. Thus making our approach general and portable.

Ultimately, the refined signatures that can be identified with our approach may also be useful as biomarkers of drug response in the clinic, providing improved predictability of drug resistance and thus allowing stratification of patients. The increasing amount of transcriptional data currently generated, both as drug-induced signatures (http://www.lincscloud.org/) as well as patient’s expression profiles in cancer [46,47] and other context, provide a prospective substrate for such analyses.

## Methods

### Drug network making up and query

Following the guidelines provided in [13], we assembled for each of the 1,309 drugs in the connectivity-map dataset [17] a PRL-signature (Prototype Ranked List). This summarises the transcriptional effect of a given drug across multiple treatments on different cell lines and consists of a genome-wide ranked list of microarray probe-sets, sorted according to their consistent differential expression across these multiple treatments[13].

To compute the similarity between drug A and B, we compared the two corresponding PRL-signatures. To this end, optimal signatures were composed for A and B by selecting the top and bottom 250 probe-sets (the up- and down-regulated genes, respectively) in the corresponding PRL-signatures. This choice is justified as follows. In [13] the authors designed a rank-merging procedure to dilute batch- and cell-line-specific effects on the transcriptional responses to a given drug across multiple experimental settings. While on one hand this method as been shown (in the same study) to improve the classification ability of the GSEA based signature-matching metrics, on the other hand this produces a genome wide PRL for each of the connectivity map compounds. As a consequence no fold-change- or significance-based methods can be applied on the PRLs to derived optimal signatures, suitable to query again the connectivity map. However in [13], the authors heuristically determined (by a simulation study) that picking the top/bottom 250 genes from these PRLs is a good choice. Based on these previously published findings we decided to employ this number of genes. Subsequently, we checked the positions of the genes in the A optimal signature along the B PRL-signature (and vice versa), through Gene Set Enrichment Analysis (GSEA) [29]. For a given gene set, we calculated an enrichment score (ES) by walking down the A PRL-signature, increasing a running-sum statistic (RS) when a gene in the set was encountered and decreasing it when encountering a gene not in the set. Finally, the ES was computed as the maximum deviation from zero observed in this random walk. This quantifies how much the genes in the A optimal signature rank consistently at the top/bottom of the B PRL-signature (and vice versa). The resulting GSEA scores for the two optimal signatures (i.e. A along B PRL-signature and B along A PRL-signature) were finally combined, yielding a single numeric value, which quantifies the distance between compounds A and B.

To build the drug network (DN), we let each node represent a drug PRL-signature and edges connect a node pairs if the distance between the corresponding PRL-signatures falls within the 5% quantile of all the 8.56 × 10^5^ possible drug pair-wise distances.

To functionally characterise the DN we clustered the nodes modules termed ‘communities’. These communities were then annotated by searching for statistically over-represented modes of action (MoAs). To generically compute connectivity scores between a signature of genes and a drug A in the DN, the similarity between the signature *S* = {*U*, *D*} (composed by two sets of genes, U and D, respectively up- and down-regulated) and the PRL-signature of a drug A was quantified. For this, the tendency of the genes in the two sets {*U*, *D*} to group at the extremities of the PRL-signature, was evaluated through a two-tailed GSEA [29]. Thus, the connectivity score (CS) of the A-PRL-signature to the signature S was defined as:

$$CS_{S,A}\;=\;\frac{ES_{A}^{U}-ES_{A}^{D}}{2}.$$

Here, $ES_{A}^{x}$, with (*x* = *U*, *D*), is the Enrichment Score (ES) of the S signature (its up-regulated and down-regulated parte, respectively for U and D, with respect to the A PRL-signature.

$ES_{A}^{x}$ ranges in [-1,1] and it is a measure based on the Kolmogorov-Smirnov statistic. It quantifies the tendency of a set of genes (*x*) to be grouped at the top of a genome wide ranked list of genes (the A PRL-signature) [13,29]. The closer $ES_{A}^{x}$ is to 1, the more the genes in *x* are grouped at the top of the ranked list. The closer -1, the more the genes in *x* are grouped at the bottom.

*CS*_*S*,*A*_ ranges in [-1,1], it is a function of a gene signature *S* = {*U*, *D*} and a ranked list A, and it quantifies the extent to which genes in the U set are placed at the top of A and genes in the D set are placed at the bottom of A. The closer these two statements are to the truth, the closer to 1 is the value of *CS*_*S*,*A*_. The more they are false the more the value of *CS*_*S*,*A*_ is close to -1.

The significance of a given connectivity score *CS*_*S*,*A*_ was defined as deviance from its expectation. To this aim, we generated 10,000 randomised versions of the ranked list A and queried them with the signature S, thus yielding a set of 10,000 empirical values for the CS. From an empirical observation of these randomly generated CSs, we determined that they distribute as a mixture of three Gaussian distributions, therefore a probability density function (pdf) from these empirical values was estimated by fitting a 3-Gaussian mixture model on them. The resulting pdf was then used to compute empirical p-values for the observed CSs. Finally these p-values were corrected for multiple hypotheses testing with the Benjamini-Hochberg method.

To compute connectivity scores for to the basal expression profiles of the GDSC cancer cell lines, we followed the same procedure but in this case the genome wide ranked lists of genes were derived from the basal expression profiles of 1,000 human cancer cell lines, as detailed in the supplementary methods.

### Microtubule growth measurements

HeLa cells expressing EB3-Tdtomato were imaged 48 h after seeding in chambered glass coverslips (Cover glass Lab-tek Chambers, FISHER) in CO2-independent L15 medium (Invitrogen) at 37C. Cells were treated with the drugs for an hour and then imaged using time-lapse microscopy once every 10 seconds for a period of 5 minutes. Z-stacks of 0.1μm thickness were acquired using a 100x NA 1.4 objective on an Applied Precision Deltavision Core microscope equipped with a Xenon 100 W lamp, Cherry-red filter (Chroma), phase-contrast filters and Cascade2 EMCCD camera. The 3-D stacks were analysed using Softworx software.

### Chromosome congression and segregation efficiency and mitotic index

For congression and segregation efficiency, HeLa cells exposed to drug for one hour were treated or untreated with MG132, respectively. Cells were then fixed with methanol and stained with DAPI for DNA as previously described [42]. Congression efficiency was assessed by scoring for unaligned chromosomes in MG132 treated cells with bipolar metaphase spindles. Segregation efficiency was assessed by scoring for mis-segregating chromosomes in MG132 untreated cells with anaphase spindles. DNA analysis was carried out using 100x objective 1.4 N.A on a Deltavision microscope described above. For mitotic index measurements, HeLa cells were seeded in 12 well plates (Thermo Scientific) at a density of 30,000 cells/well and 24 h later exposed to drugs. Six separate areas were chosen to count the percentage of rounded-up mitotic cells using DIC in a Motic AE-31 microscope equipped with a 20x objective.

### Cancer cell lines expression data pre-processing and normalization

We downloaded the basal expression profiles of the panel of cell lines in the Genomics of Drug Sensitivity in Cancer (GDSC) project [21] from ArrayExpress [3] (accession number: E-MTAB-783). From the raw CEL files we computed normalized gene expression intensities by using the Robust Multi-Array Average (RMA) method. Expression values corresponding to multiple probe-sets mapping the same gene were averaged whereas those corresponding to probe-sets mapping to multiple genes were discarded.

Finally we further normalised the resulting expression datasets gene-wisely as follows. We first estimated the probability distribution *P*_*g*_ describing the expression of a given gene g across the cell lines by using a non-parametric Gaussian kernel estimator. Then we assigned to each expression values *x*_*g*,*l*_ (of gene *g* in cell line *l*) an expression-statistic score equal to

$$z_{g,l}\;=\;log\left(\frac{CDF_{g}\left(x_{g,l}\right)}{1-CDF_{g}\left(x_{g,l}\right)}\right).$$

Where *CDF*_*g*_(*x*) is the cumulative distribution of gene *g* at *x*. Finally we generated a genome-wide ranked list of genes (CLR) for each cell line in the panel, by sorting all the genes according to their expression-statistic scores in that cell line, in decreasing order.

## Supporting Information

S1 Fig Paclitaxel/Proteasome-inhibitors running sums and microtubule stabilising running sums. (PDF)

S2 Fig Acetylated tubulin. (PDF)

S3 Fig Chromosome congression and mitotic enrichments. (PDF)

S4 Fig GDSC cell line running sums. (PDF)

S5 Fig AUC scatterplots. (PDF)

S6 Fig Predictive ability comparisons. (PDF)

S7 Fig Predictive ability comparison summary. (PDF)

S1 Table Communities in the first paclitaxel neighbourhood. (XLSX)

S2 Table Paclitaxel optimal signature and GO enrichment analysis. (XLSX)

S3 Table Paclitaxel/Proteasome-inhibitors inconsistent signature and GO analysis. (XLSX)

S4 Table Paclitaxel 1^st^ refined neighbourhood. (XLSX)

S5 Table Microtubule stabilising signature and GO enrichment analysis. (XLSX)

S6 Table GDSC cell line drug sensitivity profiles. (XLSX)

S1 Text (Supplementary Methods—contains also legends for all the supplementary figures and tables). (DOCX)

S2 Text (Supplementary Code) and S1 Dataset–Prototype ranked lists for all the Connectivity Map compounds: All the code and data objects used to produce the results presented in the manuscript are enclosed together with detailed instructions, and also publicly available on GitHub at https://github.com/francescojm/iNRG_cMap and at http://www.ebi.ac.uk/~iorio/PLoS_ONE_Submission. (PDF)
