# Supplementary material for: A Semi-Supervised Approach for Refining Transcriptional Signatures of Drug Response and Repositioning Predictions
Source: PLoS One. 2015 Oct 9;10(10):e0139446. doi: 10.1371/journal.pone.0139446 (PMC4599732; doi:10.1371/journal.pone.0139446)
Supplement: S1 Fig — (PDF) [file pone.0139446.s001.pdf]

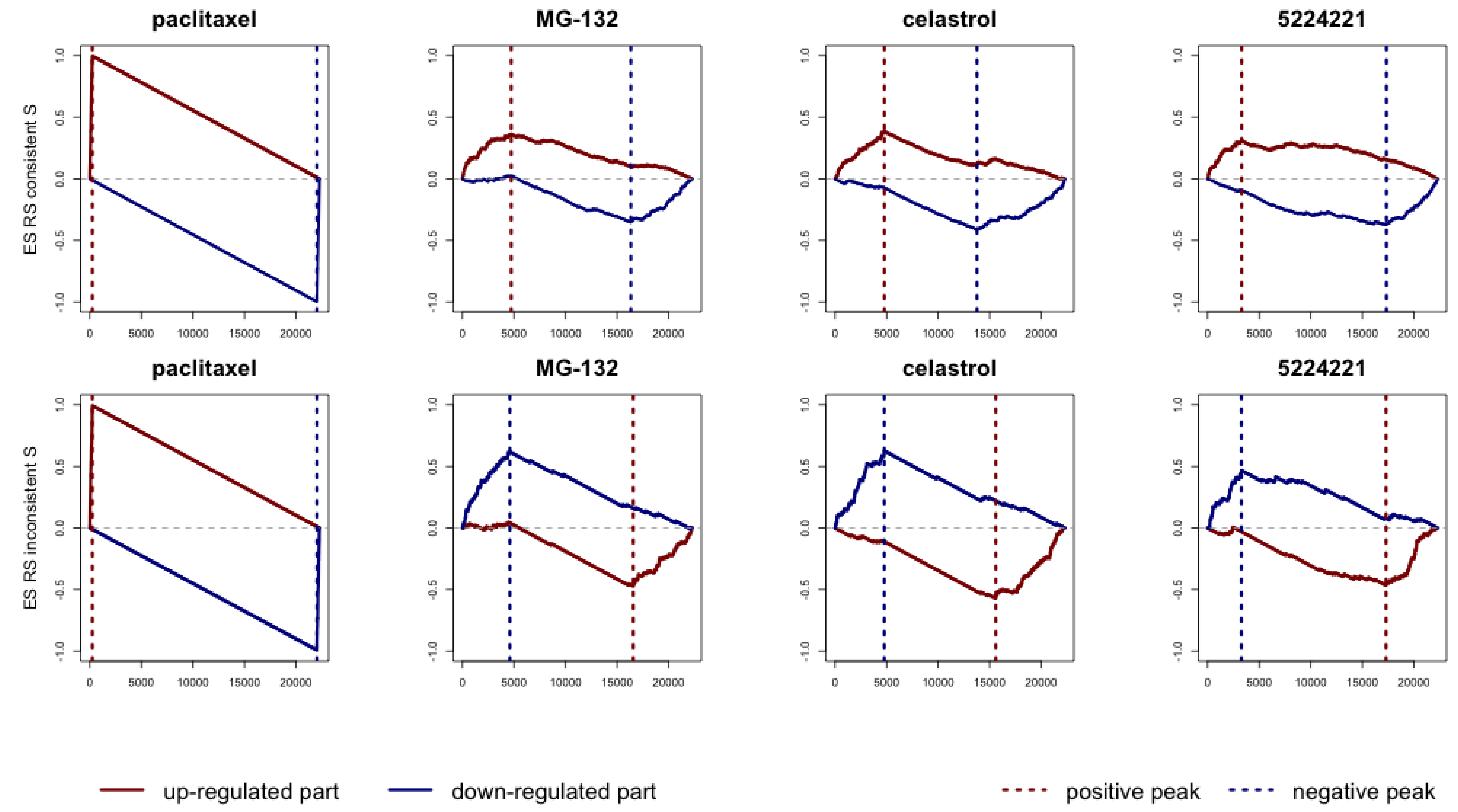

paclitaxel
MG-132
celastrol
5224221
ES RS consistent S
ES RS inconsistent S
1.0
0.5
0.0
-0.5
-1.0
0
5000
10000
15000
20000
up-regulated part
down-regulated part
positive peak
negative peak

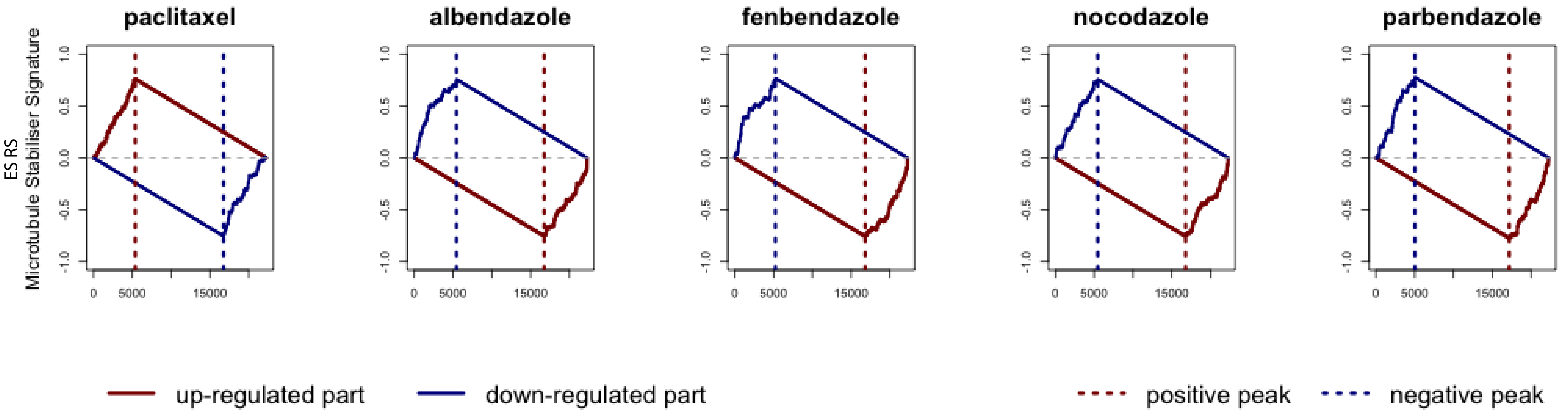

paclitaxel
albendazole
fenbendazole
nocodazole
parbendazole
ES RS
Microtubule Stabiliser Signature
1.0
0.5
0.0
-0.5
-1.0
0
5000
15000
up-regulated part
down-regulated part
positive peak
negative peak
