# Supplementary figures and images for: A Semi-Supervised Approach for Refining Transcriptional Signatures of Drug Response and Repositioning Predictions
Source: PLoS One. 2015 Oct 9;10(10):e0139446. doi: 10.1371/journal.pone.0139446 (PMC4599732; doi:10.1371/journal.pone.0139446)

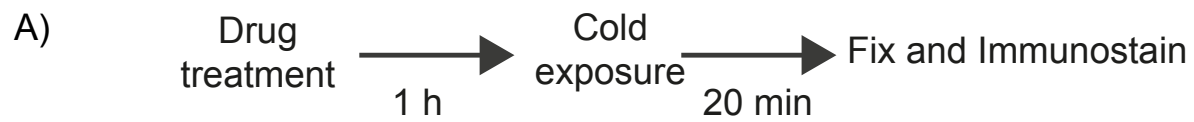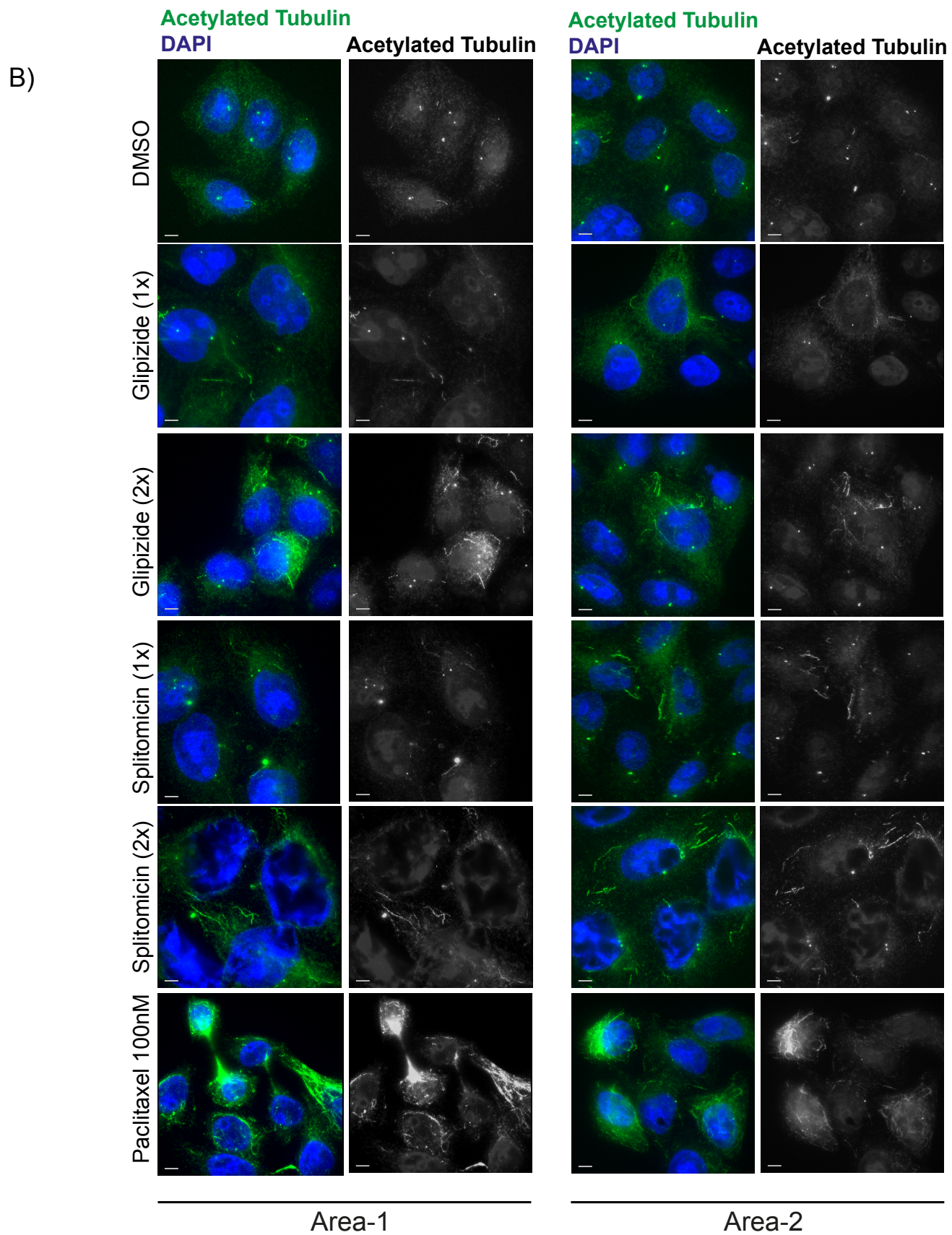

Supplement: S2 Fig — (PDF) [file pone.0139446.s002.pdf]

**A**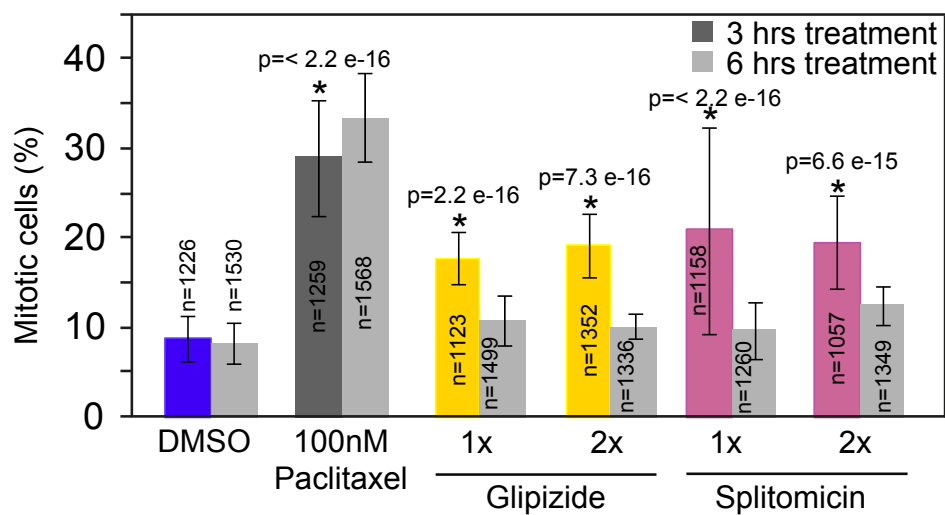**B**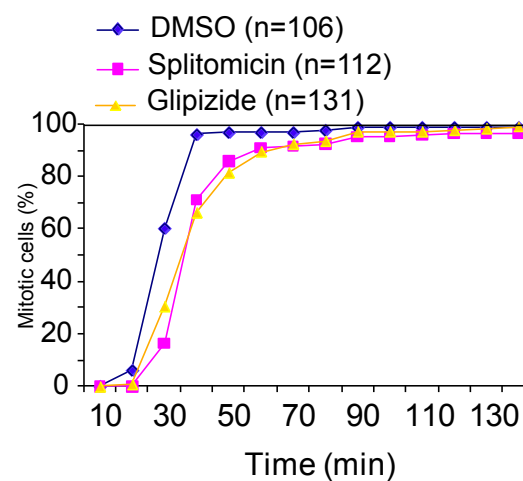**C**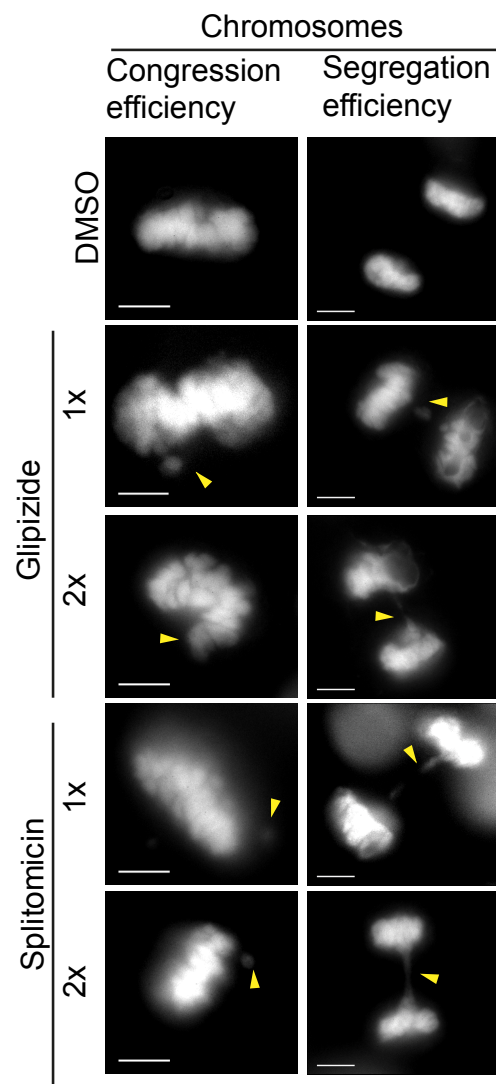**D**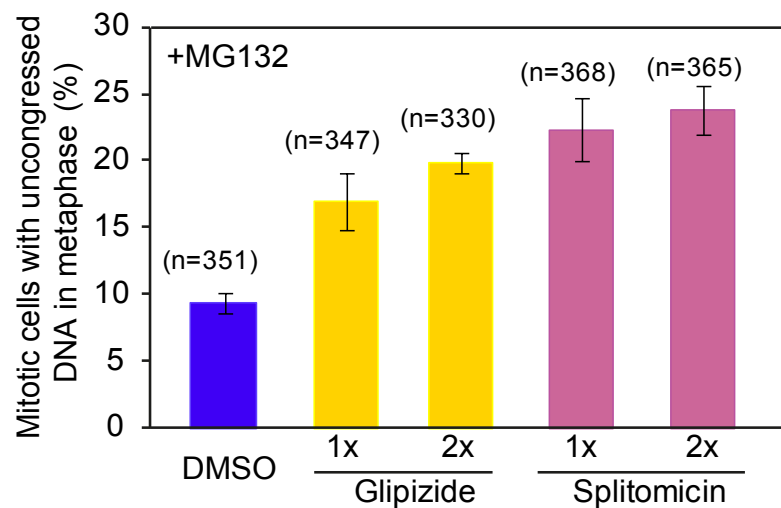**E)**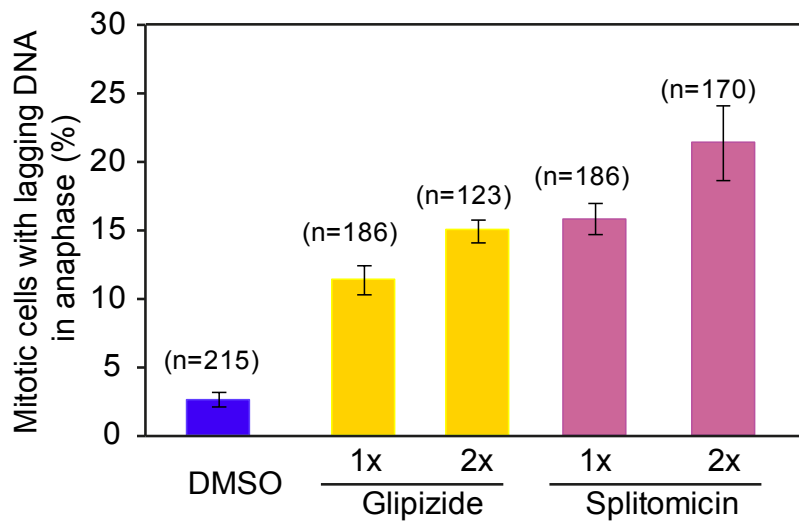

Supplement: S3 Fig — (PDF) [file pone.0139446.s003.pdf]

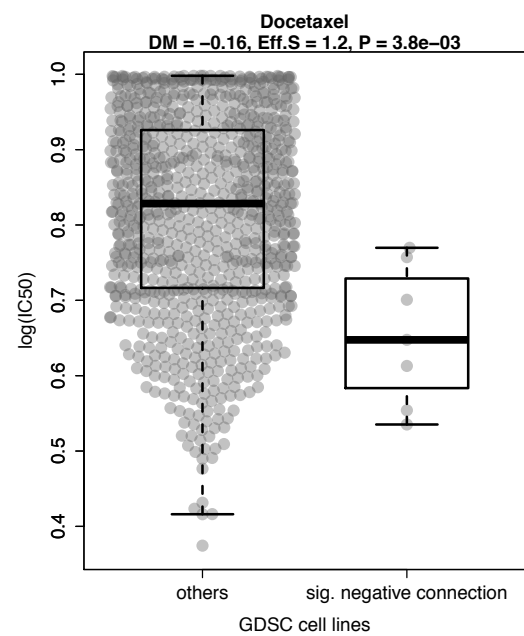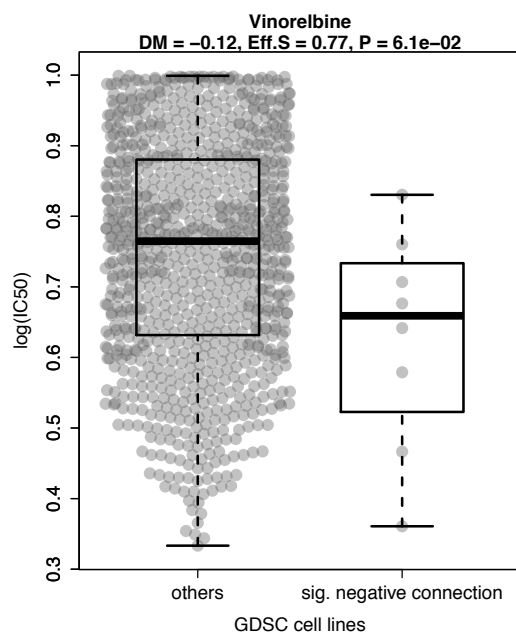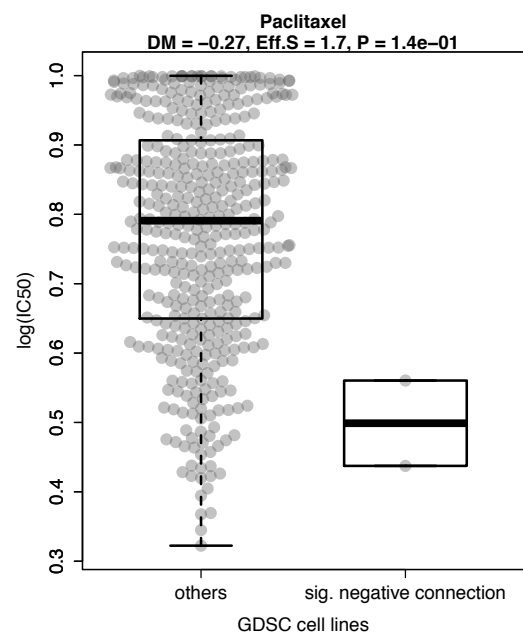

Supplement: S5 Fig — (PDF) [file pone.0139446.s005.pdf]
