# Supplementary material for: A Semi-Supervised Approach for Refining Transcriptional Signatures of Drug Response and Repositioning Predictions
Source: PLoS One. 2015 Oct 9;10(10):e0139446. doi: 10.1371/journal.pone.0139446 (PMC4599732; doi:10.1371/journal.pone.0139446)
Supplement: S4 Fig — (PDF) [file pone.0139446.s004.pdf]

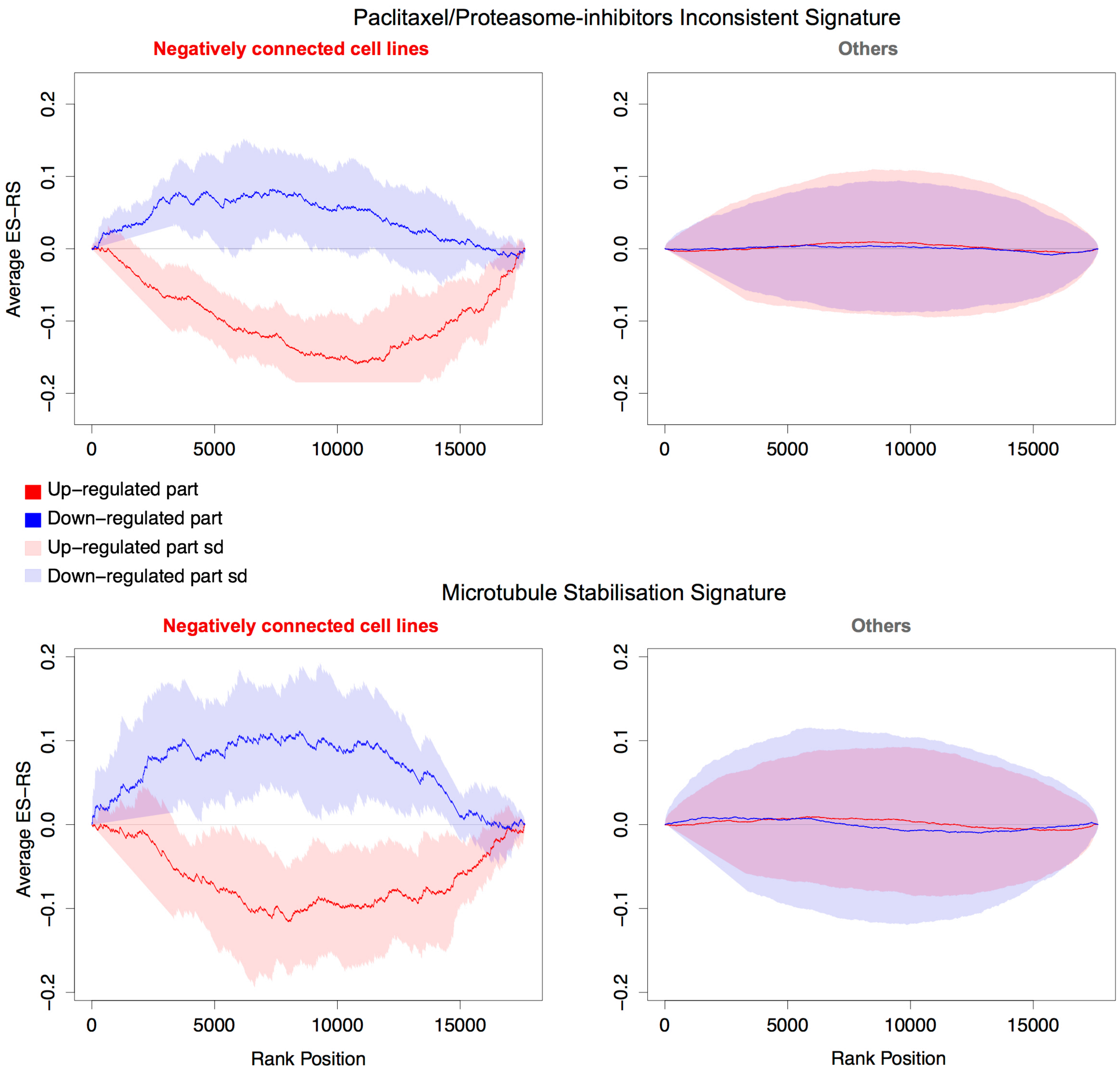
Paclitaxel/Proteasome-inhibitors Inconsistent Signature
Negatively connected cell lines
Others
Average ES−RS
0.2
0.1
0.0
−0.1
−0.2
0
5000
10000
15000
Up−regulated part
Down−regulated part
Up−regulated part sd
Down−regulated part sd
Microtubule Stabilisation Signature
Negatively connected cell lines
Others
Rank Position
Rank Position
