# Supplementary material for: A Semi-Supervised Approach for Refining Transcriptional Signatures of Drug Response and Repositioning Predictions
Source: PLoS One. 2015 Oct 9;10(10):e0139446. doi: 10.1371/journal.pone.0139446 (PMC4599732; doi:10.1371/journal.pone.0139446)
Supplement: S6 Fig — (PDF) [file pone.0139446.s006.pdf]

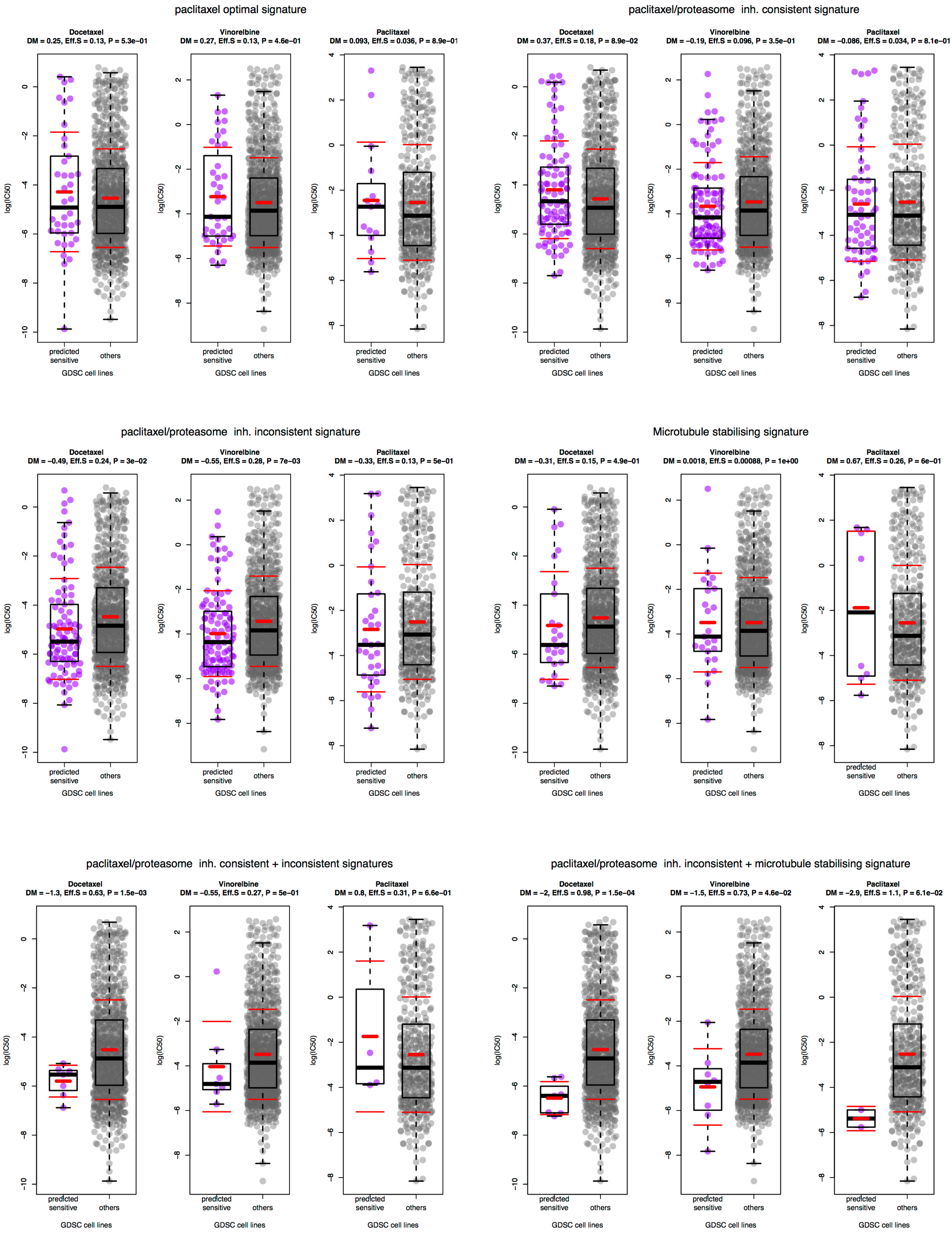
paclitaxel optimal signature
Docetaxel
DM = 0.25, Eff.S = 0.13, P = 5.3e−01
Vinorelbine
DM = 0.27, Eff.S = 0.13, P = 4.6e−01
Paclitaxel
DM = 0.093, Eff.S = 0.036, P = 8.9e−01
paclitaxel/proteasome inh. consistent signature
Docetaxel
DM = 0.37, Eff.S = 0.18, P = 8.9e−02
Vinorelbine
DM = −0.19, Eff.S = 0.096, P = 3.5e−01
Paclitaxel
DM = −0.086, Eff.S = 0.034, P = 8.1e−01
paclitaxel/proteasome inh. inconsistent signature
Docetaxel
DM = −0.49, Eff.S = 0.24, P = 3e−02
Vinorelbine
DM = −0.55, Eff.S = 0.28, P = 7e−03
Paclitaxel
DM = −0.33, Eff.S = 0.13, P = 5e−01
Microtubule stabilising signature
Docetaxel
DM = −0.31, Eff.S = 0.15, P = 4.9e−01
Vinorelbine
DM = 0.0018, Eff.S = 0.00088, P = 1e+00
Paclitaxel
DM = 0.67, Eff.S = 0.26, P = 6e−01
paclitaxel/proteasome inh. consistent + inconsistent signatures
Docetaxel
DM = −1.3, Eff.S = 0.63, P = 1.5e−03
Vinorelbine
DM = −0.55, Eff.S = 0.27, P = 5e−01
Paclitaxel
DM = 0.8, Eff.S = 0.31, P = 6.6e−01
paclitaxel/proteasome inh. inconsistent + microtubule stabilising signature
Docetaxel
DM = −2, Eff.S = 0.98, P = 1.5e−04
Vinorelbine
DM = −1.5, Eff.S = 0.73, P = 4.6e−02
Paclitaxel
DM = −2.9, Eff.S = 1.1, P = 6.1e−02
log(IC50)
predicted sensitive
others
GDSC cell lines
