# Supplementary material for: A Semi-Supervised Approach for Refining Transcriptional Signatures of Drug Response and Repositioning Predictions
Source: PLoS One. 2015 Oct 9;10(10):e0139446. doi: 10.1371/journal.pone.0139446 (PMC4599732; doi:10.1371/journal.pone.0139446)
Supplement: S7 Fig — (PDF) [file pone.0139446.s007.pdf]

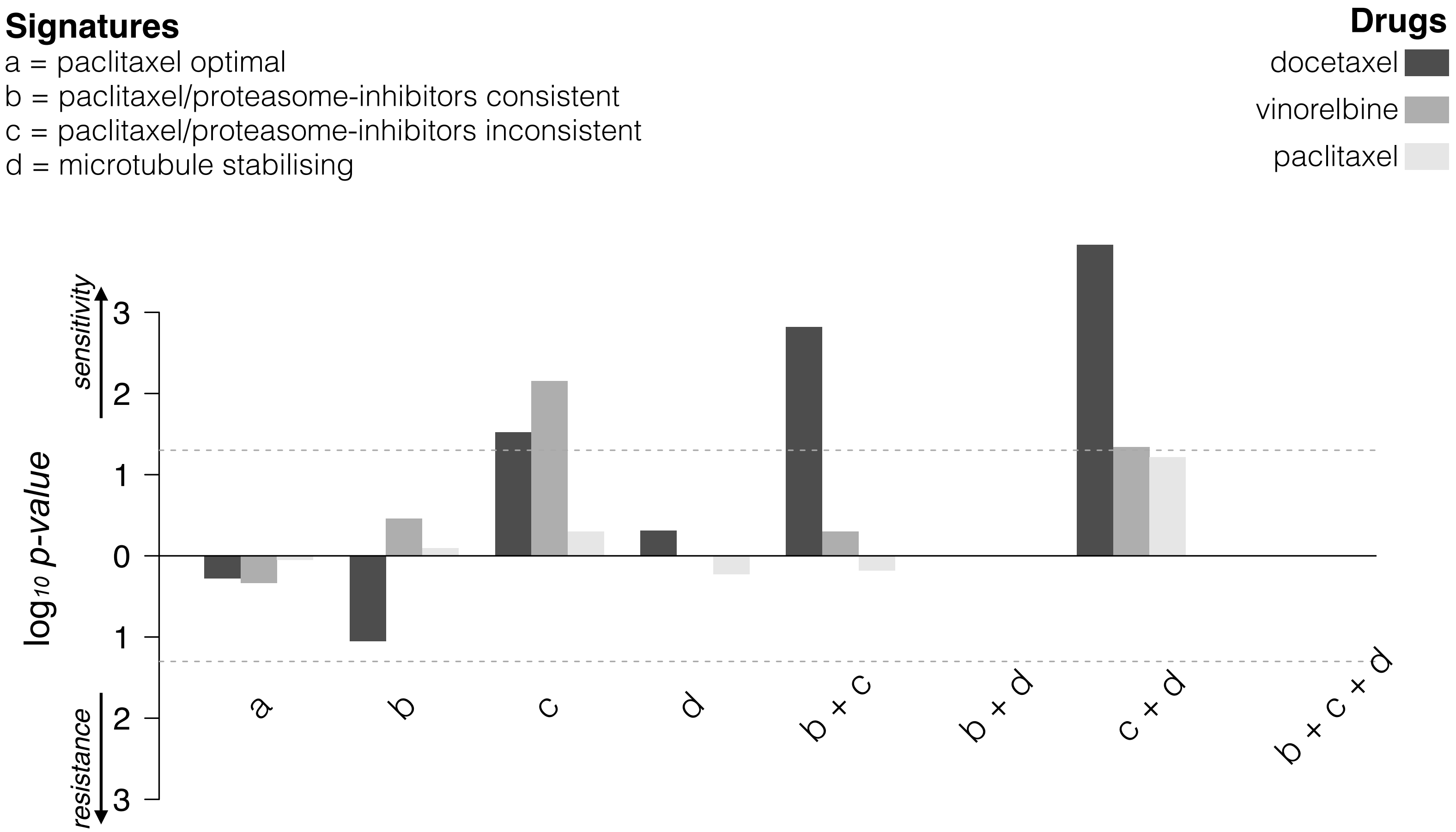
Signatures
a = paclitaxel optimal
b = paclitaxel/proteasome-inhibitors consistent
c = paclitaxel/proteasome-inhibitors inconsistent
d = microtubule stabilising
Drugs
docetaxel
vinorelbine
paclitaxel
sensitivity
resistance
log10 p-value
3
2
1
0
1
2
3
a
b
c
d
b + c
b + d
c + d
b + c + d
