# Supplementary material for: A Semi-Supervised Approach for Refining Transcriptional Signatures of Drug Response and Repositioning Predictions
Source: PLoS One. 2015 Oct 9;10(10):e0139446. doi: 10.1371/journal.pone.0139446 (PMC4599732; doi:10.1371/journal.pone.0139446)
Supplement: S1 Text — (DOCX) [file pone.0139446.s014.docx]

# A semi-supervised approach for refining transcriptional signatures of drug response and repositioning predictions

Francesco Iorio, Roshan L. Shrestha, Nicolas Levin, Viviane Boillot, Mathew Garnett,

Julio Saez-Rodriguez, Viji M. Draviam

# Supplementary materials and methods

Connecting the GDSC cell lines to the refined signatures

In the typical ‘signature-reversion’ analysis pipeline (used in all the aforementioned publications (1,7-10)), gene expression profiles are assembled from publicly available resources or newly generated, to conduct a differential expression analysis between diseased- and normal-phenotype, for a disease of interest. This usually ends up into a set of genes combined with their pattern of expression summarizing the transcriptional differences between the two phenotypes, i.e. a disease-signature. Finally the cMap dataset is queried by means of GSEA (or slightly different methods) to search for drugs whose transcriptional response is anti-correlated to the identified disease-signature. Successful hits are called ‘connections’ to the disease and the involved drugs are tested for their ability in ameliorating the phenotype under consideration (or some of its traits).

Even if moving in the same alley, our aim is different. As starting point we have a drug and a set of cancer cell lines (i.e. different diseases), together with their basal transcriptional profiles (from which signatures can be derived) and their scores of response to the drug. We aim at testing the extent of anti-correlation between the drug signature and the signature of the diseases in which that drug is effective. In our case the drug is paclitaxel, its response signatures are those derived as explained in the previous sections, the disease signatures (in our case different cancer cell lines signatures) are derived from the GDSC dataset together with the paclitaxel sensitivity scores. Additionally, the GDSC transcriptional datasets does not include profiles from microarray hybridizations of mRNA extracted from normal (healthy) tissue. For this reason it is not possible to characterise differentially expressed genes for each cancer cell line with respect to its ‘normal’ counter part. Consequently we normalised the collection of transcriptional profiles as explained in the previous section and we computed expression-level score for each gene in each cell line sample, quantifying how much each gene is ‘highly’ or ‘lowly’ expressed in a given sample considering the distribution of its over-all expression across all the cell lines. Finally, cell line ranked lists of genes (CRL) were computed for each cell line by sorting all the genes according to their expression-level score in decreasing order.

To search for cell lines whose basal CRL was ‘opposite’ (or anti-correlated) to the paclitaxel signatures, we computed connection scores as done when we were aiming at connecting the refined paclitaxel signatures to the cMap drug PRL-signatures. Briefly we checked, using GSEA (3,4) the extent to which genes in the paclitaxel signatures ranked consistently along the CLR of the GDSC cell lines.

#### Gene ontology enrichment analysis and drug-community enrichment analysis

To quantify the statistical over representation of a given community among those associated to drugs in the paclitaxel neighbourhood (or the recurrence of a given GO term among those associated to the genes in one of our signature) we used the same test. In both cases we had to quantify the probability of observing at least *k* objects belonging to a certain category (containing *n* objects in total) within a set of *m* objects randomly extracted from a super-set of *N* total objects. This probability is described by a hyper-geometric distribution and is equal to:

$$PR\left\{ X\geq k \right\}=\sum_{x=k}^{\infty} {\begin{matrix} \left( \begin{matrix} m \\ x \end{matrix} \right) & \left( \begin{matrix} N-m \\ n-x \end{matrix} \right) \end{matrix}}/\left( \begin{matrix} N \\ n \end{matrix} \right).$$

# Supplementary Figure Legends

**Supplementary Figure 1: Plots showing the enrichment score running sums for the paclitaxel/proteasome-inhibitors consistent (respectively inconsistent) signature and the microtubule stabiliser signature**

[top panel] GSEA running sums obtained by querying the ranked lists of paclitaxel, MG-132, celastrol and 5224221 with a signature of genes responding consistently (resp. inconsistently) to the 4 drugs (highlighted in cyan (resp. yellow) in figure 2 (C) of the main).

Red curves refer to genes in up-regulated upon paclitaxel treatment, while blue curves refers to down-regulated ones. The x-axis indicates rank positions along the drug ranked lists (drug-PRL-signature). The x-coordinates of the maximal deviances from zero of the curves along the y-axis (dashed lines) roughly indicate the location where the genes in the considered part of the signature (resp. up- and down-regulated part) tend to accumulate along the ranked list of a given drug. For the plots in the first row, the x-coordinates of these peaks are fairly consistent. This means that the set of genes grouped at the top (resp. bottom) of the paclitaxel ranked list tend to be grouped at the top (resp. bottom) of the ranked lists of the proteasome inhibitors. For the plots in the second row there is an opposite trend: i.e. the set of genes grouped at the top (resp. bottom) of the paclitaxel ranked list tend to be grouped at the bottom (resp. top) of the ranked lists of the proteasome inhibitors.

[bottom panel] as for the top panel but considering the microtubule stabilising signature across the paclitaxel ranked list and those of 4 benzimidazoles.

**Supplementary Figure 2: Cold stable long lived in cells treated with glipizide or splitomicin.**

(A) Schematic of experimental regime to study cold stable long lived microtubules: HeLa cells were treated with different concentrations of Glipizide (20 μM; 1x and 40 μM; 2x) or Spilitomicin (10 μM; 1x and 20 μM; 2x) or 100 nM Taxol for one hour. Cells were then exposed to ice for 20 minutes, followed by fixation with ice cold methanol for 1 min. Fixed cells were blocked with PBST+1% BSA and immunostained with monoclonal Anti-Acetylated Tubulin (1:500) for long lived microtubules. Cells were costained with DAPI, a DNA dye. (B). Images of cells treated as in A, from two separate areas, were analysed to study the presence or absence of cold stable acetylated tubulin following drug treatments as indicated. DMSO (vehicle control) and Taxol were used as negative and positive controls respectively. Scale bar: 5 μm.

**Supplementary Figure 3: Increased incidence of defects in chromosome congression and segregation in cells treated with Glipizide or Splitomicin.**

(A) Graph shows the change in mitotic index in HeLa cells following drug treatments as indicated for either 3 or 6 h. Two different concentrations of Glipizide (20 μM; 1x and 40 μM; 2x) and Splitomicin (10μM; 1x and 20μM; 2x) were used. n represents number of cells from three independent experiments from six different areas. (B) Cumulative frequency graph showing delayed anaphase onset in mitotic cells exposed to 20μM splitomicin or 40 μM glipizide compared to DMSO. Nuclear envelope breakdown to anaphase onset times were measured using time-lapse DIC movies of HeLa cells. ‘n’ refers to number of cells from two independent repeats. (C) Images of HeLa cells treated with two different concentrations of Glipizide (20 μM; 1x and 40 μM; 2x) or Splitomicin (10μM; 1x and 20μM; 2x) for 1 hour either with MG132 for chromosome congression analysis (left panels) or without MG132 for segregation analysis (right panels). Cells were then fixed and stained with DAPI for DNA. Yellow arrows mark DNA that is unaligned on metaphase plate (left) and lagging DNA that failed to segregate properly (right). Scale bar: 5 μm. (D and E) Graphs show percentage of mitotic cells with defective congression (D) or segregation (E) in cultures treated as in B. n represents number of cells analysed. Error bar represents SEM from three independent experiments.

**Supplementary Figure 4: GDSC cell lines enrichment score running sums**

GSEA running sums obtained by querying the normalised basal expression profiles of the GDSC 1,000 cell lines. Top panels were obtained by querying the profiles with the paclitaxel/proteasome-inhibitors inconsistent signature. Bottom panels were obtained by using the microtubule stabilising signature. Plots in the left column show the average running sums for the cell lines negatively connected to the query signatures, together with shaded areas indicating their standard deviations. Plots in the right column show the same results for the rest of the cell lines in the panel.

**Supplementary Figure 5: GDSC cell lines AUC scatter plots**

Scatter plots and statistical scores showing the extent of difference in the area under the dose response curve (AUC) of the GDSC 1,000 cell lines upon treatment with docetaxel, vinorelbine and paclitaxel when dichotomizing the panel into two groups. The second group refered to as ‘sig.negative connection’ is composed of cell line whose basal expression profile is negatively connected to the paclitaxel/proteasome-inhibitors inconsistent signature and the microtubule stabilising signature.

**Supplementary Figure 6: Predictive ability of individual and combined signatures**

Scatter plots and statistical scores showing the extent of difference in the IC50s of the GDSC 1,000 cell lines upon treatment with docetaxel, vinorelbine and paclitaxel when dichotomizing the panel into two groups. The first group refered to as ‘predicted sensitive’ is composed of cell lines whose basal expression profile is negatively connected to the signature(s) under consideration, whereas the second group contains the cell lines in the rest of the panel. The signature combinations for which there are no cell lines simultaneously connected have been omitted.

**Supplementary Figure 7: Comparison of predictive ability of individual and combined signatures**

Different bar colors refer to different drugs. Each group of bars refer to a different signature or combination of signatures as indicated. The value on the y-axis is given by the log10 p-value of the t-test for a given drug and a given signature (or combination of signatures), where the position with respect to the origin is determined by the association type; above and below zero refers to sensitivity and resistance associations, respectively.

# Supplementary Table Legends

**Supplementary Table 1: Drugs and communities surrounding paclitaxel in the drug network**

List of drugs (a) (with community identifiers and statistical enrichment scores) and drug communities (b) surrounding paclitaxel in the drug network.

The complete list of drugs and drug communities in the whole drug network together with the enriched MoAs is publicly available at:

http://www.pnas.org/content/107/33/14621/suppl/DCSupplemental.

**Supplementary Table 2: Paclitaxel optimal signature and GO enrichment analysis**

(a) The full list of microarray probe-sets coding for genes in the signature used to initially query the drug network for the drugs connected to paclitaxel. It also contains their functional annotation and the percentile in which each probe-set falls when sorting all of them according to their differential expression in the consensual response to paclitaxel.

(b) The GO:terms statistically enriched in the gene signature contained in (a). Only GO:terms with an enrichment p‐value < 0.05 after correction for multiple hypothesis testing are reported. For each of them, the average percentile of the associated genes along the genome-wide profile summarizing the consistent response of paclitaxel is provided.

**Supplementary Table 3: Paclitaxel/Proteasome-inhibitors inconsistent signature and GO enrichment analysis**

(a) The up-regulated inconsistent signature containing genes in the up-regulated part of the optimal signature of paclitaxel but falling over the 70% percentile when considering the genome-wide responses of at least two drugs among MG-132, celastrol and 5224221; the Down-regulated inconsistent signature contains genes in the down-regulated part of the paclitaxel optima signature but falling within the 30% percentile when considering the genome-wide response of at least two drugs among MG-132, celastrol and 5224221. In this table the percentile in which each gene falls when considering the genome-wide profile summarizing the responses to the 4 drugs (i.e. paclitaxel, MG-132, celastrol and 5224221) are also provided.

(b) The GO:terms statistically enriched in the gene signature contained in (a). Only GO:terms with an enrichment p‐value < 0.05 after correction for multiple hypothesis testing are reported. For each of them, the average percentiles of the associated genes along the genome-wide profile summarizing the consistent response to the 4 drugs are also reported.

**Supplementary Table 4: Paclitaxel 1^st^ refined neighbourhood**

The connectivity map drugs connected to paclitaxel when querying the resource with the paclitaxel/proteasome-inhibitors consistent and inconsistent signatures both. It contains connectivity and statistical significance scores for both the signatures as well as the final averaged connectivity scores. (a) contains only the drug significantly and positively connected to the signatures whereas (b) contains the scores for all the connectivity map drugs.

**Supplementary Table 5: Microtubule stabilisation signature and GO enrichment analysis**

(a) Genes falling in within the 25% quantile when considering the genome-wide consensual response to paclitaxel but falling over the 70% percentile when considering those of the 4 benzimidazoles and vice-versa; In this table the percentile in which each gene falls when considering the genome-wide profiles summarizing the consensual responses to the 5 drugsare also provided.

(b) The GO:terms statistically enriched in the gene signature contained in (a). Only GO:terms with an enrichment p‐value < 0.05 after correction for multiple hypothesis testing are reported. For each of them, the average percentiles of the associated genes along the genome-wide profile summarizing the consistent response to the 4 drugs are also reported.

**Supplementary Table 6: Connectivity scores of the GDSC 1,000 cell lines to the multiple refined signatures and their sensitivity to Docetaxel, Paclitaxel and Vinorelbine**

Connectivity and correspondent statistical scores for all the cell lines in the GDSC 1,000 panel (versus the multiple signatures) together with their half-maximal inhibitory concentration (IC*50*), and the area under the dose-response curve (AUC) upon treatment with docetaxel, paclitaxel and Vinorelbine.

(a) only cell lines negatively connected to at least 2 signatures. (b) whole bulk of cell lines.

# Supplementary Code and Data

**Supplementary Dataset DS1: Connectivity Map Drugs prototype ranked lists**

Compressed tab delimited txt file containing the ‘prototype ranked lists’ of genes of all the drugs contained in the connectivity map dataset (summarizing their consensual transcriptional response), computed as described in Iorio et al, PNAS 2010.

It can be downloaded at the following URL: http://www.ebi.ac.uk/~iorio/PLoS_CB_Submission.

**All the code and data objects used to produce the results presented in the manuscript are enclosed together with detailed instructions (also available at http://www.ebi.ac.uk/~iorio/PLoS_CB_Submission).**

# References

1. Iorio F, Bosotti R, Scacheri E, Belcastro V, Mithbaokar P, Ferriero R, et al. Discovery of drug mode of action and drug repositioning from transcriptional responses. Proceedings of the National Academy of Sciences. 2010;107:14621.

2. Lamb J. The Connectivity Map: a new tool for biomedical research. Nature Reviews Cancer. 2007;7:54–60.

3. Subramanian A, Tamayo P, Mootha V, Mukherjee S, Ebert B, Gillette M, et al. Gene set enrichment analysis: a knowledge-based approach for interpreting genome-wide expression profiles. Proceedings of the National Academy of Sciences of the United States of America. 2005;102:15545.

4. Frey B, Dueck D. Clustering by passing messages between data points. Science. 2007;315:972.

5. Lamb J, Crawford E, Peck D, Modell J, Blat I, Wrobel M, et al. The Connectivity Map: using gene-expression signatures to connect small molecules, genes, and disease. Science. 2006;313:1929.

6. Iorio F, Tagliaferri R, di Bernardo D. Identifying network of drug mode of action by gene expression profiling. Journal of Computational Biology. 2009;16:241–51.

7. Claerhout S, Lim JY, Choi W, Park Y-Y, Kim K, Kim S-B, et al. Gene expression signature analysis identifies vorinostat as a candidate therapy for gastric cancer. PLoS ONE. 2011;6:e24662.

8. Chen M-H, Yang W-LR, Lin K-T, Liu C-H, Liu Y-W, Huang K-W, et al. Gene Expression-Based Chemical Genomics Identifies Potential Therapeutic Drugs in Hepatocellular Carcinoma. Agoulnik I, editor. PLoS ONE. 2011;6:e27186.

9. Kunkel SD, Suneja M, Ebert SM, Bongers KS, Fox DK, Malmberg SE, et al. mRNA Expression Signatures of Human Skeletal Muscle Atrophy Identify a Natural Compound that Increases Muscle Mass. Cell Metabolism. 2011;13:627–38.

10. Dudley JT, Sirota M, Shenoy M, Pai RK, Roedder S, Chiang AP, et al. Computational Repositioning of the Anticonvulsant Topiramate for Inflammatory Bowel Disease. Science Translational Medicine. 2011;3:96ra76–6.
