# Supplementary material for: A Semi-Supervised Approach for Refining Transcriptional Signatures of Drug Response and Repositioning Predictions
Source: PLoS One. 2015 Oct 9;10(10):e0139446. doi: 10.1371/journal.pone.0139446 (PMC4599732; doi:10.1371/journal.pone.0139446)
Supplement: S2 Text — (PDF) [file pone.0139446.s015.pdf]

A semi-supervised approach for refining transcriptional signatures of drug response and repositioning predictions

Francesco Iorio[1], Roshan L. Shrestha[2], Nicolas Levin[2], Viviane Boillot[2], Mathew Garnett[3], Julio Saez-Rodriguez[1,*], Viji M. Draviam[2,*]

1 European Molecular Biology Laboratory – European Bioinformatics institute; Wellcome Trust Genome Campus, Hinxton – Cambridge (UK)
2 Department of Genetics - University of Cambridge; Downing Street, Cambridge (UK)
3 Cancer genome project – Wellcome Trust Sanger Institute; Wellcome Trust Genome Campus, Cambridge (UK)

SUPPORTING INFORMATION:
SOURCE CODE

Also available at:
https://github.com/francescojm/iNRG_cMap
and
http://www.ebi.ac.uk/~iorio/PLoS_ONE_Submission

Content

- Connectivity mapping predicts new microtubule stabilising agents and drug sensitivity in cancer cell lines: Instructions to reproduce results and figures included in the paper

- Iterative network guided connectivity mapping pipeline

- Signature reversion pipeline

- Code and data object documentation

- Source Code

Iterative Network-Guided Connectivity mapping

This website and the linked web pages contain functions, scripts and data objects used in the software enclosed to the paper entitled *A semi-supervised approach for refining transcriptional signatures of drug response and repositioning predictions*, by Francesco Iorio et al, submitted as research paper to *PLoS ONE*.

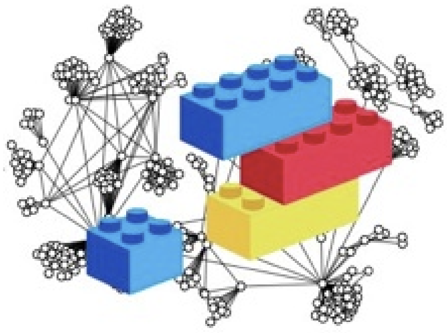

Copyright (c) 2014 - 2019, EMBL - European Bioinformatics Institute
Author: Francesco Iorio (iorio@ebi.ac.uk)
Distributed under the GPLv3 License.
See accompanying file LICENSE.txt or copy at http://www.gnu.org/licenses/gpl-3.0.html Paper website: http://www.ebi.ac.uk/~iorio/PLoS_ONE_Submission

Source code and supplementary data

Supplementary Dataset DS1: cMap Drugs prototype ranked lists
Compressed tab delimited txt file containing the 'prototype ranked lists' of genes for all the drug contained in the connectivity map dataset, computed as described in Iorio et al, PNAS 2010.
SuppDataset_SD1_DRUG_PRLS_txt.zip

How to reproduce results and figures presented in the manuscript?

To start:

Make sure you have R installed. You can download it from http://cran.ma.imperial.ac.uk/

We strongly recommend to install and use the RStudio interface to R, downloadable from: http://www.rstudio.com

Required libraries:
Make sure you have the following libraries installed (all available on the CRAN repository):
- mixtools
- sROC
- pheatmap
- beeswarm

To install them use the following command from the RStudio console:
`install.packages("[library.name]")`
replacing `[library.name]` with each of the library names listed above, in turn.

Working directory creation:

Download and unzip the following compressed folder:
IorioEtAl_R_code_and_objects.zip

Once uncompressed, the content of this folder and its sub-folders should not be changed. Files in the `OUTPUT` subfolder (initially empty) can be moved and/or deleted.

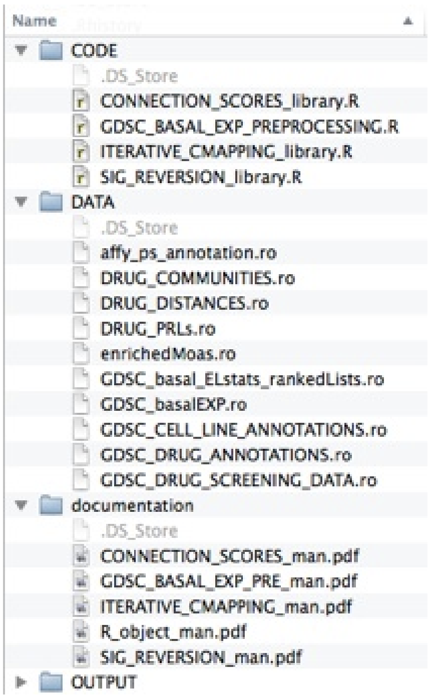

Working directory setup:
To set the working directory to IorioEtAl_R_code_and_objects use the following command from the RStudio console:
`setwd('[path]/IorioEtAl_R_code_and_objects')`
replacing `[path]` with the path of the `IorioEtAl_R_code_and_objects` directory.

Ready to go!

To reproduce results and figure presented in our manuscript execute the commands contained in the following pipelines:

Network guided iterative connectivity mapping pipeline

Pipeline for predictive ability validation through the signature reversion paradigm

Iterative network guided connectivity mapping pipeline

A semi-supervised approach for refining transcriptional signatures of drug response and repositioning predictions

(Supplementary Material and Methods: Supplementary Code)

Francesco Iorio - 24 Aprile 2014

Importing libraries of functions needed to compute connectivity scores and to run the iterative network guided connectivity mapping pipeline:

```
options(warn = -1)
source("CODE/CONNECTION_SCORES_library.R")
```

```
## Loading required package: boot
## Loading required package: MASS
## Loading required package: segmented
## mixtools package, version 1.0.1, Released January 2014
## This package is based upon work supported by the National Science Foundation under Grant No. SES-0518772.
```

```
source("CODE/ITERATIVE_CMAPPING_library.R")
```

Querying the drug network described in Iorio et al (PNAS 2010) using paclitaxel as seed compound:

```
paclitaxelNeighborhood <- DNquery(seed = "paclitaxel", distTh = 0.8065, printToFile = FALSE)
```

Analysing the paclitaxel neighborhood in the drug network (main figure 2 and supplementary table 1):

```
print(paclitaxelNeighborhood[, c("D", "quantile %", "C id", "Adj p-val")])
```

```
##                          D        quantile % C id            Adj p-val
## demecolcine       0.70572 0.449954794261324   48
## 5252917           0.73307  0.82631885114346   48 0.000236989288084179
## pararosaniline    0.75068  1.26062101237493   62
## MG-132            0.75678  1.46398843106884   40
## parbendazole      0.75768  1.49517688643431   90
## celastrol         0.75776  1.49891482865039   40   0.0182249280466375
## 5224221           0.76625  1.84537534780384   40  0.00169840589958832
## splitomicin       0.76715  1.88836168328883   24
## diltiazem         0.77064  2.05528416537591   73
## cytochalasin_B    0.77165  2.10878346334364  100
## fenbendazole      0.77847   2.5058230131085   69
## gefitinib         0.78277  2.79411180652411   60
## suloctidil        0.78504  2.95157262237672   34
## chlortetracycline 0.78788  3.16907413507521   42
## PHA-00665752       0.7887   3.2295820746981   19
## rotenone          0.78983  3.32606770815082   62   0.0694962846867603
## promethazine       0.7901  3.34697682242205   90    0.331175834577628
## ionomycin         0.79515  3.77742423074317   40  0.00251447929397367
## cyproheptadine    0.79774  4.02120814964852   40 0.000400440318057552
## lynestrenol       0.80095  4.35166560368935   40 9.79332311504551e-05
## perhexiline       0.80485   4.7877199253346  100    0.375201411612571
## terfenadine        0.8058  4.90114310945396   34   0.0559034992586342
```

Listing drug communities enriched in the paclitaxel neighborhood (adjusted p-value < 0.05):

```
enriched_cid <- unique(paclitaxelNeighborhood[which(as.numeric(as.character(paclitaxelNeighborhood[,
    "Adj p-val"])) < 0.05), "C id"])
print(enriched_cid)
```

```
## [1] 48 40
## Levels: 100 19 24 34 40 42 48 60 62 69 73 90
```

Listing modes-of-action/Drug-features over-represented in the drug communities enriched in the paclitaxel neighborhood:

```
print(as.character(unique(paclitaxelNeighborhood[which(is.element(paclitaxelNeighborhood[,
    "C id"], enriched_cid)), "MOAs"])))
```

```
## [1] "plant alkaloids // alkaloid"
## [2] "Proteasome inhibitors and UPS modulators // protein synthesis inhibitors (elongation inhibitors) // calcium signal modulators"
```

Deriving paclitaxel/Proteasome-inhibitors consistent/inconsistent signatures (supplementary table 3)

```
P_PI_consistentSig <- DeriveConsistentSignature(seed = "paclitaxel", otherCompounds = c("MG-132",
    "celastrol", "5224221"), PTH = 30, FUZZYNESS = 2, printToFile = FALSE)
P_PI_inconsistentSig <- DeriveInConsistentSignature(seed = "paclitaxel", otherCompounds = c("MG-132",
    "celastrol", "5224221"), PTH = 30, FUZZYNESS = 2, printToFile = FALSE)
```

Visualising a heatmap of expression percentiles of the computed signatures along the prototype ranked lists of paclitaxel, the protasome inhibitors contained in its neighbourhood, and 2 microtubule pertubing drugs included for reference (main figure 3 (A)):

```r
percHeatMaps(c(as.character(P_PI_consistentSig$seedUPreg$ProbeSets), as.character(P_PI_inconsistentSig$seedUPreg$ProbeSets),
    as.character(P_PI_consistentSig$seedDOWNreg$ProbeSets), as.character(P_PI_inconsistentSig$seedDOWNreg$ProbeSets)),
    seed = "paclitaxel", otherCompounds = c("MG-132", "celastrol", "5224221",
        "parbendazole", "chlortetracycline"), printToFile = FALSE)
```

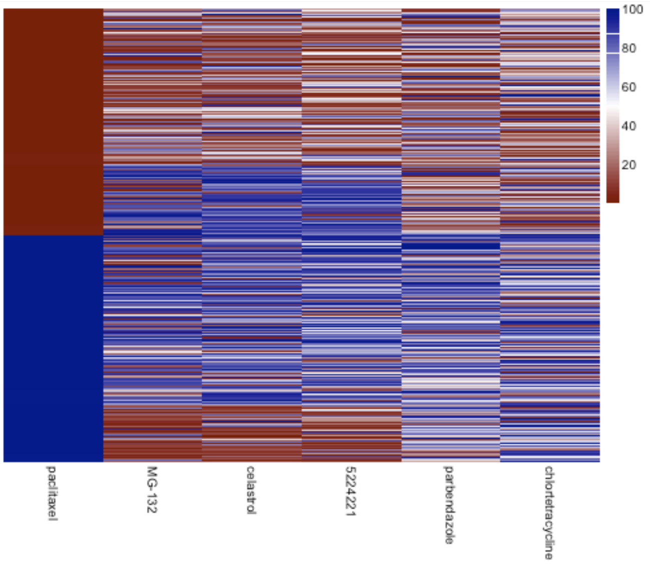

Visualising the enrichment score running sums for of the paclitaxel/proteasome-inhibitors consistent/inconsistent signatures along the prototype ranked lists of paclitaxel and the protasome inhibitors (supplementary figure SF1):

```r
plotRunningSums(P_PI_consistentSig, P_PI_inconsistentSig, seed = "paclitaxel",
    otherCompounds = c("MG-132", "celastrol", "5224221"), printToFile = FALSE)
```

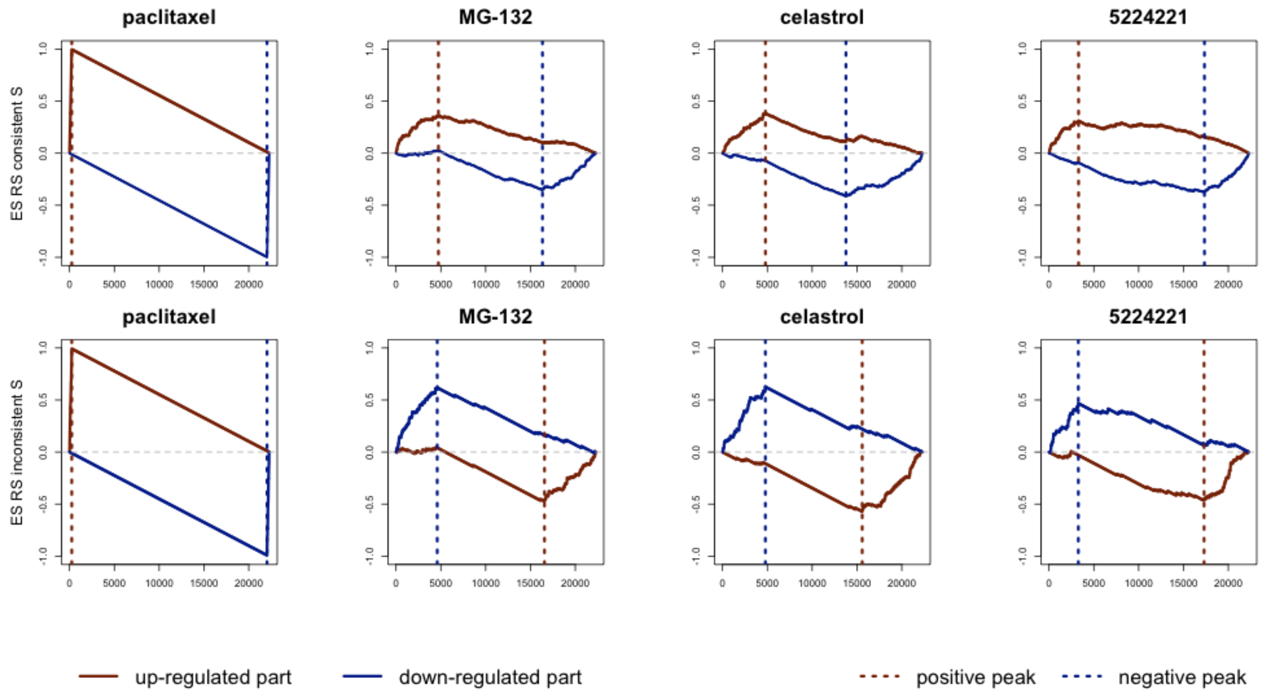

Computing connectivity scores between the prototype ranked lists of all the cMap drugs and the paclitaxel/proteasome-inhibitors consistent/inconsistent signatures (this may take a while):

```r
P_PI_consistent_CS <- CS(P_PI_consistentSig, RANKED_LISTS = DRUG_PRLs, show_progress = FALSE)
```

```
## simulating null model
## number of iterations= 123
## done!
## computing connectivity scores
## Done!
```

```r
P_PI_inconsistent_CS <- CS(P_PI_inconsistentSig, RANKED_LISTS = DRUG_PRLs, show_progress = FALSE)
```

```
## simulating null model
## number of iterations= 26
## done!
## computing connectivity scores
## Done!
```

Combining the obtained connectivity scores to refine the paclitaxel neighbourhood:

```r
first_nb <- combine_2CS(P_PI_consistent_CS, P_PI_inconsistent_CS, printToFile = FALSE,
    fn = "")
```

Visualising paclitaxel 1st refined neighbourhood (main figure 3 (B) and supplementary table 4):

```r
id <- which(first_nb[, "cons S fdr %"] < 5 & first_nb[, "incons S fdr %"] <
    5 & first_nb[, "cons S CS"] > 0 & first_nb[, "incons S CS"] > 0)
print(first_nb[id[2:length(id)], c(4, 8, 9)])
```

```
##                          cons S NCS incons S NCS avg NCS
## 5252917                       4.306        2.719   3.513
## parbendazole                  4.380        1.771   3.076
## splitomicin                   3.657        2.451   3.054
## fenbendazole                  3.916        1.927   2.922
## gefitinib                     3.532        2.228   2.880
## chlortetracycline             3.429        2.226   2.828
## rotenone                      3.850        1.705   2.778
## glipizide                     3.290        2.185   2.738
## albendazole                   3.240        2.115   2.678
## bromocriptine                 3.167        2.142   2.655
## diltiazem                     3.559        1.739   2.649
## cyproheptadine                3.620        1.666   2.643
## moroxydine                    2.614        2.471   2.542
## naloxone                      2.936        2.132   2.534
## perhexiline                   3.425        1.596   2.511
## nilutamide                    2.929        1.886   2.407
## hesperetin                    3.169        1.632   2.400
## nocodazole                    2.627        1.980   2.303
## danazol                       2.584        1.788   2.186
## betulinic_acid                2.712        1.616   2.164
## fluoxetine                    2.722        1.603   2.162
## metolazone                    2.159        2.146   2.153
## hydrastinine                  2.517        1.770   2.144
## practolol                     2.385        1.764   2.074
## genistein                     1.984        2.035   2.010
## monastrol                     2.091        1.779   1.935
## methoxamine                   1.958        1.876   1.917
## primidone                     1.874        1.815   1.844
## 3-hydroxy-DL-kynurenine       1.759        1.921   1.840
## dehydrocholic_acid            1.798        1.854   1.826
## clozapine                     1.933        1.496   1.714
## thioridazine                  1.882        1.533   1.707
## phenazone                     1.739        1.641   1.690
## epiandrosterone               1.744        1.531   1.638
## dihydroergotamine             1.681        1.591   1.636
## chlorphenesin                 1.574        1.531   1.552
```

Deriving a microtubule stabilising signature (supplementary table 5):

```r
MS_sig <- DeriveMSTSignature(seed = "paclitaxel", otherCompounds = c("albendazole",
    "fenbendazole", "nocodazole", "parbendazole"), FUZZYNESS = 4, printToFile = FALSE)
```

Visualising a heatmap of expression percentiles of the microtubule stabilising signature along the prototype ranked lists of paclitaxel and the three benzimidazoles contained in his first refined neighbourhood (figure 3 (C)):

```r
percHeatMaps(c(as.character(MS_sig$seedUPreg$ProbeSets), rev(as.character(MS_sig$seedDOWNreg$ProbeSets))),
    seed = "paclitaxel", otherCompounds = c("albendazole", "fenbendazole", "nocodazole",
        "parbendazole"), printToFile = FALSE)
```

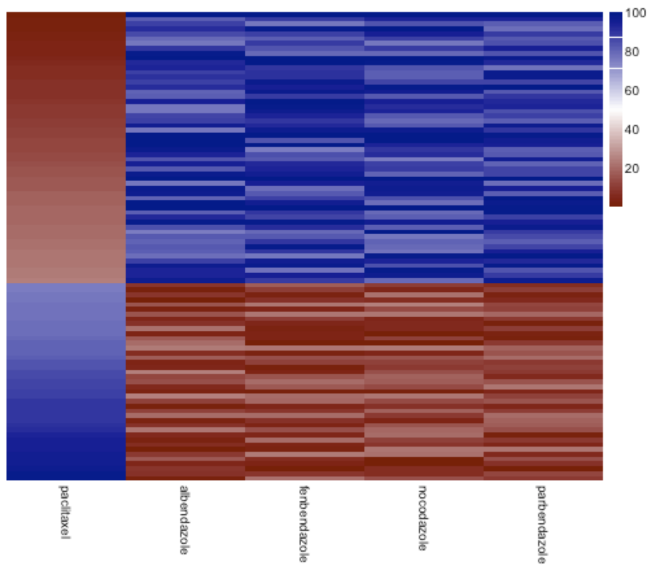

Visualising the enrichment score running sums of the microtubule stabilising signature along the prototype ranked lists of paclitaxel and the recovered benzimidazoles (supplementary figure SF1):

```
plotRunningSumsMST(MS_sig, seed = "paclitaxel", otherCompounds = c("albendazole",
    "fenbendazole", "nocodazole", "parbendazole"), printToFile = FALSE)
```

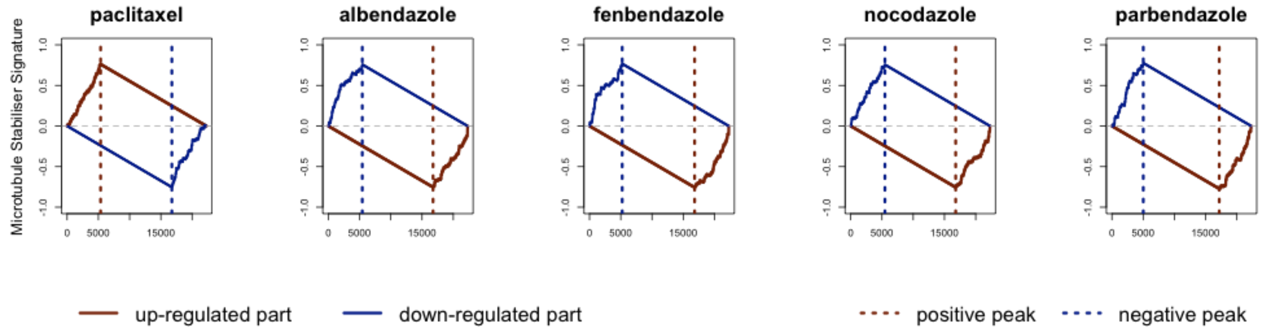

Computing connectivity scores between the prototype ranked lists of all the cMap drugs and the microtubule stabilising signature (this may take a while):

```
MST_CS <- CS(MS_sig, RANKED_LISTS = DRUG_PRLs, show_progress = FALSE)
```

```
## simulating null model
## number of iterations= 34
## done!
## computing connectivity scores
## Done!
```

Combining the obtained connectivity scores to finally refine the paclitaxel neighbourhood:

```
previousConnections <- rownames(first_nb)[which(as.numeric(first_nb[, "avg NCS"]) >
    0 & as.numeric(first_nb[, "cons S NCS"]) > 0 & as.numeric(first_nb[, "incons S NCS"]) >
    0 & as.numeric(first_nb[, "cons S fdr %"]) < 5 & as.numeric(first_nb[, "incons S fdr %"]) <
    5)]

final_nb <- combine_3CS(P_PI_consistent_CS, P_PI_inconsistent_CS, MST_CS, previousNeighBr = previousConnections,
    printToFile = TRUE, fn = "final")
```

Visualising the final refined neighbourhood of paclitaxel (main figure 3 (D), table 1 and supplementary table 4):

```
print(final_nb[2:nrow(final_nb), 5])
```

```
##              glipizide            splitomicin             fluoxetine
##     "2.19989686915533"     "2.04683202514754"     "1.96842993269957"
##             metolazone                5252917              diltiazem
##      "1.8779827209263"     "1.80526353389624"     "1.79016488605669"
##         betulinic_acid             nilutamide             moroxydine
##     "1.77586391018833"     "1.75347275374926"     "1.61659219321536"
##            perhexiline              gefitinib           bromocriptine
##     "1.57596141207419"     "1.54301919561025"     "1.52192398075313"
##               rotenone                danazol       epiandrosterone
```

```
##       "1.4943673200471"      "1.47509920854964"      "1.46695129207344"
##      chlortetracycline            thioridazine          cyproheptadine
##      "1.43213750357412"      "1.38190858522147"      "1.36622967073937"
##           hydrastinine               genistein                naloxone
##      "1.35856721388798"      "1.34265532302282"      "1.31001661205284"
##              monastrol              hesperetin               primidone
##      "1.27449217019113"      "1.26817333429531"      "1.26195005404283"
##              phenazone               clozapine      dehydrocholic_acid
##      "1.21488168491939"      "1.02664929830309"     "0.982238982351692"
##             methoxamine 3-hydroxy-DL-kynurenine                practolol
##     "0.961273540522456"     "0.914417894562068"     "0.885856416275826"
##           chlorphenesin       dihydroergotamine            parbendazole
##     "0.729772935806824"     "0.550035080493067"   "-0.0926467043760503"
##            fenbendazole              albendazole               nocodazole
##    "-0.159860724573494"    "-0.306185924261493"    "-0.555568320480004"
```

Pipeline for predictive ability validation through the signature reversion paradigm

A semi-supervised approach for refining transcriptional signatures of drug response and repositioning predictions

(Supplementary Material and Methods: Supplementary Code)

Francesco Iorio - 24 Aprile 2014

Importing libraries of functions needed to compute connectivity scores and to run the signature reversion pipeline:

```
options(warn = -1)
source("CODE/ITERATIVE_CMAPPING_library.R")
source("CODE/CONNECTION_SCORES_library.R")
```

```
## Loading required package: boot
## Loading required package: MASS
## Loading required package: segmented
## mixtools package, version 1.0.1, Released January 2014
## This package is based upon work supported by the National Science Foundation under Grant No. SES-0518772.
```

```
source("CODE/SIG_REVERSION_library.R")
```

Loading AffyMetrix probe-set annotations:

```
load("DATA/affy_ps_annotation.ro")
```

Loading the GDSC drug screening data and drug annotations for docetaxel, vinorelbine and paclitaxel:

```
load("DATA/GDSC_DRUG_SCREENING_DATA.ro")
load("DATA/GDSC_DRUG_ANNOTATIONS.ro")
```

Loading the annotation file for the GDSC cell lines:

```
load("DATA/GDSC_CELL_LINE_ANNOTATIONS.ro")
```

Load the basal expression statistic ranked lists:

```
load("DATA/GDSC_basal_ELstats_rankedLists.ro")
```

Or, **alternatively**, recomputing them by executing the following commands:

```
source("DATA/GDSC_basal_ELstats_rankedLists.ro")
ELstats <- EL_statistics(basalEXP)
gdsc_basal_ELstats_rankedLists <- basalRanked_lists(ELstats)
```

Generating the gene signatures to be tested individually and in combinations (note that the functions are called with the default parameters):

1. the paclitaxel optimal signature (supplementary table 2)

2. the paclitaxel/protasome-inh. consistent signature

3. the paclitaxel/protasome-inh. inconsistent signature (supplementary table 3)

4. the microtubule stabilising signature (supplementary table 5)

```
paclitaxel_opt_sig <- DeriveSingleSignature()
paclitaxel_PI_con_sig <- DeriveConsistentSignature()
paclitaxel_PI_incon_sig <- DeriveInConsistentSignature()
MI_stab_sig <- DeriveMSTSignature()
```

Converting microarray probe-sets to gene symbols:

```
paclitaxel_opt_sig$seedUPreg$ProbeSets <- affy_ps_annotation[as.character(paclitaxel_opt_sig$seedUPreg$ProbeSets),
    1]
paclitaxel_opt_sig$seedDOWNreg$ProbeSets <- affy_ps_annotation[as.character(paclitaxel_opt_sig$seedDOWNreg$ProbeSets),
    1]
paclitaxel_PI_con_sig$seedUPreg$ProbeSets <- affy_ps_annotation[as.character(paclitaxel_PI_con_sig$seedUPreg$ProbeSets),
    1]
paclitaxel_PI_con_sig$seedDOWNreg$ProbeSets <- affy_ps_annotation[as.character(paclitaxel_PI_con_sig$seedDOWNreg$ProbeSets),
    1]
paclitaxel_PI_incon_sig$seedUPreg$ProbeSets <- affy_ps_annotation[as.character(paclitaxel_PI_incon_sig$seedUPreg$ProbeSets),
    1]
paclitaxel_PI_incon_sig$seedDOWNreg$ProbeSets <- affy_ps_annotation[as.character(paclitaxel_PI_incon_sig$seedDOWNreg$ProbeSets),
    1]
MI_stab_sig$seedUPreg$ProbeSets <- affy_ps_annotation[as.character(MI_stab_sig$seedUPreg$ProbeSets),
    1]
MI_stab_sig$seedDOWNreg$ProbeSets <- affy_ps_annotation[as.character(MI_stab_sig$seedDOWNreg$ProbeSets),
    1]
```

Computing connectivity scores for all the GDSC cell lines and the individual signatures (this may take a while):

```
PACLITAXEL <- CS(paclitaxel_opt_sig, gdsc_basal_ELstats_rankedLists, show_progress = FALSE)
```

```
## simulating null model
## number of iterations= 64
## done!
## computing connectivity scores
## Done!
```

```
P_PI_CON <- CS(paclitaxel_PI_con_sig, gdsc_basal_ELstats_rankedLists, show_progress = FALSE)
```

```
## simulating null model
## number of iterations= 686
## done!
## computing connectivity scores
## Done!
```

```
P_PI_INCON <- CS(paclitaxel_PI_incon_sig, gdsc_basal_ELstats_rankedLists, show_progress = FALSE)
```

```
## simulating null model
## number of iterations= 80
## done!
## computing connectivity scores
## Done!
```

```
MI <- CS(MI_stab_sig, gdsc_basal_ELstats_rankedLists, show_progress = FALSE)
```

```
## simulating null model
## number of iterations= 33
## done!
## computing connectivity scores
## Done!
```

Selecting an fdr threshold and the drugs to be tested:

```
th <- 0.3
DRUGS <- rownames(DRUG_PROPS)
```

Testing the predictive ability of the individual signatures and storing performance scores (supplementary figure SF6) :

```
totRES <- test_pred_ability(list(PACLITAXEL), DRUGS = DRUGS, mainTitle = "paclitaxel optimal signature")
```

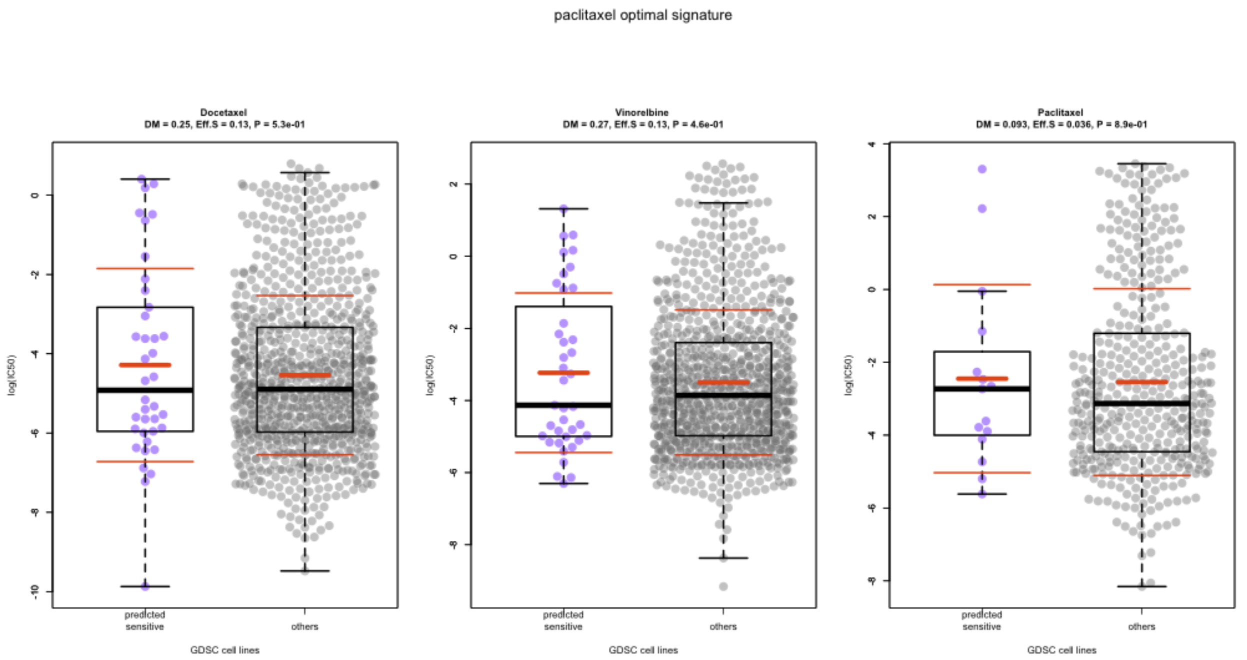


```
totRES <- rbind(totRES, test_pred_ability(list(P_PI_CON), DRUGS = DRUGS, mainTitle = "paclitaxel/proteasome-inh. consistent signature"))
```

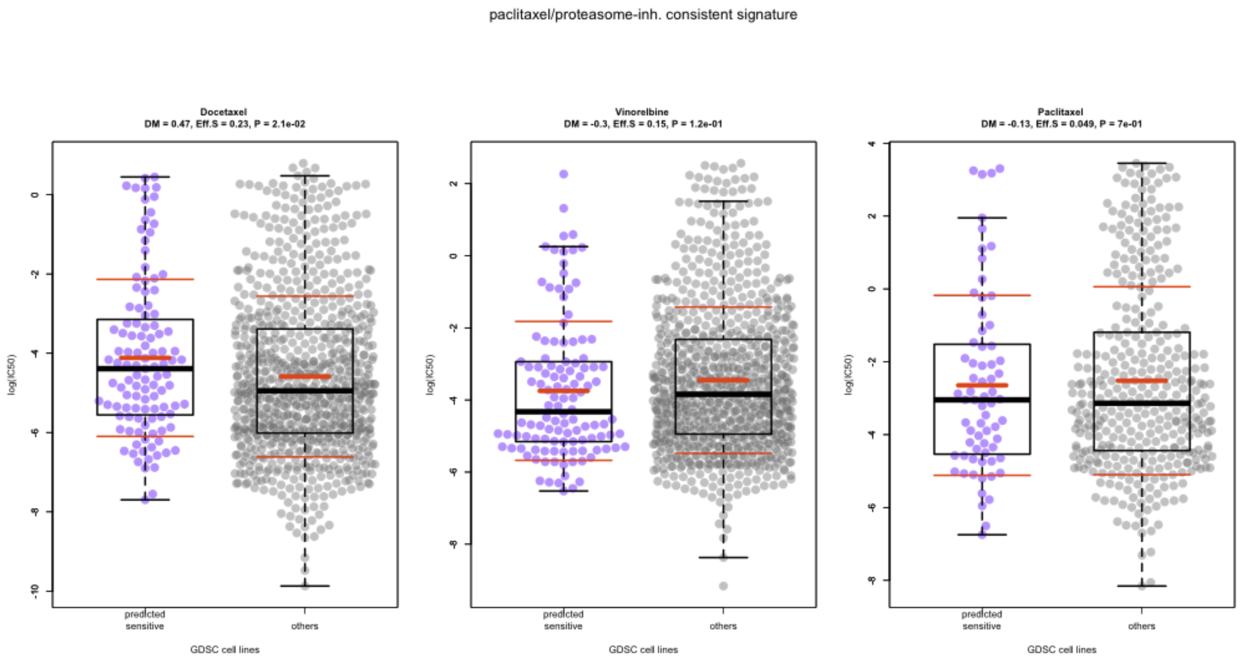


```
totRES <- rbind(totRES, test_pred_ability(list(P_PI_INCON), DRUGS = DRUGS, mainTitle = "paclitaxel/proteasome-inh. inconsistent signature"))
```

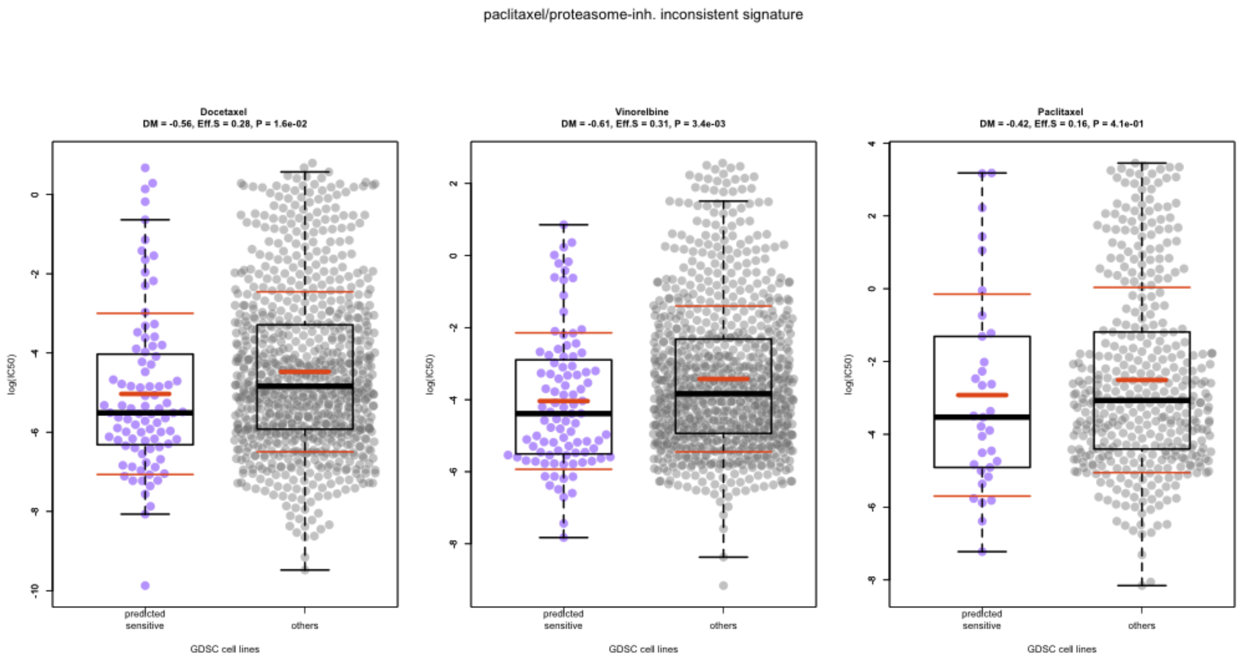


```
totRES <- rbind(totRES, test_pred_ability(list(MI), DRUGS = DRUGS, mainTitle = "Microtubule stabilising signature"))
```

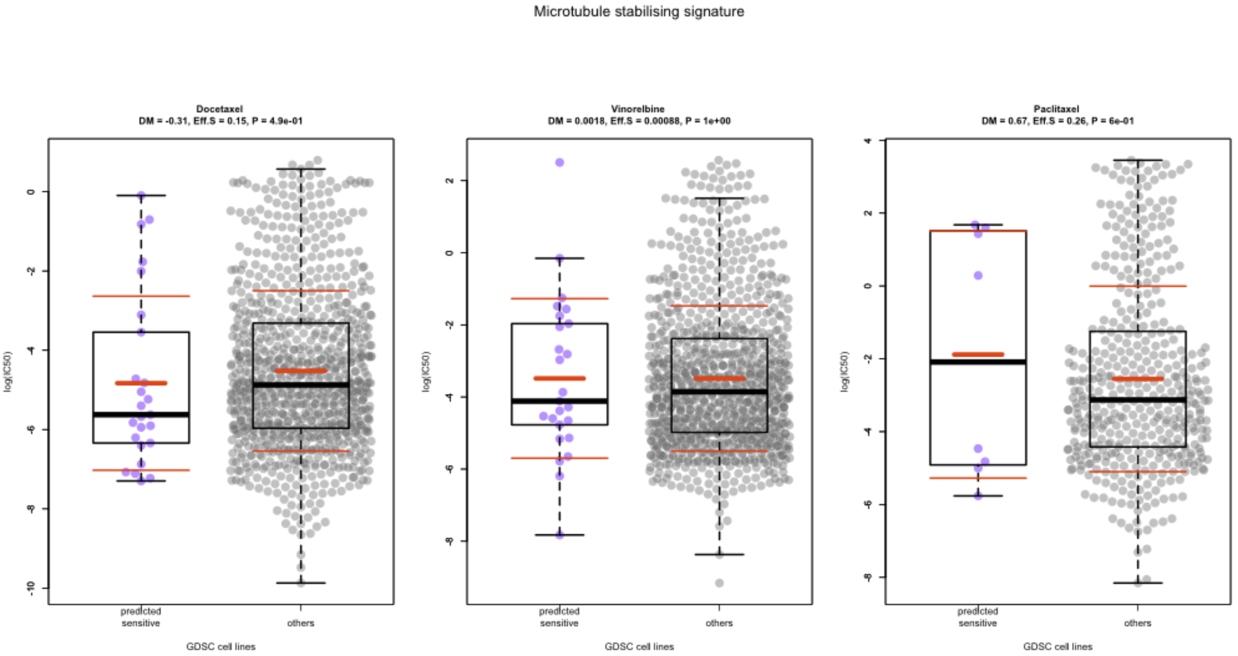

Testing the predictive ability of the combined signatures and storing performance scores (main figure 5 (B) and supplementary figure SF6):

```
totRES <- rbind(totRES, test_pred_ability(list(P_PI_CON, P_PI_INCON), DRUGS = DRUGS,
    mainTitle = "paclitaxel/proteasome-inh. consistent + inconsistent signatures"))
```

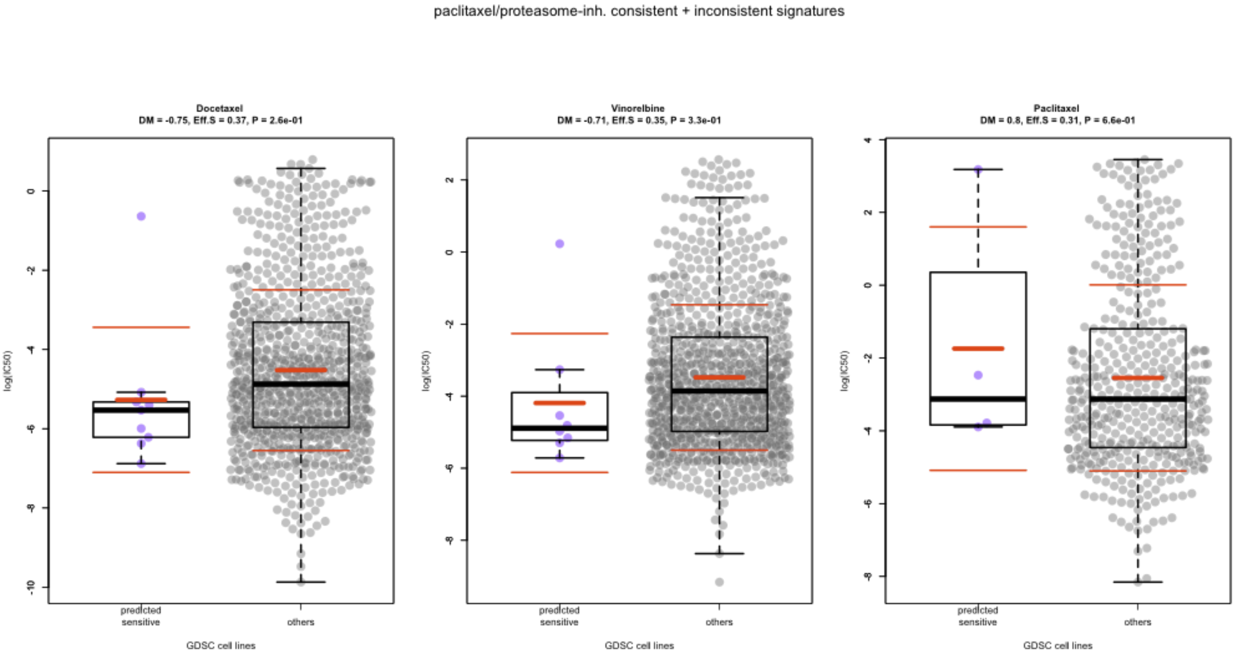


```
totRES <- rbind(totRES, test_pred_ability(list(P_PI_CON, MI), DRUGS = DRUGS,
    mainTitle = "paclitaxel/proteasome-inh. consistent + microtubule stabilising signature"))
```

paclitaxel/proteasome-inh. consistent + microtubule stabilising signature

```
totRES <- rbind(totRES, test_pred_ability(list(P_PI_INCON, MI), DRUGS = DRUGS,
    mainTitle = "paclitaxel/proteasome-inh. inconsistent + microtubule stabilising signature"))
```

paclitaxel/proteasome-inh. inconsistent + microtubule stabilising signature

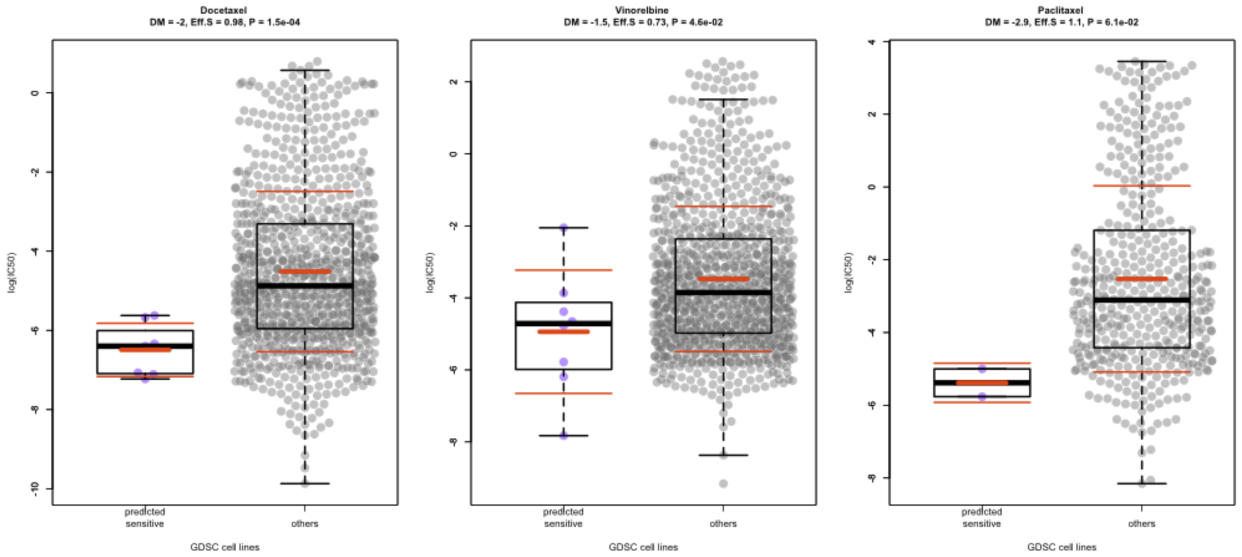


```
totRES <- rbind(totRES, test_pred_ability(list(P_PI_CON, P_PI_INCON, MI), DRUGS = DRUGS,
    mainTitle = "paclitaxel/proteasome-inh. consistent + inconsistent + microtubule stabilising signature"))
```

paclitaxel/proteasome-inh. consistent + inconsistent + microtubule stabilising signature

Summarising the predictive performances for all the tested signatures and signature combinations:

```
performanceMatrix <- matrix(NA, nrow = length(unique(totRES$"used signature(s)")),
    ncol = 3, dimnames = list(unique(totRES$"used signature(s)"), unique(totRES$drug)))
signatures <- unique(totRES$"used signature(s)")



for (i in 1:length(signatures)) {
    idxs <- which(totRES$"used signature(s)" == signatures[i])
    performanceMatrix[i, ] <- log10(as.numeric(as.character(totRES[idxs, "p-val"]))) *
        sign(as.numeric(as.character(totRES[idxs, "deltaMean"])))
}
```

Plotting the performance summary (supplementary figure SF7):

```
par(las = 2)
barplot(t(performanceMatrix), ylab = "association signed p-value", names.arg = c("a",
    "b", "c", "d", "b + c", "b + d", "c + d", "b + c + d"), beside = TRUE, col = c("#000000",
    "#8B8378", "#C1CDCD"), ylim = c(-max(abs(c(performanceMatrix)), na.rm = TRUE),
    max(abs(c(performanceMatrix)), na.rm = TRUE)), border = NA)
abline(h = -log10(0.05), lty = 2, col = "darkgray")
abline(h = log10(0.05), lty = 2, col = "darkgray")
abline(h = 0, lty = 1, col = "black")
legend("topleft", c("docetaxel", "vinorelbine", "paclitaxel"), fill = c("#000000",
    "#8B8378", "#C1CDCD"), border = NA, title = "Drugs")
legend("bottomright", c("a = paclitaxel optimal", "b = paclitaxel/proteasome-inh. consistent",
    "c = paclitaxel/proteasome-inh. inconsistent ", "d = microtubule stabilising"),
    title = "Signatures")
```

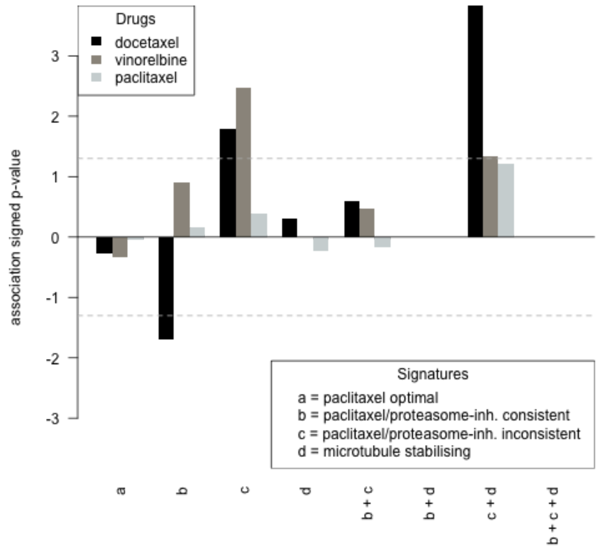
Drugs
docetaxel
vinorelbine
paclitaxel
association signed p-value
3
2
1
0
-1
-2
-3
Signatures
a = paclitaxel optimal
b = paclitaxel/proteasome-inh. consistent
c = paclitaxel/proteasome-inh. inconsistent
d = microtubule stabilising
a
b
c
d
b + c
b + d
c + d
b + c + d

Iterative network guided cMapping and validation

Supplementary Material and Methods - Supplementary Code: R objects documentation

This document describes functions, scripts and data objects used in the software enclosed to the paper entitled *A semi-supervised approach for refining transcriptional signatures of drug response and repositioning predictions*, by Francesco Iorio et al, submitted as research paper to PLoS ONE.

Copyright (c) 2014 – 2019, EMBL - European Bioinformatics Institute
Author: Francesco Iorio (iorio@ebi.ac.uk)
Distributed under the GPLv3 License.
See accompanying file LICENSE.txt or copy at http://www.gnu.org/licenses/gpl-3.0.html
Paper website: http://www.ebi.ac.uk/~iorio/PLoS_ONE_Submission

April 28, 2014

`DRUG_COMMUNITIES` *cMap drug communities*

Description

Data frame containing the community identifiers for 1,233 (out of 1,309) drugs from the Connectivity Map (cMap) dataset [1,2].
These communities have been obtained as described in [3,4].
Row names correspond to drug names. This data frame has been assembled using R and the data in the supplementary materials of [3] publicly available at [5] and [6].

Format

A data frame with 1233 observations on the following 2 variables, specifing for each drug:

`cID` The numerical identifiers of the community

`DRUGS` The drug name

References

[1] Lamb,J. (2007) The Connectivity Map: a new tool for biomedical research. Nature Reviews Cancer, 7, 54-60.

[2] Lamb,J. et al. (2006) The Connectivity Map: using gene-expression signatures to connect small molecules, genes, and disease. Science, 313, 1929.

[3] Iorio,F. et al. (2009) Identifying network of drug mode of action by gene expression profiling. Journal of Computational Biology, 16, 241-251.

[4] Iorio,F. et al. (2010) Discovery of drug mode of action and drug repositioning from transcriptional responses. Proceedings of the National Academy of Sciences, 107, 14621.

[5] http://www.pnas.org/content/107/33/14621.long?tab=ds

[6] http://mantra.tigem.it/About/AboutPnas.aspx.

DRUG_DISTANCES *cMap drug distances*

Description

1,233 x 1,233 double matrix containing the pair-wise distance scores for the 1,233 drugs in the cMap dataset [1,2].
These distances have been computed among drug prototype ranked lists (PRLs) (contained in the DRUG_PRLs object) assembled as described in [3,4] and the supplementary material and methods of our paper.
Row and column names correspond to drug names.
The entry in the i,j position of the matrix contains the distance between the i-th and the j-th drug.

References

[1] Lamb,J. (2007) The Connectivity Map: a new tool for biomedical research. Nature Reviews Cancer, 7, 54-60.

[2] Lamb,J. et al. (2006) The Connectivity Map: using gene-expression signatures to connect small molecules, genes, and disease. Science, 313, 1929.

[3] Iorio,F. et al. (2009) Identifying network of drug mode of action by gene expression profiling. Journal of Computational Biology, 16, 241-251.

[4] Iorio,F. et al. (2010) Discovery of drug mode of action and drug repositioning from transcriptional responses. Proceedings of the National Academy of Sciences, 107, 14621.

DRUG_PRLs *cMap drug prototype ranked lists*

Description

22,283 x 1,309 data frame containing the prototype ranked lists (PRLs) for all the drugs in the cMap dataset [1,2]. For each drug, the PRL consists of a genome-wide list of affyMetrix HG-U133A probe-sets identifiers sorted according to their consensual differential expression upon treatment with the drug under consideration, across a set of human cancer cell lines.

These PRLs have been assembled by post-processing the cMap gene expression profiles through the Kru-Bor method described in [4, 5] and the supplementary material and methods of our paper. Column names of the data frame correspond to drug names.

Format

A data frame with 22283 observations on 1309 variables.

`[drug name]`**a character vector containing 22,283 microrray probe-sets names**

References

[1] Lamb,J. (2007) The Connectivity Map: a new tool for biomedical research. Nature Reviews Cancer, 7, 54-60.

[2] Lamb,J. et al. (2006) The Connectivity Map: using gene-expression signatures to connect small molecules, genes, and disease. Science, 313, 1929.

[3] Iorio,F. et al. (2009) Identifying network of drug mode of action by gene expression profiling. Journal of Computational Biology, 16, 241-251.

[4] Iorio,F. et al. (2010) Discovery of drug mode of action and drug repositioning from transcriptional responses. Proceedings of the National Academy of Sciences, 107, 14621.

`GDSC_CELL_LINE_ANNOTATIONS` *GDSC cell line annotations*

Description

1,471 x 5 data frame, containing the tissue of origin annotations, sample names and COSMIC [1] identifiers for all the cell lines in the GDSC [2] panel.
Row names correspond to COSMIC identifiers.

Format

A data frame with 1471 observations on the following 5 variables, specifying for each cell line:

`Cell.line.name` a character vector containing the cell line name

`Analysis.Set.Name` a character vector containing the cell line names the cell line name

`COSMIC.ID` a character vector containing the COSMIC identifier of the cell lines

`GDSC.description_1` a character vector containing the description of the tissue of origin of the cell line

`GDSC.description_2` a character vector containing the description of the tissue of origin of the cell line (at a different level of specificity)

References

[1] Forbes,S.A. et al. (2011) COSMIC: mining complete cancer genomes in the Catalogue of Somatic Mutations in Cancer. Nucleic Acids Res, 39, D945-50.

[2] Garnett,M.J. et al. (2012) Systematic identification of genomic markers of drug sensitivity in cancer cells. Nature, 483, 570-575.

GDSC_DRUG_ANNOTATIONS *GDSC drug annotations*

Description

3 x 4 data frame, containing the annotations, and target information for docetaxel, vinorelbine, and paclitaxel.
Row names correspond to internal drug identifiers.

Format

A data frame with 3 observations on the following 4 variables, specifying of each drug:

`DRUG_NAME` The name of the drug

`SYNONYMS` Drug name synonyms

`BRAND_NAME` Brand name of the drug

`PUTATIVE_TARGET` Putative targets of the drug

GDSC_DRUG_SCREENING_DATA *GDSC drug screening data*

Description

Data structure containing the GDSC [1] screening data for docetaxel, vinorelbine, and paclitaxel, across the 1,074 human cancer cell lines in the panel.
It contains five 1,074 x 3 double matrix (`IC50s`, `IC90s`, `AUC`, `SLOPE`, and `maxConc`) with cell line COSMIC [2] identifiers as row names and drug internal identifiers as column names.

Format

The entry i,j of each of these contained double matrix, indicates for the treatment of the i-th cell line with the j-th drug:

`IC50s` the half-maximal inhibitory concentration

`IC90s` the 90% inhibitory concentration

`AUC` the normalised area under the dose/response curve

`SLOPE` the maximal concentration tested

References

[1] Garnett,M.J. et al. (2012) Systematic identification of genomic markers of drug sensitivity in cancer cells. Nature, 483, 570-575.

`GDSC_basalRanked_lists` *Ranked lists of genes based on expression level statistics of the GDSC cell lines*

Description

17641 x 715 string matrix, containing genome-wide ranked lists of genes (one for each cell line in the GDSC [1] panel) sorted according to their expression level statistics computed as described in the supplementary material and methods of our paper.
Column names correspond to COSMIC [2] cell line identifiers.

References

[1] Garnett,M.J. et al. (2012) Systematic identification of genomic markers of drug sensitivity in cancer cells. Nature, 483, 570-575.

[2] Forbes,S.A. et al. (2011) COSMIC: mining complete cancer genomes in the Catalogue of Somatic Mutations in Cancer. Nucleic Acids Res, 39, D945-50.

`GDSC_basalEXP` *GDSC cell lines basal expression*

Description

Basal expression profiles of the cell lines in the GDSC [1] panel.

Format

17,641 x 715 double matrix, containing the pre-processed basal expression profiles of the cell lines in the GDSC [1] panel.
Row names correspond to genes and column names correspond to cell line COSMIC [2] identifiers. This matrix has been assembled by downloading the raw gene expression data publicly available at the ArrayExpress repository [3] (accession number: E-MTAB-783) and by pre-processing it as described in the supplementary material and methods of our paper.

References

[1] Garnett,M.J. et al. (2012) Systematic identification of genomic markers of drug sensitivity in cancer cells. Nature, 483, 570-575.

[2] Forbes,S.A. et al. (2011) COSMIC: mining complete cancer genomes in the Catalogue of Somatic Mutations in Cancer. Nucleic Acids Res, 39, D945-50.

[3] Parkinson,H. et al. (2011) ArrayExpress update–an archive of microarray and high-throughput sequencing-based functional genomics experiments. Nucleic Acids Res, 39, D1002-4.

affy_ps_annotation *affyMetrix probe-sets annotation*

Description

Data frame containing the annotation for the Affymetrix probe-sets in the HG-U133A platform, used to in the connectivity map project [1,2]

Format

A data frame with 22277 observations on the following 2 variables.

V1 HUGO symbol(s) for the gene(s) mapped by the probe-sets

V1 annotation(s) of the gene(s) mapped by the probe-sets

Row names correspond to the probe-sets identifiers

References

[1] Lamb,J. (2007) The Connectivity Map: a new tool for biomedical research. Nature Reviews Cancer, 7, 54<e2><80><93>60.

[2] Lamb,J. et al. (2006) The Connectivity Map: using gene-expression signatures to connect small molecules, genes, and disease. Science, 313, 1929.

enrichedMOAs *Modes of action enriched in the drug communities*

Description

String vector containing the modes-of-action or the features enriched in the drug communities described in [1].
Names of the vector correspond to community identifiers. This vector has been assembled by using R and the supplementary material of the publication [1], publicly available at [2].

References

[1] Iorio,F. et al. (2010) Discovery of drug mode of action and drug repositioning from transcriptional responses. Proceedings of the National Academy of Sciences, 107, 14621.

[2] http://www.pnas.org/content/107/33/14621.long?tab=ds

Index

∗Topic **GDSC**
GDSC_basalEXP, 5
GDSC_basalRanked_lists, 5
GDSC_CELL_LINE_ANNOTATIONS, 3
GDSC_DRUG_ANNOTATIONS, 4
GDSC_DRUG_SCREENING_DATA, 4
∗Topic **cMap**
affy_ps_annotation, 6
DRUG_COMMUNITIES, 1
DRUG_DISTANCES, 2
DRUG_PRLs, 2
∗Topic **datasets**
affy_ps_annotation, 6
DRUG_COMMUNITIES, 1
DRUG_DISTANCES, 2
DRUG_PRLs, 2
enrichedMOAs, 6
GDSC_basalEXP, 5
GDSC_basalRanked_lists, 5
GDSC_CELL_LINE_ANNOTATIONS, 3
GDSC_DRUG_ANNOTATIONS, 4
GDSC_DRUG_SCREENING_DATA, 4

affy_ps_annotation, 6

DRUG_COMMUNITIES, 1
DRUG_DISTANCES, 2
DRUG_PRLs, 2

enrichedMOAs, 6

GDSC_basalEXP, 5
GDSC_basalRanked_lists, 5
GDSC_CELL_LINE_ANNOTATIONS, 3
GDSC_DRUG_ANNOTATIONS, 4
GDSC_DRUG_SCREENING_DATA, 4

Iterative network guided cMapping and validation

Supplementary Material and Methods - Supplementary Code: CONNECTION_SCORES

This document describes functions, scripts and data objects used in the software enclosed to the paper entitled *A semi-supervised approach for refining transcriptional signatures of drug response and repositioning predictions*, by Francesco Iorio et al, submitted as research paper to PLoS ONE.

Copyright (c) 2014 – 2019, EMBL - European Bioinformatics Institute
Author: Francesco Iorio (iorio@ebi.ac.uk)
Distributed under the GPLv3 License.
See accompanying file LICENSE.txt or copy at http://www.gnu.org/licenses/gpl-3.0.html
Paper website: http://www.ebi.ac.uk/~iorio/PLoS_ONE_Submission

April 30, 2014

`CS` *Connection scores to multiple ranked lists and statistical significance*

Description

This function computes connections scores of a signature generated with one among the functions

`DeriveSingleSignature`,
`DeriveConsistentSignature`,
`DeriveInconsistentSignature`,
`DeriveMSTSignature`

(all contained in `ITERATIVE_CMAPPING_library.R`) to multiple ranked lists of genes (sorted according to their differential expression, in decreasing order), by computing also statistical significance.
Empirical p-values are computed by simulating a null model through permutation of the ranked lists, by using the `est_emp_Cs` function.

Usage

```
CS(signature, RANKED_LISTS, show_progress = TRUE)
```

Arguments

`signature`	A signature of genes generated as described above
`RANKED_LISTS`	A data frame where each column contains a genome-wide ranked lists of genes or probe-sets compatible with the input signature. This data frame should have more than one column.
`show_progress`	A boolean parameter specifying whether a progress bar should be visualised or not (default = TRUE)

Value

A list of numerical vectors containing for all the columns of `RANKED_LISTS` (i.e. for each inputted ranked list):

`CS`	The obtained connection score

`Pval` The p-value of the obtained connection score

`adjP`

The p-value of the obtained connection score after correction for multiple hypothesys testing

`NCS` The normalised connection score, computed as described in [1]

Author(s)

Francesco Iorio (iorio@ebi.ac.uk)
Copyright (c) 2014 - 2019, EMBL - European Bioinformatics Institute
Distributed under the PLv3 License
See accompanying file LICENSE.txt or copy at http://www.gnu.org/licenses/gpl-3.0.html
Paper website: http://www.ebi.ac.uk/~iorio/PLoS_ONE_Submission

Refer ences

[1] Lamb,J. et al. (2006) The Connectivity Map: using gene-expression signatures to connect small molecules, genes, and disease. Science, 313, 1929. [2] Iorio,F. et al. (2010) Discovery of drug mode of action and drug repositioning from transcriptional responses. Proceedings of the National Academy of Sciences, 107, 14621.

See Also

est_emp_Cs

Examples

```
## loading functions and data objects needed to perform iterative connectivity mapping
source('CODE/ITERATIVE_CMAPPING_library.R')

## generating the optimal signature of digoxin (a cardiac glycoside), as described in [2]
digoxinSig<-DeriveSingleSignature(seed='digoxin')

## querying the prototype ranked lists of digoxin and digoxigenin, digitoxigenin,
## and ouabain (other cardiac glycosides) with the optimal signature of digoxin
CS(digoxinSig,DRUG_PRLs[,c('digoxin','digoxigenin','digitoxigenin','ouabain')])
```

`cMap_CS` *Connection scores computation*

Description

This function computes connections scores of a genome-wide ranked lists of genes (sorted according to their differential expression, in decreasing order) and a signature composed by two sets of genes (up-regulated and down-regulated respoctively), as described in [1,2], by means of unweighted GSEA [3]

Usage

```
cMap_CS(ranked_list, opsig1, returnRS = FALSE)
```

Arguments

`ranked_list`	A string vector containing a genome-wide ranked list of genes sorting according to their differential expression, in decreasing order
`opsig1`	A list composed by two string vectors (`UP` and `DOWN`) containing the up-regulated (resp. down-regulated) genes of the signature
`returnRS`	A boolean parameter specifying if the individual enrichment scores (for the two parts of the signatures), together with the two corresponding obtained running sums should be returned or not (default = FALSE)

Value

The obtained connection score or (if `returnRS == TRUE`) a structure containing the following objects:

`TES`	The obtained connection score
`ESUP`	The enrichment score of the up regulated part of the input signature (i.e. `opsig1$UP`)
`ESDOWN`	The enrichment score of the up-regulated part of the input signature (i.e. `opsig1$DOWN`)
`RSUP`	A numerical vector with the obtained running sum for the up-regulated part of the input signature (i.e. `opsig1$UP`)
`RDOWN`	A numerical vector with the obtained running sum for the up-regulated part of the input signature (i.e. `opsig1$DOWN`)

Author(s)

Francesco Iorio (iorio@ebi.ac.uk)
Copyright (c) 2014 - 2019, EMBL - European Bioinformatics Institute
Distributed under the PLv3 License
See accompanying file LICENSE.txt or copy at http://www.gnu.org/licenses/gpl-3.0.html
Paper website: http://www.ebi.ac.uk/~iorio/PLoS_ONE_Submission

References

[1] Lamb,J. (2007) The Connectivity Map: a new tool for biomedical research. Nature Reviews Cancer, 7, 54-60.

[2] Lamb,J. et al. (2006) The Connectivity Map: using gene-expression signatures to connect small molecules, genes, and disease. Science, 313, 1929.

[3] Subramanian,A. et al. (2005) Gene set enrichment analysis: a knowledge-based approach for interpreting genome-wide expression profiles. Proceedings of the National Academy of Sciences of the United States of America, 102, 15545.

Examples

```
## loading the prototype ranked lists for all the drugs in the connectivity map[1,2] dataset
load('DATA/DRUG_PRLs.ro')

## selecting the PRL of metformin
rankedList<-DRUG_PRLs[,'metformin']

## generating a random signature
signature<-list(UP=DRUG_PRLs[sample(1:5000,250),1],DOWN=DRUG_PRLs[sample(17000:22000,250),1])
```

```
## computing the connection score of the ranked list to the signature
cMap_CS(rankedList,signature)
```

---

combine_2CS — *Combining connection score sets obtained with two different signatures*

Description

This function combines the connection score sets obtained by using two different signatures by qurying with the a set of genome-wide ranked lists of genes, through the function `CS`

Usage

```
combine_2CS(CS1, CS2, printToFile = FALSE, fn = "")
```

Arguments

`CS1`	A list of numerical vectors outputted by the `CS` function when using the first signature as input
`CS2`	A list of numerical vectors outputted by the `CS` function when using the second signature as input
`printToFile`	A boolean parameter specifying if the output of this function should be stored in a tab delimited txt file (default = FALSE). If TRUE then a file, whose name is speficied in the `fn` parameter is created in the `~/OUTPUT` directory (where ~ is the working directory)
`fn`	A string containing the file storing the results. This parameter is ignored if `printToFile = FALSE`

Details

For usage xamples see the pipeline described at
http: //www.ebi.ac.uk/~iorio/PLoS_ONE_Submission iterativeCmappingPL/IterativeCmappingPipeline.html

Value

A data frame containing a row for each queryied ranked list of genes (corresponding to column names). With the following columns:

`cons S CS`	Connection scores obtained with the first signature
`cons S pvalue`	Empirical p-values of connection scores obtained with the first signature
`cons S fdr`	False discovery rate for connection scores obtained with the first signature
`incons S NCS`	Normalised connection scores obtained with the first signature
`incons S CS`	Connection scores obtained with the second signature
`incons S pvalue`	Empirical p-values of connection scores obtained with the second signature
`incons S fdr`	False discovery rate for connection scores obtained with the second signature
`incons S NCS`	Normalised connection scores obtained with the second signature
`avg NCS`	Normlised connection scores averaged across the two signatures

Author(s)

Francesco Iorio (iorio@ebi.ac.uk)
Copyright (c) 2014 - 2019, EMBL - European Bioinformatics Institute
Distributed under the PLv3 License
See accompanying file LICENSE.txt or copy at http://www.gnu.org/licenses/gpl-3.0.html
Paper website: http://www.ebi.ac.uk/~iorio/PLoS_ONE_Submission

---

combine_3CS — *Combining connection score sets obtained with three different signatures on a user defined sub-sets of ranke lists*

Description

This function combines the connection score sets obtained by using two different signatures by qurying with the a set of genome-wide ranked lists of genes, through the function `CS`

Usage

```
combine_3CS(CS1, CS2, CS3, previousNeighBr = "", printToFile = FALSE, fn = "")
```

Arguments

`CS1`	A list of numerical vectors outputted by the `CS` function when using the first signature as input
`CS2`	A list of numerical vectors outputted by the `CS` function when using the second signature as input
`CS3`	A list of numerical vectors outputted by the `CS` function when using the third signature as input
`previousNeighBr`	A string list containing the names of the ranked lists the analysis should focus on
`printToFile`	A boolean parameter specifying if the output of this function should be stored in a tab delimited txt file (default = FALSE). If TRUE then a file, whose name is speficied in the `fn` parameter is created in the `~/OUTPUT` directory (where ~ is the working directory)
`fn`	A string containing the file storing the results. This parameter is ignored if `printToFile = FALSE`

Details

For usage examples see the pipeline described at
http: //www.ebi.ac.uk/~iorio/PLoS_ONE_Submission iterativeCmappingPL/
IterativeCmappingPipeline.html

Value

A data frame containing a row for each queryied ranked list of genes (corresponding to column names). With the following columns:

`P/PI cons S NCS`
: Normalised connection scores obtained with the first signature

`P/PI incons S NCS`
: Normalised connection scores obtained with the second signature

`MST S NCS`
: Normalised connection scores obtained with the third signature

`avg NCS`
: Normlised connection scores averaged across the three signatures

Author(s)

Francesco Iorio (iorio@ebi.ac.uk)
Copyright (c) 2014 - 2019, EMBL - European Bioinformatics Institute
Distributed under the PLv3 License
See accompanying file LICENSE.txt or copy at http://www.gnu.org/licenses/gpl-3.0.html
Paper website: http://www.ebi.ac.uk/~iorio/PLoS_ONE_Submission

---

`est_emp_Cs` *Connection score null model simulation by ranked list permutation*

Description

This function estimates an empirical null distribution of connection scores for a signature of a given size and a set of genome-wide ranked lists of genes. Given the tri-modal nature of the modeled distribution [1], this function returns a 3-gaussian mixture distribution that can be used to estimate connection scores p-values

Usage

```
est_emp_Cs(signature, nt, RANKED_LISTS, show_progress = TRUE)
```

Arguments

`signature`	A list composed by two string vectors (`UP` and `DOWN`) containing the up-regulated (resp. down-regulated) genes of the signature
`nt`	An integer specifying the number of permutations of the ranked lists to be performed
`RANKED_LISTS`	A data frame where each column contains a genome-wide ranked lists of genes or probe-sets compatible with the input signature. This data frame should have more than one column.
`show_progress`	A boolean parameter specifying whether a progress bar should be visualised or not (default = TRUE)

Value

A list of class mixEM

Author(s)

Francesco Iorio (iorio@ebi.ac.uk)
Copyright (c) 2014 - 2019, EMBL - European Bioinformatics Institute
Distributed under the PLv3 License
See accompanying file LICENSE.txt or copy at http://www.gnu.org/licenses/gpl-3.0.html
Paper website: http://www.ebi.ac.uk/~iorio/PLoS_ONE_Submission

Refer ences

[1] Lamb,J. et al. (2006) The Connectivity Map: using gene-expression signatures to connect small molecules, genes, and disease. Science, 313, 1929.

[2] Iorio,F. et al. (2010) Discovery of drug mode of action and drug repositioning from transcriptional responses. Proceedings of the National Academy of Sciences, 107, 14621.

Examples

```
## loading prototype ranked lists for the connectivity map [1] drugs
load('DATA/DRUG_PRLs.ro')

## loading functions and data objects needed to perform iterative connectivity mapping
source('CODE/ITERATIVE_CMAPPING_library.R')

## generating optimal signature for tamoxifen [2]
tamoxifenSig<-DeriveSingleSignature(seed='tamoxifen')

## converting signature format
tamoxifenSig<-list(UP=as.character(tamoxifenSig$seedUPreg$ProbeSets),
                   DOWN=as.character(tamoxifenSig$seedDOWNreg$ProbeSets))

## estimating connection scores null distribution for the tamoxifen siganture
## by executing 10000 permutation of the drug prototype ranked lists
tamoxifenNull<-est_emp_Cs(tamoxifenSig,nt=10000,DRUG_PRLs)

## visualising an histogram with the simulated connection scores
hist(tamoxifenNull$x,100)

## visualising the parameters of the 3-guassian distributions in the mixture model
summary(tamoxifenNull)
```

---

getDrugName *Drug name from internal identifiers*

Description

This function returns the name of the drug whose internal identifier is given in input

Usage

```
getDrugName(id)
```

Arguments

`id`	A string specifying the internal identifier of the drug under consideration

Value

A string specifying the name of the drug

Author(s)

Francesco Iorio (iorio@ebi.ac.uk)
Copyright (c) 2014 - 2019, EMBL - European Bioinformatics Institute
Distributed under the PLv3 License
See accompanying file LICENSE.txt or copy at http://www.gnu.org/licenses/gpl-3.0.html
Paper website: http://www.ebi.ac.uk/~iorio/PLoS_ONE_Submission

---

`getDrugTarget` *Drug target from internal identifiers*

Description

This function returns the target of the drug whose internal identifier is given in input

Usage

```
getDrugTarget(id)
```

Arguments

`id`	A string specifying the internal identifier of the drug under consideration

Value

A string specifying the target of the drug

Author(s)

Francesco Iorio (iorio@ebi.ac.uk)
Copyright (c) 2014 - 2019, EMBL - European Bioinformatics Institute
Distributed under the PLv3 License
See accompanying file LICENSE.txt or copy at http://www.gnu.org/licenses/gpl-3.0.html
Paper website: http://www.ebi.ac.uk/~iorio/PLoS_ONE_Submission

`pnormmix` *Connection scores empirical p-value computation*

Description

This fuction computes the empirical p-value of a connection score, given an empirical null distribution described as a 3-gaussian mixture model (generated by `est_emp_Cs`)

Usage

```
pnormmix(x, mixture)
```

Arguments

`x`	The connection score whose significance should be evaluated
`mixture`	A list of class mixEM generated by `est_emp_Cs` by giving in input the same signature and ranked list used to generate `x`

Author(s)

Francesco Iorio (iorio@ebi.ac.uk)
Copyright (c) 2014 - 2019, EMBL - European Bioinformatics Institute
Distributed under the PLv3 License
See accompanying file LICENSE.txt or copy at http://www.gnu.org/licenses/gpl-3.0.html
Paper website: http://www.ebi.ac.uk/~iorio/PLoS_ONE_Submission

References

[1] Lamb,J. et al. (2006) The Connectivity Map: using gene-expression signatures to connect small molecules, genes, and disease. Science, 313, 1929.

[2] Iorio,F. et al. (2010) Discovery of drug mode of action and drug repositioning from transcriptional responses. Proceedings of the National Academy of Sciences, 107, 14621.

See Also

est_emp_Cs

Examples

```
## loading prototype ranked lists for the connectivity map [1] drugs
load('DATA/DRUG_PRLs.ro')

## loading functions and data objects needed to perform iterative connectivity mapping
source('CODE/ITERATIVE_CMAPPING_library.R')

## generating optimal signature for valproic acid (a histone deacetylase inhibitor) [2]
vaSig<-DeriveSingleSignature(seed='valproic_acid')

## converting signature format
vaSig<-list(UP=as.character(vaSig$seedUPreg$ProbeSets),
            DOWN=as.character(vaSig$seedDOWNreg$ProbeSets))
```

```
## estimating connection scores null distribution for the valproic acid siganture
## by executing 10000 permutation of the drug prototype ranked lists
vaNull<-est_emp_Cs(vaSig,nt=10000,DRUG_PRLs)

## computing the connection score of the prototype ranked list of trichostatin A
## (another histone deacetylase inhibitor) to the valproic acid optimal signature
cs<-cMap_CS(DRUG_PRLs[,'trichostatin_A'],vaSig)

## computing empirical p-value of the obtained connection score
pnormmix(cs,vaNull)
```

qES *Quick Enrichment Score*

Description

This function performs unweighted gene set enrichment analysis (GSEA) [1] by querying a genome-wide ranked list of genes with an input gene signature. It also visualise the obtained running sum.

Usage

```
qES(RANKEDLIST, REGULON, display = TRUE, returnRS = FALSE)
```

Arguments

`RANKEDLIST`	A string vector containing a genome-wide ranked list of genes sorting according to their differential expression, in decreasing order
`REGULON`	A signature of genes (i.e. a subset of the genes contained in `RANKEDLIST`)
`display`	A boolean parameter specifying if the obtained running sum should be visualised or not (default = TRUE)
`returnRS`	A boolean parameter specifying if the obtained running sum should be returned as vector of doubles (default = FALSE)

Value

The obtained enrichment score or (if `returnRS == TRUE`) a structure containing the following objects:

`ES`	The obtained enrichment score
`RS`	A numerical vector with same length of `RANKEDLIST` containing the obtained running sum
`POSITION`	The index position of the genes in `REGULON` along the list contained in `RANKEDLIST`
`PEAK`	The index position at which the running sum in `RS` reaches the maximal divergence from zero

Author(s)

Francesco Iorio (iorio@ebi.ac.uk)
Copyright (c) 2014 - 2019, EMBL - European Bioinformatics Institute
Distributed under the PLv3 License
See accompanying file LICENSE.txt or copy at http://www.gnu.org/licenses/gpl-3.0.html
Paper website: http://www.ebi.ac.uk/~iorio/PLoS_ONE_Submission

Refer ences

[1] Subramanian,A. et al. (2005) Gene set enrichment analysis: a knowledge-based approach for interpreting genome-wide expression profiles. Proceedings of the National Academy of Sciences of the United States of America, 102, 15545.

[2] Garnett,M.J. et al. (2012) Systematic identification of genomic markers of drug sensitivity in cancer cells. Nature, 483, 570-575.

Examples

```
## Loading the genome wide ranked lists of the GDSC [2] cell lines,
## where the genes are sorted according to their basal expression statistics
load('DATA/GDSC_basal_ELstats_rankedLists.ro')

## select a ranked list
rankedList<-gdsc_basal_ELstats_rankedLists[,1]

## selecting a random gene signature
signature<-gdsc_basal_ELstats_rankedLists[sample(1:5000,200),1]

## computing the enrichment score and visualising the obtained running sum
qES(rankedList,signature)
```

Index

∗Topic GSEA
cMap_CS, 2
est_emp_Cs, 6
pnormmix, 9
qES, 10
∗Topic connection scores
cMap_CS, 2
combine_2CS, 4
combine_3CS, 5
CS, 1
est_emp_Cs, 6
pnormmix, 9
qES, 10
∗Topic significance
est_emp_Cs, 6
pnormmix, 9

cMap_CS, 2
combine_2CS, 4
combine_3CS, 5
CS, 1

est_emp_Cs, 6

getDrugName, 7
getDrugTarget, 8

pnormmix, 9

qES, 10

Iterative network guided cMapping and validation

Supplementary Material and Methods - Supplementary Code: ITERATIVE_CMAPPING

This document describes functions, scripts and data objects used in the software enclosed to the paper entitled *A semi-supervised approach for refining transcriptional signatures of drug response and repositioning predictions*, by Francesco Iorio et al, submitted as research paper to PLoS ONE.

Copyright (c) 2014 – 2019, EMBL - European Bioinformatics Institute
Author: Francesco Iorio (iorio@ebi.ac.uk)
Distributed under the GPLv3 License.
See accompanying file LICENSE.txt or copy at http://www.gnu.org/licenses/gpl-3.0.html
Paper website: http://www.ebi.ac.uk/~iorio/PLoS_ONE_Submission

April 30, 2014

`DNquery` *Querying the drug network with a seed compound*

Description

This function queries the drug network described in [1] for compounds whose consensual trascriptional response is similar to that of a given one (i.e. the seed compound).
This set of compounds is called the seed neighborhood. Once the seed neighborhood is computed an enrichment analysis for over-represented drug communities (identified in [1]) is also performed

Usage

```
DNquery(seed = "paclitaxel", distTh = 0.8065, printToFile = FALSE)
```

Arguments

`seed`	String specifying the name of the compound to be used as seed (default = 'paclitaxel')
`distTh`	The distance threshold below which the transcriptional response of two compounds should be considered significantly similar (default = 0.8065 as heuristically determined in [1])
`printToFile`	A boolean parameter specifying if the output of this function should be stored in a tab delimited txt file (default = FALSE). If TRUE then a file (whose name is `$$_DN_neighborhood`, wher `$$` is the name of the seed compound) is created in the `~/OUTPUT` directory (where ~ is the working directory)

Value

A data frame with a row for each of the identified seed neighbors, with the following columns:

`D`	Distance between the seed compound and the compound specified by the row
`quantile perc`	Percentile where the drug distance falls when sorting all the distances in decreasing order
`Drug`	Name of the seed neighboring drug
`C id`	Numerical identifier of the community containing the drug under consideration

`order` The neighborhood order (i.e. a neighbor of order K contains the K closest to the seed neighbors according the distance specified in `D`)

`C occ` Community occurrence = how many drugs belonging to the community whose identifier is specified in `id` are observed in the neighborhood of order K, where K is the row number

`C card` Community cardinality = how many drugs are contained in the community in the drug network described in [1]

`Total #drugs` Total number of drugs contained in the drug network described in [1]

`C Overrep p-val`
Probability of observing by chance the number of drugs specified in `C Occ`, in a neighborhood whose order is specified in `order`, given the background populations specified in `C card` and `Total #drugs`

`Adj p-val` The p-value described above, after correction for multiple hypothesis testing

`MOAs` The modes of action (or drug features) enriched in the drug community specified in `C id`

Author(s)

Francesco Iorio (iorio@ebi.ac.uk)
Copyright (c) 2014 - 2019, EMBL - European Bioinformatics Institute
Distributed under the PLv3 License
See accompanying file LICENSE.txt or copy at http://www.gnu.org/licenses/gpl-3.0.html
Paper website: http://www.ebi.ac.uk/~iorio/PLoS_ONE_Submission

References

[1] Iorio,F. et al. (2010) Discovery of drug mode of action and drug repositioning from transcriptional responses. Proceedings of the National Academy of Sciences, 107, 14621.

Examples

```
## querying the drug network for the neighbors of daunorubicin (a topoisomerase inhibitor)
NN<-DNquery('daunorubicin')

## visualising the first 10 neighbors of daunorubicin
print(NN[1:10,])
```

`DeriveConsistentSignature` *Computing consistent signatures*

Description

This function computes signatures of genes that are consistently up- (resp. down-) regulated when considering the optimal signature [1] of a seed compound and the prototype ranked lists of other user defined connectivity map [2] compounds

Usage

```
DeriveConsistentSignature(seed = "paclitaxel",
                          otherCompounds = c("MG-132", "celastrol", "5224221"),
                          PTH = 30, FUZZYNESS = 2, printToFile = FALSE)
```

Arguments

`seed`	A string specifing the name of the connectivity map [2] drug that should be used as seed
`otherCompounds`	A list of strings specifying the names of the other compounds whose prototype ranked list [1] should be checked for consistency with the optimal signature of the seed
`PTH`	The expression percentile that should be considered when building the consistent signature (see the material and methods of our manuscript for further details)
`FUZZYNESS`	The number of other compounds that should satisfy the consistency
`printToFile`	A boolean parameter specifying if the output of this function should be stored in two tab delimited txt file (default = FALSE), respectively for the up- and the down-regulated part of the signature. If TRUE then two files, whose name will be `$_$$_consistentSignatureUP` (resp. `$_$$_consistentSignatureDOWN`), where `$` is the name of the seed compound and `$$` is a string composed by the other compound names, are created in the `~/OUTPUT` directory (where ~ is the working directory)

Value

A list containing two data frames (`seedUPreg` and `seedDOWNreg`). The first column of these data frame contains the probe-set identifiers in the up-regulated (resp. down-regulated) part of the consistent signature. The following columns contain the percentile in which each probe-set falls along the prototype ranked list [1] of the seed and the other compounds.

Author(s)

Francesco Iorio (iorio@ebi.ac.uk)
Copyright (c) 2014 - 2019, EMBL - European Bioinformatics Institute
Distributed under the PLv3 License
See accompanying file LICENSE.txt or copy at http://www.gnu.org/licenses/gpl-3.0.html
Paper website: http://www.ebi.ac.uk/~iorio/PLoS_ONE_Submission

References

[1] Iorio,F. et al. (2010) Discovery of drug mode of action and drug repositioning from transcriptional responses. Proceedings of the National Academy of Sciences, 107, 14621.

[2] Lamb,J. et al. (2006) The Connectivity Map: using gene-expression signatures to connect small molecules, genes, and disease. Science, 313, 1929.

Examples

```
DeriveConsistentSignature(seed='paclitaxel',otherCompounds=c('MG-132','celastrol'))
```

DeriveInConsistentSignature *Computing inconsistent signatures*

Description

This function computes signatures of genes that are inconsistently up- (resp. down-) regulated when considering the optimal signature [1] of a seed compound and the prototype ranked lists of other user defined connectivity map [2] compounds

Usage

```
DeriveInConsistentSignature(seed = "paclitaxel",
                            otherCompounds = c("MG-132", "celastrol", "5224221"),
                            PTH = 30, FUZZYNESS = 2, printToFile = FALSE)
```

Arguments

`seed`	A string specifing the name of the connectivity map [2] drug that should be used as seed
`otherCompounds`	A list of strings specifying the names of the other compounds whose prototype ranked list [1] should be checked for inconsistency with the optimal signature of the seed
`PTH`	The expression percentile that should be considered when building the inconsistent signature (see the material and methods of our manuscript for further details)
`FUZZYNESS`	The number of other compounds that should satisfy the inconsistency
`printToFile`	A boolean parameter specifying if the output of this function should be stored in two tab delimited txt file (default = FALSE), respectively for the up- and the down-regulated part of the signature. If TRUE then two files, whose name will be `$_$$_inconsistentSignatureUP` (resp. `$_$$_inconsistentSignatureDOWN`), where `$` is the name of the seed compound and `$$` is a string composed by the other compound names, are created in the `~/OUTPUT` directory (where ~ is the working directory)

Value

A list containing two data frames (`seedUPreg` and `seedDOWNreg`). The first column of these data frame contains the probe-set identifiers in the up-regulated (resp. down-regulated) part of the consistent signature. The following columns contain the percentile in which each probe-set falls along the prototype ranked list [1] of the seed and the other compounds.

Author(s)

Francesco Iorio (iorio@ebi.ac.uk)
Copyright (c) 2014 - 2019, EMBL - European Bioinformatics Institute
Distributed under the PLv3 License
See accompanying file LICENSE.txt or copy at http://www.gnu.org/licenses/gpl-3.0.html
Paper website: http://www.ebi.ac.uk/~iorio/PLoS_ONE_Submission

References

[1] Iorio,F. et al. (2010) Discovery of drug mode of action and drug repositioning from transcriptional responses. Proceedings of the National Academy of Sciences, 107, 14621.

[2] Lamb,J. et al. (2006) The Connectivity Map: using gene-expression signatures to connect small molecules, genes, and disease. Science, 313, 1929.

Examples

```
DeriveInConsistentSignature(seed='paclitaxel',otherCompounds=c('MG-132','celastrol'))
```

`DeriveMSTSignature` *Computing inconsistent signatures (less stringently)*

Description

This function computes signatures of genes that are inconsistently up- (resp. down-) regulated when considering the prototype ranked lists [1] of a seed compound and those of other user defined connectivity map [2] compounds

Usage

```
DeriveMSTSignature(seed = "paclitaxel",
                   otherCompounds = c("albendazole", "fenbendazole",
                                      "nocodazole", "parbendazole"),
                   PTH = 25, FUZZYNESS = 4, printToFile = FALSE)
```

Arguments

`seed`	A string specifing the name of the connectivity map [2] drug that should be used as seed
`otherCompounds`	A list of strings specifying the names of the other compounds whose prototype ranked list [1] should be checked for inconsistency with that of the seed
`PTH`	The expression percentile that should be considered when building the inconsistent signature (see the material and methods of our manuscript for further details)
`FUZZYNESS`	The number of other compounds that should satisfy the inconsistency
`printToFile`	A boolean parameter specifying if the output of this function should be stored in two tab delimited txt file (default = FALSE), respectively for the up- and the down-regulated part of the signature. If TRUE then two files, whose name will be `$_$$_MST_UP` (resp. `$_$$_MST_DOWN`), where `$` is the name of the seed compound and `$$` is a string composed by the other compound names, are created in the `~/OUTPUT` directory (where ~ is the working directory)

Value

A list containing two data frames (`seedUPreg` and `seedDOWNreg`). The first column of these data frame contains the probe-set identifiers in the up-regulated (resp. down-regulated) part of the inconsisten signature. The following columns contain the percentile in which each probe-set falls along the prototype ranked list [1] of the seed and the other compounds.

Author(s)

Francesco Iorio (iorio@ebi.ac.uk)
Copyright (c) 2014 - 2019, EMBL - European Bioinformatics Institute
Distributed under the PLv3 License
See accompanying file LICENSE.txt or copy at http://www.gnu.org/licenses/gpl-3.0.html
Paper website: http://www.ebi.ac.uk/~iorio/PLoS_ONE_Submission

References

[1] Iorio,F. et al. (2010) Discovery of drug mode of action and drug repositioning from transcriptional responses. Proceedings of the National Academy of Sciences, 107, 14621.

[2] Lamb,J. et al. (2006) The Connectivity Map: using gene-expression signatures to connect small molecules, genes, and disease. Science, 313, 1929.

Examples

```
DeriveMSTSignature(seed='paclitaxel',otherCompounds=c('albendazole','nocodazole'),FUZZYNESS=2)
```

`DeriveSingleSignature` *Computing single drug optimal signatures*

Description

This function computes the optimal signature (as defined in [1]) for a compound contained in the connectivity map dataset [2]

Usage

```
DeriveSingleSignature(seed = "paclitaxel")
```

Arguments

`seed`	A string specifying the name of the connectivity map compound whose optimal signature should be computed (default = 'paclitax el')

Value

A list containing two data frames (`seedUPreg` and `seedDOWNreg`). The first column of these data frame contains the probe-set identifiers in the up-regulated (resp. down-regulated) part of the optimal signature. The second column contains the percentile in which each probe-set falls along the prototype ranked list [1] of the compound under consideration.

Author(s)

Francesco Iorio (iorio@ebi.ac.uk)
Copyright (c) 2014 - 2019, EMBL - European Bioinformatics Institute
Distributed under the PLv3 License
See accompanying file LICENSE.txt or copy at http://www.gnu.org/licenses/gpl-3.0.html
Paper website: http://www.ebi.ac.uk/~iorio/PLoS_ONE_Submission

References

[1] Iorio,F. et al. (2010) Discovery of drug mode of action and drug repositioning from transcriptional responses. Proceedings of the National Academy of Sciences, 107, 14621.

[2] Lamb,J. et al. (2006) The Connectivity Map: using gene-expression signatures to connect small molecules, genes, and disease. Science, 313, 1929.

Examples

```
DeriveSingleSignature(seed='metformin')
```

`percHeatMaps` *Visualising heatmaps of expression percentiles*

Description

This function visualise the expression percentiles of a set of genes along the prototype ranked lists [1] of a seed drug and those of other user defined compounds

Usage

```
percHeatMaps(probes, seed, otherCompounds, printToFile = FALSE)
```

Arguments

`probes`	A string vector containing the probe-set identifiers whose percentile should be visualised
`seed`	A string specifying the name of the seed compound
`otherCompounds`	A string vector containing the names of the other compounds in the connectivity map [2] dataset
`printToFile`	A boolean parameter specifying if the heatmap produced by this function should be stored in a png file (default = FALSE). If TRUE then a file, whose name will be `$_$$_percHeatMap.png`, where `$` is the name of the seed compound and `$$` is a string composed by the other compound names, will be created in the `~/OUTPUT` directory (where ~ is the working directory)

Details

For usage examples see the pipeline described at
http://www.ebi.ac.uk/~iorio/PLoS_ONE_Submission/iterativeCmappingPL/IterativeCmappingPipeline.html

Author(s)

Francesco Iorio (iorio@ebi.ac.uk)
Copyright (c) 2014 - 2019, EMBL - European Bioinformatics Institute
Distributed under the PLv3 License
See accompanying file LICENSE.txt or copy at http://www.gnu.org/licenses/gpl-3.0.html
Paper website: http://www.ebi.ac.uk/~iorio/PLoS_ONE_Submission

References

[1] Iorio,F. et al. (2010) Discovery of drug mode of action and drug repositioning from transcriptional responses. Proceedings of the National Academy of Sciences, 107, 14621.

[2] Lamb,J. et al. (2006) The Connectivity Map: using gene-expression signatures to connect small molecules, genes, and disease. Science, 313, 1929.

---

plotRunningSums *Visualising running sums for consistent/inconsistent signatures*

Description

This function visualise the enrichment score [1] running sums of two different signatures along the prototype ranked lists [2] of a seed compound and those of other user defined connectivity map drugs [3]

Usage

```
plotRunningSums(consistentSigTable,
                inconsistentSigTable,
                seed = "paclitaxel",
                otherCompounds = c("MG-132", "celastrol", "5224221"),
                printToFile = FALSE)
```

Arguments

consistentSigTable
: A signature generated by the function `DeriveConsistentSignature`

inconsistentSigTable
: A signature generated by the function `DeriveInConsistentSignature`

seed
: A string specifying the name of the seed compound

otherCompounds
: A string vector containing the names of the other compounds in the connectivity map [3] dataset

printToFile
: A boolean parameter specifying if the plots generated by this function should be stored in a png file (default = FALSE). If TRUE then a file, whose name will be `$_$$_RS.png`, where `$` is the name of the seed compound and `$$` is a string composed by the other compound names, will be created in the `~/OUTPUT` directory (where ~ is the working directory)

Details

For usage examples see the pipeline described at
http://www.ebi.ac.uk/~iorio/PLoS_ONE_Submission/iterativeCmappingPL/IterativeCmappingPipeline.html

Author(s)

Francesco Iorio (iorio@ebi.ac.uk)
Copyright (c) 2014 - 2019, EMBL - European Bioinformatics Institute
Distributed under the PLv3 License
See accompanying file LICENSE.txt or copy at http://www.gnu.org/licenses/gpl-3.0.html
Paper website: http://www.ebi.ac.uk/~iorio/PLoS_ONE_Submission

References

[1] Subramanian,A. et al. (2005) Gene set enrichment analysis: a knowledge-based approach for interpreting genome-wide expression profiles. Proceedings of the National Academy of Sciences of the United States of America, 102, 15545.

[2] Iorio,F. et al. (2010) Discovery of drug mode of action and drug repositioning from transcriptional responses. Proceedings of the National Academy of Sciences, 107, 14621.

[3] Lamb,J. et al. (2006) The Connectivity Map: using gene-expression signatures to connect small molecules, genes, and disease. Science, 313, 1929.

`plotRunningSumsMST` *Visualising running sums for (less stringently) inconsistent signatures*

Description

This function visualise the enrichment score [1] running sums of a given signature along the prototype ranked lists [2] of a seed compound and those of other user defined connectivity map drugs [3]

Usage

```
plotRunningSumsMST(MSTsignatureTable,
                   seed = "paclitaxel",
                   otherCompounds = c("albendazole", "fenbendazole",
                                      "nocodazole", "parbendazole"),
                   printToFile = FALSE)
```

Arguments

`MSTsignatureTable`
: A signature generated by the function `DeriveMSTSignature`

`seed`
: A string specifying the name of the seed compound

`otherCompounds`
: A string vector containing the names of the other compounds in the connectivity map [3] dataset

`printToFile`
: A boolean parameter specifying if the plots generated by this function should be stored in a png file (default = FALSE). If TRUE then a file, whose name will be `$_$$_RS.png`, where `$` is the name of the seed compound and `$$` is a string composed by the other compound names, will be created in the `~/OUTPUT` directory (where ~ is the working directory)

Details

For usage examples see the pipeline described at http://www.ebi.ac.uk/~iorio/PLoS_ONE_Submission/iterativeCmappingPL/IterativeCmappingPipeline.html

Author(s)

Francesco Iorio (iorio@ebi.ac.uk)
Copyright (c) 2014 - 2019, EMBL - European Bioinformatics Institute
Distributed under the PLv3 License
See accompanying file LICENSE.txt or copy at http://www.gnu.org/licenses/gpl-3.0.html
Paper website: http://www.ebi.ac.uk/~iorio/PLoS_ONE_Submission

References

[1] Subramanian,A. et al. (2005) Gene set enrichment analysis: a knowledge-based approach for interpreting genome-wide expression profiles. Proceedings of the National Academy of Sciences of the United States of America, 102, 15545.

[2] Iorio,F. et al. (2010) Discovery of drug mode of action and drug repositioning from transcriptional responses. Proceedings of the National Academy of Sciences, 107, 14621.

[3] Lamb,J. et al. (2006) The Connectivity Map: using gene-expression signatures to connect small molecules, genes, and disease. Science, 313, 1929.

Index

∗Topic **signature derivation**
DeriveConsistentSignature, 2
DeriveInConsistentSignature, 4
DeriveMSTSignature, 5
DeriveSingleSignature, 6
∗Topic **visualisation**
percHeatMaps, 7
plotRunningSums, 8
plotRunningSumsMST, 9

DeriveConsistentSignature, 2
DeriveInConsistentSignature, 4
DeriveMSTSignature, 5
DeriveSingleSignature, 6
DNquery, 1

percHeatMaps, 7
plotRunningSums, 8
plotRunningSumsMST, 9

Iterative network guided cMapping and validation

Supplementary Material and Methods - Supplementary Code: GDSC_BASAL_EXP_PREPROCESSING

This document describes functions, scripts and data objects used in the software enclosed to the paper entitled *A semi-supervised approach for refining transcriptional signatures of drug response and repositioning predictions*, by Francesco Iorio et al, submitted as research paper to PLoS ONE.

Copyright (c) 2014 – 2019, EMBL - European Bioinformatics Institute
Author: Francesco Iorio (iorio@ebi.ac.uk)
Distributed under the GPLv3 License.
See accompanying file LICENSE.txt or copy at http://www.gnu.org/licenses/gpl-3.0.html
Paper website: http://www.ebi.ac.uk/~iorio/PLoS_ONE_Submission

April 28, 2014

`EL_statistic` *Basal expression level statistics generation for a single gene*

Description

This function normalises the pre-processed expression signal of given gene across multiple samples, by estimating the density function of its expression first, then computing expression level scores as described in the supplementary methods of our manuscript.

Usage

```
EL_statistic(expression_pattern, ret.pvals = FALSE)
```

Arguments

`expression_pattern`
: A numerical vector containing the pre-processed basal expression profiles of the a gene across m samples

`ret.pvals`
: A bolean parameter specifying whether cumulative probabilities should be returned for all the samples

Value

A numerical vector containing the basal expression level statistics for the input gene or a list containing this vector and the vector of cumulative probabilities for all the samples

Author(s)

Francesco Iorio (iorio@ebi.ac.uk)
Copyright (c) 2014 - 2019, EMBL - European Bioinformatics Institute
Distributed under the PLv3 License
See accompanying file LICENSE.txt or copy at http://www.gnu.org/licenses/gpl-3.0.html
Paper website: http://www.ebi.ac.uk/~iorio/PLoS_ONE_Submission

Examples

```
## loading the pre-processd basal expression dataset of the GDSC [1] cell lines
load('DATA/GDSC_basalEXP.ro')

## visualising histograms of the expression level of "CYFIP2" across all the cell lines
hist(basalEXP["CYFIP2",],main="CYFIP2")

## computing expression level statistics for the selected 20 genes across all the cell lines
elevels<-EL_statistic(basalEXP["CYFIP2",])

hist(elevels,main="CYFIP2")
```

EL_statistics *Basal expression level statistics generation for multiple genes*

Description

This function normalises the pre-processed expression signal of multiple genes across multiple samples, by estimating the density function of their expression first, then computing expression level scores as described in the supplementary methods of our manuscript.

Usage

```
EL_statistics(expression_data, show_progress = TRUE)
```

Arguments

`expression_data`
An n x m double matrix, containing the pre-processed basal expression profiles of the n genes across m samples with row names corresponding to gene symbols and column names correspond to sample identifiers

`show_progress` A boolean parameter specifying if a progress bar should be visualised (default = TRUE)

Value

An n x m double matrix, containing the expression level statistics of the n genes across m samples in the input matrix, with row names corresponding to gene symbols and column names correspond to sample identifiers.

Author(s)

Francesco Iorio (iorio@ebi.ac.uk)
Copyright (c) 2014 - 2019, EMBL - European Bioinformatics Institute
Distributed under the PLv3 License
See accompanying file LICENSE.txt or copy at http://www.gnu.org/licenses/gpl-3.0.html
Paper website: http://www.ebi.ac.uk/~iorio/PLoS_ONE_Submission

Refer ences

[1] Garnett,M.J. et al. (2012) Systematic identification of genomic markers of drug sensitivity in cancer cells. Nature, 483, 570-575.

Examples

```
## loading the pre-processd basal expression dataset of the GDSC [1] cell lines
load('DATA/GDSC_basalEXP.ro')

## visualising histograms of the expression level of 15 genes across all the cell lines
par(mfrow=c(3,5))
for (i in 1:15){
  hist(basalEXP[i,],main=rownames(basalEXP)[i])
}

## computing expression level statistics for the selected 20 genes across all the cell lines
elevels<-EL_statistics(basalEXP[1:15,])

## visualising histograms of the expression level statistics of the selected 15 genes across all the cell l
par(mfrow=c(3,5))
for (i in 1:15){
  hist(elevels[i,],main=rownames(elevels)[i])
}
```

---

basalRanked_lists — *Computing genome-wide ranked lists of genes from their basal expression level statistics*

Description

This Function turns the genome wide basal expression level statistics into ranked list of genes sorted according to these values

Usage

```
basalRanked_lists(medNorm_basalExp)
```

Arguments

`medNorm_basalExp`
: An n x m double matrix containing the basal expression level statistics of n genes across m samples. Rownames should contains gene symbols and column names should contain sample identifiers.

Value

An n x m dataframe containing, for each sample, the genes sorted according to their basal expression level (in decreasing order).

Author(s)

Francesco Iorio (iorio@ebi.ac.uk)
Copyright (c) 2014 - 2019, EMBL - European Bioinformatics Institute
Distributed under the PLv3 License
See accompanying file LICENSE.txt or copy at http://www.gnu.org/licenses/gpl-3.0.html
Paper website: http://www.ebi.ac.uk/~iorio/PLoS_ONE_Submission

Examples

```
## loading the pre-processd basal expression dataset of the GDSC [1] cell lines
load('DATA/GDSC_basalEXP.ro')

## computing expression level statistics for all the genes (this may take a while)
elevels<-EL_statistics(basalEXP)

## computing ranked lists from expression level statistics for all the genes
rankedLists<-basalRanked_lists(elevels)

## visualising genes in the top 10 positions across the first 5 samples
print(rankedLists[1:10,1:5])
```

Index

∗Topic GDSC preprocessing
basalRanked_lists, 3
EL_statistic, 1
EL_statistics, 2

basalRanked_lists, 3

EL_statistic, 1
EL_statistics, 2

Iterative network guided cMapping and validation

Supplementary Material and Methods - Supplementary Code: SIG_REVERSION

This document describes functions, scripts and data objects used in the software enclosed to the paper entitled *A semi-supervised approach for refining transcriptional signatures of drug response and repositioning predictions*, by Francesco Iorio et al, submitted as research paper to PLoS ONE.

Copyright (c) 2014 – 2019, EMBL - European Bioinformatics Institute
Author: Francesco Iorio (iorio@ebi.ac.uk)
Distributed under the GPLv3 License.
See accompanying file LICENSE.txt or copy at http://www.gnu.org/licenses/gpl-3.0.html
Paper website: http://www.ebi.ac.uk/~iorio/PLoS_ONE_Submission

May 1, 2014

`cell_lines_connected_to_mult_sig`
Identifying cancer cell lines connected to multiple signatures

---

Description

This function identifies cancer cell lines in the GDSC [1] panel whose post-processed basal expression profile is connected to multiple signatures

Usage

```
cell_lines_connected_to_mult_sig(multiple_sig_cs, th = 0.3)
```

Arguments

`multiple_sig_cs`
: Connection scores basal expression profiles of the GDSC [1] cell lines to multiple signatures. A list of connection scores data frames obtained by using the `CS` function

`th`
: False discovery rate threshold. A cell line is connected simoultaneously to the multiple signatures if the false discovery rate of all the connection scores is below this threshold

Details

For usage xamples see the pipeline described at
http://www.ebi.ac.uk/~iorio/PLoS_ONE_Submission/
sigRevPL/SigRevPL.html

Value

A list containing two string vectors: `POS` and `NEG`. The former contains COSMIC [2] identifiers of cell lines positively connected simoultaneously to the multiple signatures, the latter those of the cell lines negatively connected simoulteneously to the multiple signatures.

Author(s)

Francesco Iorio (iorio@ebi.ac.uk)
Copyright (c) 2014 - 2019, EMBL - European Bioinformatics Institute
Distributed under the PLv3 License
See accompanying file LICENSE.txt or copy at http://www.gnu.org/licenses/gpl-3.0.html
Paper website: http://www.ebi.ac.uk/~iorio/PLoS_ONE_Submission

References

[1] Garnett,M.J. et al. (2012) Systematic identification of genomic markers of drug sensitivity in cancer cells. Nature, 483, 570-575

[2] Forbes,S.A. et al. (2011) COSMIC: mining complete cancer genomes in the Catalogue of Somatic Mutations in Cancer. Nucleic Acids Res, 39, D945-50.

`genericTtest` *Generic t-test for drug response differences*

Description

This function implements a simple t-test to assess the extent of difference (and its statistical significance) in drug response across two user defined population of cell lines of the GDSC [1] screening

Usage

```
genericTtest(IC50s,
             ALLscreenedCellLines,
             specific_cell_lines,
             drug = drug_id,
             display = TRUE,
             labels = NULL)
```

Arguments

`IC50s` A matrix of IC50 values contained in the `SCREENING` object

`ALLscreenedCellLines` A string vector containing the COSMIC [2] identifiers of all the cell lines to be included in the test

`specific_cell_lines` A string vector containing the COSMIC [2] identifiers of a subset of cell lines to tested for differences in drug response

`drug` The internal identifiers of a drug

`display` A boolean parameter specifying if a box plot should be plotted (default = TRUE)

`labels` A string vector with the labels to be plotted below the two groups of cell lines. If `display = FALSE` then this parameter is ignored

Value

A list containing the following items:

`PVAL`	The p-value of the performed t-test
`deltaMEAN`	Difference of the mean IC50 values across the two groups of cell lines
`effectSize`	Cohen's d quantifying the effect size of the group/drug-response association
`N1`	Number of cell lines in the first group
`N2`	Number of cell lines in the second group

Author(s)

Francesco Iorio (iorio@ebi.ac.uk)
Copyright (c) 2014 - 2019, EMBL - European Bioinformatics Institute
Distributed under the PLv3 License
See accompanying file LICENSE.txt or copy at http://www.gnu.org/licenses/gpl-3.0.html
Paper website: http://www.ebi.ac.uk/~iorio/PLoS_ONE_Submission

References

[1] Garnett,M.J. et al. (2012) Systematic identification of genomic markers of drug sensitivity in cancer cells. Nature, 483, 570-575

[2] Forbes,S.A. et al. (2011) COSMIC: mining complete cancer genomes in the Catalogue of Somatic Mutations in Cancer. Nucleic Acids Res, 39, D945<e2><80><93>50.

Examples

```
## loading functions and objects needed to retrieve drug names and targets
source("CODE/CONNECTION_SCORES_library.R")

## loading drug annotations
load('DATA/GDSC_DRUG_ANNOTATIONS.ro')

## loading cell line annotations
load('DATA/GDSC_CELL_LINE_ANNOTATIONS.ro')

## loading drug screening data
load("DATA/GDSC_DRUG_SCREENING_DATA.ro")


## selecting areo-digestive-tract cancer cell lines
aero_dig_tract_cancer_cell_lines<-
as.character(MASTER_LIST$COSMIC.ID
[which(MASTER_LIST$GDSC.description_1=="aero_dig_tract")])

## assessing the difference in response to paclitaxel
## across two group of cell lines (aero-digestive-tract cancer vs others)
genericTtest(SCREENING$IC50s,
             rownames(SCREENING$IC50s),
             aero_dig_tract_cancer_cell_lines,
             drug='11',labels=c('aerodig tract','others'))
```

test_pred_ability *Testing the predictive ability of the signatures*

Description

This function evaluates the difference in drug response across two groups of cell lines in the GDSC [1] panel. The first one is composed by cell lines negatively connected to a set of signatures (simoultaneously). The second one contains all the other cell lines in the panel

Usage

```
test_pred_ability(multiple_sig_cs, DRUGS, th = 0.3, mainTitle, display = TRUE)
```

Arguments

`multiple_sig_cs`
: Connection scores of the basal expression profiles of the GDSC [1] cell lines to multiple signatures. A list of connection scores data frames obtained by using the `CS` function

`DRUGS`
: The internal identifiers of the drugs to be tested

`th`
: False discovery rate threshold. A cell line is connected simoultaneously to the multiple signatures if the false discovery rate of all the connection scores is below this threshold

`mainTitle`
: Main title of the resulting figure (if `display = TRUE`)

`display`
: A boolean parameter specifying if a box plot should be plotted (default = TRUE)

Details

For usage xamples see the pipeline described at
http://www.ebi.ac.uk/~iorio/PLoS_ONE_Submission/sigRevPL/SigRevPL.html

Value

A data frame with a row for each tested drug, and the following columns:

`used signature(s)`
: Name of the signature(s) tested

`drug id`
: Internal identifier of the tested drug

`drug`
: Name of the tested drug

`target`
: Target of the tested drug

`p-value`
: P-value of a t-test assessing the extent of difference in drug response when dichotomising the set of cell lines in the GDSC [1] panel in two groups: those negatively connected to the tested signatures and all the others

`deltaMean`
: The difference in average IC50s across the two groups of samples described above

`effectSize`
: The effect size of the t-test

`N2`
: Number of cell lines negatively connected to the tested signatures

`N1`
: Number of cell lines in the rest of the GDSC panel

Author(s)

Francesco Iorio (iorio@ebi.ac.uk)
Copyright (c) 2014 - 2019, EMBL - European Bioinformatics Institute
Distributed under the PLv3 License
See accompanying file LICENSE.txt or copy at http://www.gnu.org/licenses/gpl-3.0.html
Paper website: http://www.ebi.ac.uk/~iorio/PLoS_ONE_Submission

References

[1] Garnett,M.J. et al. (2012) Systematic identification of genomic markers of drug sensitivity in cancer cells. Nature, 483, 570-575

Index

cell_lines_connected_to_mult_sig, 1

genericTtest, 2

test_pred_ability, 4

SOURCE CODE

```
##############################################################################################
#                                                                                            #
#     This code is part of the software enclosed to the paper entitled "A semi-supervised approach for  #
#     refining transcriptional signatures of drug response and repositioning predictions",    #
#     by Francesco Iorio et al, submitted as research paper to PLoS ONE.                     #
#                                                                                            #
#     Copyright (c) 2014 - 2019, EMBL - European Bioinformatics Institute                    #
#     Author: Francesco Iorio (iorio@ebi.ac.uk)                                              #
#     Distributed under the GPLv3 License.                                                   #
#     See accompanying file LICENSE.txt or copy at http://www.gnu.org/licenses/gpl-3.0.html  #
#                                                                                            #
#     Paper website: http://www.ebi.ac.uk/~iorio/PLoS_ONE_Submission                         #
                                                                                             #
##############################################################################################

library(mixtools)

combine_2CS<-function(CS1,CS2,printToFile=FALSE,fn=''){

  connectedDRUGS<-colnames(DRUG_PRLs)

  RESULTS<-
cbind(CS1$CS[connectedDRUGS],CS1$Pval[connectedDRUGS],100*CS1$adjP[connectedDRUGS],CS1$NCS[connectedDRUGS],

CS2$CS[connectedDRUGS],CS2$Pval[connectedDRUGS],100*CS2$adjP[connectedDRUGS],CS2$NCS[connectedDRUGS],
                 rowMeans(cbind(CS1$NCS[connectedDRUGS],CS2$NCS[connectedDRUGS])))
  RESULTS<-RESULTS[order(RESULTS[,9],decreasing=TRUE),]

  colnames(RESULTS)<-c('cons S CS','cons S pvalue','cons S fdr %','cons S NCS',
                       'incons S CS','incons S pvalue','incons S fdr %','incons S NCS',
                       'avg NCS')

  if (printToFile){
    RESULTS<-cbind(rownames(RESULTS),RESULTS)
    colnames(RESULTS)[1]<-'DRUG'

write.table(RESULTS,quote=FALSE,sep='\t',row.names=FALSE,file=paste('OUTPUT/',fn,'_refined_neighborhood.txt',sep=''))

  }
  return(RESULTS)
}
combine_3CS<-function(CS1,CS2,CS3,previousNeighBr='',printToFile=FALSE,fn=''){
  connectedDRUGS<-previousNeighBr

  RESULTS<-cbind(CS1$NCS[connectedDRUGS],
                 CS2$NCS[connectedDRUGS],
                 CS3$NCS[connectedDRUGS],
                 rowMeans(cbind(CS1$NCS[connectedDRUGS],CS2$NCS[connectedDRUGS],CS3$NCS[connectedDRUGS])))

  RESULTS<-RESULTS[order(RESULTS[,4],decreasing=TRUE),]

  colnames(RESULTS)<-c('P/PI cons S NCS',
                       'P/PI incons S NCS',
                       'MST S NCS',
                       'avg NCS')

  if (printToFile){
    RESULTS<-cbind(rownames(RESULTS),RESULTS)
    colnames(RESULTS)[1]<-'DRUG'
    write.table(RESULTS,quote=FALSE,sep='\t',row.names=FALSE,file=paste('OUTPUT/',fn,'_neighborhood.txt',sep=''))
  }
  return(RESULTS)
}
cMap_CS<-function(ranked_list,opsig1,returnRS=FALSE){
  ESUP1<-qES(ranked_list,opsig1$UP,display=FALSE,returnRS=returnRS)
  ESDOWN1<-qES(ranked_list,opsig1$DOWN,display=FALSE,returnRS=returnRS)

  if (returnRS){
    RSUP<-ESUP1$RS
    RSDOWN<-ESDOWN1$RS

    ESUP1<-ESUP1$ES
    ESDOWN1<-ESDOWN1$ES

    TES1<-(ESUP1-ESDOWN1)/2

    return(list(TES=TES1,ESUP=ESUP1,ESDOWN=ESDOWN1,RSUP=RSUP,RSDOWN=RSDOWN))
  }
```

```
  TES1<-(ESUP1-ESDOWN1)/2

  return(TES1)
}

CS<-function(signature,RANKED_LISTS,show_progress=TRUE){

  signature<-list(UP=signature$seedUPreg$ProbeSets,
                  DOWN=signature$seedDOWNreg$ProbeSets)

  ns<-ncol(RANKED_LISTS)

  CS<-rep(NA,ns)
  Pvals<-rep(NA,ns)

  names(CS)<-colnames(RANKED_LISTS)
  names(Pvals)<-colnames(RANKED_LISTS)

  cat('simulating null model\n')
  mixmdl<-est_emp_Cs(signature,10000,RANKED_LISTS,show_progress=show_progress)
  cat('done!\n')

  cat('computing connectivity scores\n')

  if(show_progress){
    pb <- txtProgressBar(min=1,max=ns,style=3)
  }

  for (i in 1:ns){
    CS[i]<-cMap_CS(RANKED_LISTS[,i],signature)
    Pvals[i]<-pnormmix(CS[i], mixmdl)
    if(show_progress){
      setTxtProgressBar(pb, i)
    }
  }

  if(show_progress){
    Sys.sleep(1)
    close(pb)
  }

  NCS<-rep(NA,length(CS))
  NCS[CS>=0]<-CS[CS>=0]/max(mixmdl$mu)
  NCS[CS<0]<--CS[CS<0]/min(mixmdl$mu)

  names(NCS)<-names(CS)

  res<-list(CS=CS,Pval=Pvals,adjP=p.adjust(Pvals,method='fdr'),NCS=NCS)

  cat('Done!\n')

  return(res)
}
qES<-function(RANKEDLIST,REGULON,display=TRUE,returnRS=FALSE){

  REGULON<-intersect(as.character(REGULON),RANKEDLIST)

  HITS<-is.element(RANKEDLIST,REGULON)+0

  hitCases<-cumsum(HITS)
  missCases<-cumsum(1-HITS)

  N<-length(RANKEDLIST)
  NR<-length(REGULON)

  Phit<-hitCases/NR
  Pmiss<-missCases/(N-NR)

  m<-max(abs(Phit-Pmiss))
  t<-which(abs(Phit-Pmiss)==m)

  if (length(t)>1){t<-t[1]}
  peak<-t
  ES<-Phit[t]-Pmiss[t]
  RS<-Phit-Pmiss

  if (display){
    if (ES>=0){c<-"red"}else{c<-"green"}
```

```
    plot(0:N,c(0,Phit-Pmiss),col=c,type="l",xlim=c(0,N),ylim=c(-(abs(ES)+0.5*(abs(ES))),abs(ES)+0.5*
(abs(ES))),xaxs="i",bty="l",axes=FALSE,
         xlab="Gene Rank Position",ylab="Running Sum")
    par(new=TRUE)
    plot(0:N,rep(0,N+1),col='gray',type="l",new=FALSE,xlab="",ylab="",ylim=c(-(abs(ES)+0.5*(abs(ES))),abs(ES)+0.5*
(abs(ES))))
    axis(side=2)

  }

  if (returnRS){
    POSITIONS<-which(HITS==1)
    names(POSITIONS)<-RANKEDLIST[which(HITS==1)]

    POSITIONS<-POSITIONS[order(names(POSITIONS))]
    names(POSITIONS)<-names(POSITIONS)[order(names(POSITIONS))]

    return(list(ES=ES,RS=RS,POSITIONS=POSITIONS,PEAK=t))
  } else {return(ES)}
}

est_emp_Cs<-function(signature,nt,RANKED_LISTS,show_progress=TRUE){
  EMP_CS<-rep(NA,nt)

  ng<-nrow(RANKED_LISTS)

  if (show_progress){
    pb <- txtProgressBar(min=1,max=nt,style=3)
  }

  for (i in 1:nt){
    EMP_CS[i]<-cMap_CS(RANKED_LISTS[sample(1:ng,ng),1],signature)
    if (show_progress){
      setTxtProgressBar(pb, i)
    }
  }
  if(show_progress){
    Sys.sleep(1)
    close(pb)
  }
  mixmdl = normalmixEM(EMP_CS,k=3,verb=FALSE)

  return(mixmdl)
}

pnormmix <- function(x,mixture) {
  lambda <- mixture$lambda
  k <- length(lambda)
  pnorm.from.mix <- function(x,component) {
    if (x>=0){
      lambda[component]*pnorm(-x,mean=-mixture$mu[component],sd=mixture$sigma[component],lower.tail=TRUE)
    }else {
      lambda[component]*pnorm(x,mean=mixture$mu[component],sd=mixture$sigma[component],lower.tail=TRUE)
    }
  }
  pnorms <- sapply(1:k,pnorm.from.mix,x=x)
  return(sum(pnorms))
}

getDrugName<-function(id){
  return(as.character(DRUG_PROPS[id,'DRUG_NAME']))
}
getDrugTarget<-function(id){
  return(as.character(DRUG_PROPS[id,'PUTATIVE_TARGET']))
}
```

```
###########################################################################################################
#                                                                                                         #
#     This code is part of the software enclosed to the paper entitled "A semi-supervised approach for  #
#     refining transcriptional signatures of drug response and repositioning predictions",              #
#by Francesco Iorio et al, submitted as research paper to PLoS ONE.                                      #
#                                                                                                         #
#     Copyright (c) 2014 - 2019, EMBL - European Bioinformatics Institute                                #
#     Author: Francesco Iorio (iorio@ebi.ac.uk)                                                          #
#     Distributed under the GPLv3 License.                                                               #
#     See accompanying file LICENSE.txt or copy at http://www.gnu.org/licenses/gpl-3.0.html            #
#                                                                                                         #
#               Paper website: http://www.ebi.ac.uk/~iorio/PLoS_ONE_submission                          #
#                                                                                                         #
###########################################################################################################


library(pheatmap)

load('DATA/DRUG_DISTANCES.ro')
load('DATA/DRUG_COMMUNITIES.ro')
load('DATA/enrichedMoas.ro')
load('DATA/DRUG_PRLs.ro')
load('DATA/affy_ps_annotation.ro')


DNquery<-function(seed='paclitaxel',distTh=0.8065,printToFile=FALSE){
  neighborDistances<-sort(DRUG_DISTANCES[seed,])
  neighborDistances<-neighborDistances[which(neighborDistances<distTh)]

  seedId<-which(names(neighborDistances)==seed)
  neighborDistances<-neighborDistances[-seedId]

  neighbors<-names(neighborDistances)

  WholeDistanceSet<-c(DRUG_DISTANCES)
  WholeDistanceSet<-WholeDistanceSet[which(WholeDistanceSet>0)]

  quantiles<-rep(NA,length(neighbors))
  names(quantiles)<-neighbors

  drugCommunities<-DRUG_COMMUNITIES[neighbors,1]
  names(drugCommunities)<-neighbors
  communityOccurrence<-rep(NA,length(drugCommunities))
  communityCardinality<-rep(NA,length(drugCommunities))

  flag<-0
  for (i in neighbors){
    flag<-flag+1
    quantiles[i]<-length(which(WholeDistanceSet<=neighborDistances[i]))/length(WholeDistanceSet)*100
    communityOccurrence[flag]<-length(which(drugCommunities[1:flag]==drugCommunities[i]))
    communityCardinality[flag]<-length(which(DRUG_COMMUNITIES[,1]==drugCommunities[i]))
  }

  totalNdrugs<-rep(nrow(DRUG_COMMUNITIES),length(neighbors))

  subNeighOrder<-1:length(neighbors)
  PVALS<-phyper(communityOccurrence-1,communityCardinality,totalNdrugs-communityCardinality,subNeighOrder,lower.tail=FALSE)

  PVALS[which(communityOccurrence<2)]<-NA

  a.pval<-p.adjust(PVALS[!is.na(PVALS)],'fdr')
  ADJ.PVAL<-rep(NA,length(drugCommunities))
  ADJ.PVAL[!is.na(PVALS)]<-a.pval

  MOAs<-enrichedMOAs[as.character(drugCommunities)]

  neighborhood<-
as.data.frame(cbind(neighborDistances,quantiles,neighbors,drugCommunities,subNeighOrder,communityOccurrence,communityCardinality,

                                    ADJ.PVAL,MOAs))



  colnames(neighborhood)<-c('D','quantile %','Drug','C id','order','C Occ','C card','Total #drugs','C Overrep p-val','Adj p-
val','MOAs')

  if(printToFile){
    write.table(neighborhood,quote=FALSE,sep='\t',row.names=FALSE,file=paste('OUTPUT/',seed,'_DN_neighborhood.txt',sep=''))
  }

  return(neighborhood)
}

DeriveSingleSignature<-function(seed='paclitaxel'){

  seedUP<-DRUG_PRLs[1:250,seed]
```

```
  nprobes<-nrow(DRUG_PRLs)

  current_percentile<-100*match(seedUP,DRUG_PRLs[,seed])/nprobes
  percentiles<-current_percentile

  Signature<-cbind(seedUP,percentiles)
  colnames(Signature)[1]<-'ProbeSets'
  rownames(Signature)<-Signature[,1]
  UP<-as.data.frame(Signature)

  seedDOWN<-DRUG_PRLs[nprobes:(nprobes-250+1),seed]

  current_percentile<-100*match(seedDOWN,DRUG_PRLs[,seed])/nprobes
  percentiles<-current_percentile

  Signature<-cbind(seedDOWN,percentiles)
  colnames(Signature)[1]<-'ProbeSets'
  rownames(Signature)<-Signature[,1]

  DOWN<-as.data.frame(Signature)
  return(list(seedUPreg=UP,seedDOWNreg=DOWN))



}
DeriveInConsistentSignature<-function(seed='paclitaxel',otherCompounds=c('MG-
132','celastrol','5224221'),PTH=30,FUZZYNESS=2,printToFile=FALSE){

  seedUP<-DRUG_PRLs[1:250,seed]

  nprobes<-nrow(DRUG_PRLs)

  for (i in 1:length(otherCompounds)){

    current_percentile<-100*match(seedUP,DRUG_PRLs[,otherCompounds[i]])/nprobes

    if (i == 1){
      percentiles<-current_percentile
    }else{
      percentiles<-cbind(percentiles,current_percentile)
    }
  }

  colnames(percentiles)<-otherCompounds
  rownames(percentiles)<-seedUP

  inconsistency<-(rowSums(percentiles>=(100-PTH)))>=FUZZYNESS
  inconsistentSignature<-which(inconsistency)

  inconsistentSignature<-percentiles[inconsistentSignature,]

  seedPercentiles<-100*match(rownames(inconsistentSignature),DRUG_PRLs[,seed])/nprobes

  inconsistentSignature<-cbind(seedPercentiles,inconsistentSignature)
  colnames(inconsistentSignature)[1]<-seed

  inconsistentSignature<-cbind(rownames(inconsistentSignature),inconsistentSignature)
  colnames(inconsistentSignature)[1]<-'ProbeSets'

  UP<-as.data.frame(inconsistentSignature)

  if(printToFile){


write.table(inconsistentSignature,quote=FALSE,sep='\t',row.names=FALSE,file=paste('OUTPUT/',seed,'_',paste(otherCompounds,collaps

                                                                                        '_inconsistentSignatureUP.txt',sep=''))
  }


  seedDOWN<-DRUG_PRLs[nprobes:(nprobes-250+1),seed]


  for (i in 1:length(otherCompounds)){

    current_percentile<-100*match(seedDOWN,DRUG_PRLs[,otherCompounds[i]])/nprobes

    if (i == 1){
      percentiles<-current_percentile
    }else{
      percentiles<-cbind(percentiles,current_percentile)
    }
  }

  colnames(percentiles)<-otherCompounds
  rownames(percentiles)<-seedDOWN
```

```
  inconsistency<-(rowSums(percentiles<=PTH))>=FUZZYNESS
  inconsistentSignature<-which(inconsistency)

  inconsistentSignature<-percentiles[inconsistentSignature,]

  seedPercentiles<-100*match(rownames(inconsistentSignature),DRUG_PRLs[,seed])/nprobes

  inconsistentSignature<-cbind(seedPercentiles,inconsistentSignature)
  colnames(inconsistentSignature)[1]<-seed

  inconsistentSignature<-cbind(rownames(inconsistentSignature),inconsistentSignature)
  colnames(inconsistentSignature)[1]<-'ProbeSets'

  DOWN<-as.data.frame(inconsistentSignature)

  if(printToFile){

write.table(inconsistentSignature,quote=FALSE,sep='\t',row.names=FALSE,file=paste('OUTPUT/',seed,'_',paste(otherCompounds,collaps

                                                                                     '_inconsistentSignatureDOWN.txt',sep=''))
  }

  return(list(seedUPreg=UP,seedDOWNreg=DOWN))


}
DeriveConsistentSignature<-function(seed='paclitaxel',otherCompounds=c('MG-
132','celastrol','5224221'),PTH=30,FUZZYNESS=2,printToFile=FALSE){

  seedUP<-DRUG_PRLs[1:250,seed]

  nprobes<-nrow(DRUG_PRLs)

  for (i in 1:length(otherCompounds)){

    current_percentile<-100*match(seedUP,DRUG_PRLs[,otherCompounds[i]])/nprobes

    if (i == 1){
      percentiles<-current_percentile
    }else{
      percentiles<-cbind(percentiles,current_percentile)
    }
  }

  colnames(percentiles)<-otherCompounds
  rownames(percentiles)<-seedUP

  inconsistency<-(rowSums(percentiles>=(100-PTH)))>=FUZZYNESS
  consistentSignature<-which(!inconsistency)

  consistentSignature<-percentiles[consistentSignature,]

  seedPercentiles<-100*match(rownames(consistentSignature),DRUG_PRLs[,seed])/nprobes

  consistentSignature<-cbind(seedPercentiles,consistentSignature)
  colnames(consistentSignature)[1]<-seed

  consistentSignature<-cbind(rownames(consistentSignature),consistentSignature)
  colnames(consistentSignature)[1]<-'ProbeSets'

  UP<-as.data.frame(consistentSignature)

  if(printToFile){

write.table(consistentSignature,quote=FALSE,sep='\t',row.names=FALSE,file=paste('OUTPUT/',seed,'_',paste(otherCompounds,collapse=

                                                                                   '_consistentSignatureUP.txt',sep=''))
  }


  seedDOWN<-DRUG_PRLs[nprobes:(nprobes-250+1),seed]


  for (i in 1:length(otherCompounds)){

    current_percentile<-100*match(seedDOWN,DRUG_PRLs[,otherCompounds[i]])/nprobes

    if (i == 1){
      percentiles<-current_percentile
    }else{
      percentiles<-cbind(percentiles,current_percentile)
    }
  }
```

```
  colnames(percentiles)<-otherCompounds
  rownames(percentiles)<-seedDOWN

  inconsistency<-(rowSums(percentiles<=PTH))>=FUZZYNESS
  consistentSignature<-which(!inconsistency)

  consistentSignature<-percentiles[consistentSignature,]

  seedPercentiles<-100*match(rownames(consistentSignature),DRUG_PRLs[,seed])/nprobes

  consistentSignature<-cbind(seedPercentiles,consistentSignature)
  colnames(consistentSignature)[1]<-seed

  consistentSignature<-cbind(rownames(consistentSignature),consistentSignature)
  colnames(consistentSignature)[1]<-'ProbeSets'

  DOWN<-as.data.frame(consistentSignature)

  if(printToFile){


write.table(consistentSignature,quote=FALSE,sep='\t',row.names=FALSE,file=paste('OUTPUT/',seed,'_',paste(otherCompounds,collapse=

                                                                                    '_consistentSignatureDOWN.txt',sep=''))
  }

  return(list(seedUPreg=UP,seedDOWNreg=DOWN))




}
DeriveMSTSignature<-
function(seed='paclitaxel',otherCompounds=c('albendazole','fenbendazole','nocodazole','parbendazole'),PTH=25,FUZZYNESS=4,printToF
{

  nprobes<-nrow(DRUG_PRLs)

  UpLim<-round(nprobes*PTH/100)

  seedUP<-DRUG_PRLs[1:UpLim,seed]

  for (i in 1:length(otherCompounds)){

    current_percentile<-100*match(seedUP,DRUG_PRLs[,otherCompounds[i]])/nprobes

    if (i == 1){
      percentiles<-current_percentile
    }else{
      percentiles<-cbind(percentiles,current_percentile)
    }
  }

  colnames(percentiles)<-otherCompounds
  rownames(percentiles)<-seedUP

  inconsistency<-(rowSums(percentiles>=(100-PTH)))>=FUZZYNESS
  MSTsignature<-which(inconsistency)

  MSTsignature<-percentiles[MSTsignature,]

  seedPercentiles<-100*match(rownames(MSTsignature),DRUG_PRLs[,seed])/nprobes

  MSTsignature<-cbind(seedPercentiles,MSTsignature)
  colnames(MSTsignature)[1]<-seed

  MSTsignature<-cbind(rownames(MSTsignature),MSTsignature)
  colnames(MSTsignature)[1]<-'ProbeSets'

  UP<-as.data.frame(MSTsignature)

  if(printToFile){


write.table(MSTsignature,quote=FALSE,sep='\t',row.names=FALSE,file=paste('OUTPUT/',seed,'_',paste(otherCompounds,collapse=','),
                                                                             '_MST_UP.txt',sep=''))
  }


  seedDOWN<-DRUG_PRLs[nprobes:(nprobes-UpLim+1),seed]


  for (i in 1:length(otherCompounds)){

    current_percentile<-100*match(seedDOWN,DRUG_PRLs[,otherCompounds[i]])/nprobes
```

```
    if (i == 1){
      percentiles<-current_percentile
    }else{
      percentiles<-cbind(percentiles,current_percentile)
    }
  }

  colnames(percentiles)<-otherCompounds
  rownames(percentiles)<-seedDOWN

  inconsistency<-(rowSums(percentiles<=PTH))>=FUZZYNESS
  MTDSignature<-which(inconsistency)

  MTDSignature<-percentiles[MTDSignature,]

  seedPercentiles<-100*match(rownames(MTDSignature),DRUG_PRLs[,seed])/nprobes

  MTDSignature<-cbind(seedPercentiles,MTDSignature)
  colnames(MTDSignature)[1]<-seed

  MTDSignature<-cbind(rownames(MTDSignature),MTDSignature)
  colnames(MTDSignature)[1]<-'ProbeSets'

  DOWN<-as.data.frame(MTDSignature)

  if(printToFile){


write.table(MTDSignature,quote=FALSE,sep='\t',row.names=FALSE,file=paste('OUTPUT/',seed,'_',paste(otherCompounds,collapse=','),
                                                                             '_MST_DOWN.txt',sep=''))
  }

  return(list(seedUPreg=UP,seedDOWNreg=DOWN))




}
percHeatMaps<-function(probes,seed,otherCompounds,printToFile=FALSE){

  compounds<-c(seed,otherCompounds)
  nprobes<-nrow(DRUG_PRLs)

  for (i in 1:length(compounds)){

    current_percentile<-100*match(probes,DRUG_PRLs[,compounds[i]])/nprobes

    if (i == 1){
      percentiles<-current_percentile
    }else{
      percentiles<-cbind(percentiles,current_percentile)
    }
  }

  colnames(percentiles)<-compounds



  if (printToFile){
    pheatmap(filename=paste('OUTPUT/',seed,'_',paste(otherCompounds,collapse=','),
                            '_percHeatMap.png',sep=''),border_color=NA,
             percentiles,cluster_rows=FALSE,cluster_cols=FALSE,color=colorRampPalette(colors=c('darkred','white','darkblue'))
(100))
  }else{

pheatmap(percentiles,border_color=NA,cluster_rows=FALSE,cluster_cols=FALSE,color=colorRampPalette(colors=c('darkred','white','dar
(100))
  }

}
plotRunningSums<-function(consistentSigTable,inconsistentSigTable,
                          seed='paclitaxel',otherCompounds=c('MG-132','celastrol','5224221'),
                          printToFile=FALSE){

  if(printToFile){
    png(width=1024,height=300,paste('OUTPUT/',seed,'_',paste(otherCompounds,collapse=','),
                                    '_RS.png',sep=''))
  }

  Consistent_signature1<-list(UP=consistentSigTable$seedUPreg$ProbeSets,
                              DOWN=consistentSigTable$seedDOWNreg$ProbeSets)

  Inconsistent_signature1<-list(UP=inconsistentSigTable$seedUPreg$ProbeSets,
                                DOWN=inconsistentSigTable$seedDOWNreg$ProbeSets)

  compounds<-c(seed,otherCompounds)
  ncompounds<-length(compounds)
```

```
  layout(matrix(c(1:(2*ncompounds),rep(2*ncompounds+1,ncompounds)), ncol=ncompounds, byrow=TRUE), heights=c(4, 4, 2))

  par(mar=c(2,5,4,4))

  for (i in 1:ncompounds){
    if(i==1) {ylab='ES RS consistent S'} else {ylab=''}
    ConsSig_CS<-cMap_CS(DRUG_PRLs[,compounds[i]],Consistent_signature1,returnRS=TRUE)
    plot(ConsSig_CS$RSUP,type='l',ylab=ylab,xlab='',col='darkred',ylim=c(-1,1),lwd=3,main=compounds[i],cex.main=2,cex.lab=1.5)
    peakUP<-which(abs(ConsSig_CS$RSUP)==max(abs(ConsSig_CS$RSUP)))[1]
    peakDOWN<-which(abs(ConsSig_CS$RSDOWN)==max(abs(ConsSig_CS$RSDOWN)))[1]

    par(new=TRUE)
    plot(ConsSig_CS$RSDOWN,type='l',ylab='',xlab='',col='darkblue',ylim=c(-1,1),lwd=2.5,axes=FALSE)
    abline(h=0,lty=2,col='darkgray')
    abline(v=peakUP,lty=3,col='darkred',lwd=3)
    abline(v=peakDOWN,lty=3,col='darkblue',lwd=3)

  }

  for (i in 1:ncompounds){
    if(i==1) {ylab='ES RS inconsistent S'} else {ylab=''}
    InconsSig_CS<-cMap_CS(DRUG_PRLs[,compounds[i]],Inconsistent_signature1,returnRS=TRUE)
    plot(InconsSig_CS$RSUP,type='l',ylab=ylab,xlab='rank
position',col='darkred',ylim=c(-1,1),lwd=3,main=compounds[i],cex.main=2,cex.lab=1.5)
    peakUP<-which(abs(InconsSig_CS$RSUP)==max(abs(InconsSig_CS$RSUP)))[1]
    peakDOWN<-which(abs(InconsSig_CS$RSDOWN)==max(abs(InconsSig_CS$RSDOWN)))[1]

    par(new=TRUE)
    plot(InconsSig_CS$RSDOWN,type='l',ylab='',xlab='',col='darkblue',ylim=c(-1,1),lwd=3,axes=FALSE)
    abline(h=0,lty=2,col='darkgray')
    abline(v=peakUP,lty=3,col='darkred',lwd=3)
    abline(v=peakDOWN,lty=3,col='darkblue',lwd=3)
  }

  plot(0,0,col='white',axes=FALSE,xlab='',ylab='')
  legend('left',col=c('darkred','darkblue'),legend=c('up-regulated part','down-regulated
part'),bty='n',cex=2,horiz=TRUE,lty=1,lwd=3)
  legend('right',col=c('darkred','darkblue'),legend=c('positive peak','negative peak'),bty='n',cex=2,horiz=TRUE,lty=3,lwd=3)

  if(printToFile){
    dev.off()
  }

}
plotRunningSumsMST<-function(MSTsignatureTable,
                             seed='paclitaxel',
                             otherCompounds=c('albendazole','fenbendazole','nocodazole','parbendazole'),
                             printToFile=FALSE){

  if(printToFile){
    png(width=1024,height=300,paste('OUTPUT/',seed,'_',paste(otherCompounds,collapse=','),
                                    '_RS.png',sep=''))
  }

  MST_signature<-list(UP=MSTsignatureTable$seedUPreg$ProbeSets,
                      DOWN=MSTsignatureTable$seedDOWNreg$ProbeSets)


  compounds<-c(seed,otherCompounds)
  ncompounds<-length(compounds)


  layout(matrix(c(1:ncompounds,rep(ncompounds+1,ncompounds)), ncol=ncompounds, byrow=TRUE), heights=c(4, 2))

  par(mar=c(2,5,4,4))

  for (i in 1:ncompounds){
    if(i==1) {ylab='Microtubule Stabiliser Signature'} else {ylab=''}
    MSTsig_CS<-cMap_CS(DRUG_PRLs[,compounds[i]],MST_signature,returnRS=TRUE)
    plot(MSTsig_CS$RSUP,type='l',ylab=ylab,xlab='',col='darkred',ylim=c(-1,1),lwd=3,main=compounds[i],cex.main=2,cex.lab=1.5)
    peakUP<-which(abs(MSTsig_CS$RSUP)==max(abs(MSTsig_CS$RSUP)))[1]
    peakDOWN<-which(abs(MSTsig_CS$RSDOWN)==max(abs(MSTsig_CS$RSDOWN)))[1]

    par(new=TRUE)
    plot(MSTsig_CS$RSDOWN,type='l',ylab='',xlab='',col='darkblue',ylim=c(-1,1),lwd=2.5,axes=FALSE)
    abline(h=0,lty=2,col='darkgray')
    abline(v=peakUP,lty=3,col='darkred',lwd=3)
    abline(v=peakDOWN,lty=3,col='darkblue',lwd=3)

  }

  plot(0,0,col='white',axes=FALSE,xlab='',ylab='')
  legend('left',col=c('darkred','darkblue'),legend=c('up-regulated part','down-regulated
part'),bty='n',cex=2,horiz=TRUE,lty=1,lwd=3)
  legend('right',col=c('darkred','darkblue'),legend=c('positive peak','negative peak'),bty='n',cex=2,horiz=TRUE,lty=3,lwd=3)
```

```
  if(printToFile){
    dev.off()
  }

}
```

```
################################################################################################
#
#
#       This code is part of the software enclosed to the paper entitled "A semi-supervised approach for
#
#   refining transcriptional signatures of drug response and repositioning predictions",
#
#   by Francesco Iorio et al, submitted as research paper to PLoS ONE.
#
#
#
#     Copyright (c) 2014 - 2019, EMBL - European Bioinformatics Institute
#
#     Author: Francesco Iorio (iorio@ebi.ac.uk)
#
#     Distributed under the GPLv3 License.
#
#   See accompanying file LICENSE.txt or copy at http://www.gnu.org/licenses/gpl-3.0.html
#
#
#
#     Paper website: http://www.ebi.ac.uk/~iorio/PLoS_ONE_Submission
#
#
#
################################################################################################

library(sROC)
load('DATA/GDSC_basalEXP.ro')

EL_statistic<-function(expression_pattern,ret.pvals=FALSE){
  ep<-expression_pattern


  CDF<-kCDF(ep,xgrid=ep,adjust=1)
  nep<-CDF$Fhat[match(ep,CDF$x)]

  el<-log(nep/(1-nep))

  if (ret.pvals){
    pvals<-rep(NA,length(nep))
    pvals[el>=0]<-(1-nep[el>=0])
    pvals[el<0]<-nep[el<0]

    return(list(el=el,pvals=pvals))
  }

  return(el)
}

basalRanked_lists<-function(medNorm_basalExp){
  cat('Generating rankings...\n')
  ranks<-apply(medNorm_basalExp,MARGIN=2,FUN='order',decreasing=TRUE)


  nc<-ncol(ranks)
  basal_ranked_lists<-matrix(NA,nrow(medNorm_basalExp),nc)


  for (i in 1:nc){

    basal_ranked_lists[,i]<-rownames(medNorm_basalExp)[ranks[,i]]
  }
  cat('Done!\n')

  colnames(basal_ranked_lists)<-colnames(medNorm_basalExp)
  return(basal_ranked_lists)
}
```

```
EL_statistics<-function(expression_data,show_progress=TRUE){
  ed<-expression_data


  nn<-dim(ed)
  EL<-matrix(NA,nn[1],nn[2])

  cat('Computing Expression Level statistics...\n')
  if(show_progress){pb <- txtProgressBar(min=1,max=nn[1],style=3)}

  for (i in 1:nn[1]){
    EL[i,]<-EL_statistic(ed[i,])
    if(show_progress){setTxtProgressBar(pb, i)}
  }

  if(show_progress){
    Sys.sleep(1)
    close(pb)
  }
  cat('Done!\n')

  rownames(EL)<-rownames(ed)
  colnames(EL)<-colnames(ed)
  return(EL)
}
```

```
##########################################################################################################
#                                                                                                        #
#     This code is part of the software enclosed to the paper entitled "A semi-supervised approach for  #
#     refining transcriptional signatures of drug response and repositioning predictions",              #
#     by Francesco Iorio et al, submitted as research paper to PLoS ONE.                                 #
#                                                                                                        #
#     Copyright (c) 2014 - 2019, EMBL - European Bioinformatics Institute                                #
#     Author: Francesco Iorio (iorio@ebi.ac.uk)                                                          #
#     Distributed under the GPLv3 License.                                                               #
#     See accompanying file LICENSE.txt or copy at http://www.gnu.org/licenses/gpl-3.0.html              #
#                                                                                                        #
#     Paper website: http://www.ebi.ac.uk/~iorio/PLoS_ONE_Submission #                                   #
                                                                                                         #
##########################################################################################################


library(beeswarm)

test_pred_ability<-function(multiple_sig_cs,DRUGS,th=0.30,mainTitle,display=TRUE){

  ndrugs<-length(DRUGS)

  connected_cell_lines<-cell_lines_connected_to_mult_sig(multiple_sig_cs)

  if (display){
    layout(matrix(c(1,1,1,2,3,4), ncol=3, byrow=TRUE),heights=c(1,4))
    par(xpd=NA)
    plot(0,0,col=NA,axes=FALSE,xlab='',ylab='')
    text(0,0,mainTitle,cex=1.5)
  }

  for (i in 1:length(DRUGS)){
    res<-genericTtest(SCREENING$IC50s,ALLscreenedCellLines=rownames(SCREENING$IC50s),display=display,
                 specific_cell_lines=connected_cell_lines$NEG,drug=DRUGS[i],
                 labels=c(paste('predicted\nsensitive'),'others'))
    currentLine<-c(res$PVAL,res$deltaMEAN,res$effectSize,res$N1,res$N2)
    if (i == 1){
      totres<-currentLine

    }else{
      totres<-rbind(totres,currentLine)
    }
  }
  totres<-cbind(DRUGS,as.character(DRUG_PROPS[DRUGS,1]),as.character(DRUG_PROPS[DRUGS,4]),totres)
  totres<-cbind(rep(mainTitle,length(DRUGS)),totres)

  totres<-as.data.frame(totres,row.names=NA)
  colnames(totres)<-c('used signature(s)','drug id','drug','target','p-val','deltaMean','effectSize','N1','N2')
  return(totres)
}
cell_lines_connected_to_mult_sig<-function(multiple_sig_cs,th=0.30){

  nsig<-length(multiple_sig_cs)


  for (i in 1:nsig){
    if (i == 1){
      idxsNEG<-names(which(multiple_sig_cs[[i]]$NCS < 0 & multiple_sig_cs[[i]]$adjP < th))
      idxsPOS<-names(which(multiple_sig_cs[[i]]$NCS > 0 & multiple_sig_cs[[i]]$adjP < th))
    }
    else{
      idxsNEG<-intersect(idxsNEG,names(which(multiple_sig_cs[[i]]$NCS < 0 & multiple_sig_cs[[i]]$adjP < th)))
      idxsPOS<-intersect(idxsNEG,names(which(multiple_sig_cs[[i]]$NCS > 0 & multiple_sig_cs[[i]]$adjP < th)))
    }
  }

  NEG<-intersect(idxsNEG,rownames(SCREENING$IC50s))
  POS<-intersect(idxsPOS,rownames(SCREENING$IC50s))

  return(list(NEG=NEG,POS=POS))
}
genericTtest<-function(IC50s,ALLscreenedCellLines,specific_cell_lines,drug=drug_id,display=TRUE,labels=NULL){

  ALLscreenedCellLines<-intersect(ALLscreenedCellLines,rownames(IC50s))
  specific_cell_lines<-intersect(specific_cell_lines,rownames(IC50s))

  ALLscreenedCellLines<-ALLscreenedCellLines[!is.na(IC50s[ALLscreenedCellLines,as.character(drug)])]
  specific_cell_lines<-specific_cell_lines[!is.na(IC50s[specific_cell_lines,as.character(drug)])]
```

```
  IC50pattern<-IC50s[ALLscreenedCellLines,as.character(drug)]
  names(IC50pattern)<-ALLscreenedCellLines

  if (length(labels)==0){
    labels=c('g1','g2')
  }
  XLAB='GDSC cell lines'

  N1<-length(which(!is.na(IC50pattern[setdiff(ALLscreenedCellLines,specific_cell_lines)])))
  N2<-length(which(!is.na(IC50pattern[specific_cell_lines])))

  if (N1>=2  & N2>=2){


    TT<-t.test(IC50pattern~(is.element(ALLscreenedCellLines,specific_cell_lines)))
    P<-TT$p.value
    DM<-TT$estimate[2]-TT$estimate[1]
    DM<-DM[[1]]


    lx <- N1 - 1
    ly <- N2 - 1
    md  <- abs(DM)        ## mean difference (numerator)


    x<-IC50pattern[setdiff(ALLscreenedCellLines,specific_cell_lines)]
    x<-x[which(!is.na(x))]

    y<-IC50pattern[specific_cell_lines]
    y<-y[which(!is.na(y))]


    csd <- lx * var(x) + ly * var(y)
    csd <- csd/(lx + ly)
    csd <- sqrt(csd)                     ## common sd computation

    cd  <- md/csd                        ## cohen's d

    if(display){


      MAIN<-paste(getDrugName(drug),'\n')



      beeswarm(IC50pattern~
(!is.element(ALLscreenedCellLines,specific_cell_lines)),labels=labels,xlab=XLAB,ylab='log(IC50)',
               col=c(rgb(150,0,255,150,maxColorValue=255),
                     rgb(100,100,100,100,maxColorValue=255)),
                     pch = 16,cex=2,
               main=paste(MAIN,
                          'DM = ',format(DM,digits=2),', Eff.S = ',format(cd,digits=2),
                          ', P = ',format(P,digits=2,scientific=TRUE),
                          sep=''),cex.main=1,corral='wrap',cex.lab=1,cex.names=0.5)


      boxplot(IC50pattern~
(!is.element(ALLscreenedCellLines,specific_cell_lines)),add=TRUE,outline=FALSE,col=NA,boxwex=0.6,names=c('',''),lwd=2)


      Malt<-mean(IC50pattern[which(is.element(ALLscreenedCellLines,specific_cell_lines))])
      Mwt<-mean(IC50pattern[which(!is.element(ALLscreenedCellLines,specific_cell_lines))])

      SDalt<-sd(IC50pattern[which(is.element(ALLscreenedCellLines,specific_cell_lines))])
      SDwt<-sd(IC50pattern[which(!is.element(ALLscreenedCellLines,specific_cell_lines))])

      lines(x=c(0.85,1.15),y=c(Malt,Malt),col='red',lwd=5)
      lines(x=c(1.85,2.15),y=c(Mwt,Mwt),col='red',lwd=5)

      lines(x=c(0.70,1.30),y=c(Malt+SDalt,Malt+SDalt),col='red',lwd=2)
      lines(x=c(0.70,1.30),y=c(Malt-SDalt,Malt-SDalt),col='red',lwd=2)

      lines(x=c(1.70,2.30),y=c(Mwt+SDwt,Mwt+SDwt),col='red',lwd=2)
      lines(x=c(1.70,2.30),y=c(Mwt-SDwt,Mwt-SDwt),col='red',lwd=2)

    }

  }else{
    P<-NA
```

```
    DM<-NA
    cd<-NA
    effectSize<-NA
  }


  return(list(PVAL=P,deltaMEAN=DM,effectSize=cd,N1=N1,N2=N2))
}
```
